# Assessing the Quality and Potential Efficacy of Commercial Extracts of *Rhodiola rosea* L. by Analyzing the Salidroside and Rosavin Content and the Electrophysiological Activity in Hippocampal Long-Term Potentiation, a Synaptic Model of Memory

**DOI:** 10.3389/fphar.2018.00425

**Published:** 2018-05-24

**Authors:** Wilfried Dimpfel, Leonie Schombert, Alexander G. Panossian

**Affiliations:** ^1^Department of Pharmacology, Justus Liebig University Giessen, Giessen, Germany; ^2^NeuroCode AG, Wetzlar, Germany; ^3^EuroPharma USA Inc., Green Bay, WI, United States

**Keywords:** *Rhodiola rosea*, salidroside, rosavin, long-term potentiation, hippocampus, quality control, UPLC

## Abstract

*Rhodiola rosea* L. roots and rhizome extracts are active ingredients in adaptogenic herbal medicinal products (HMP) and dietary supplements for temporary relief of symptoms of stress, such as fatigue and weakness. *R. rosea* extract has a stimulating effect on the CNS, suggesting potential benefits on cognitive functions, memory, learning, and attention. The reproducible efficacy and quality of preparations of the underground parts of *R. rosea* depend on the highly variable content of the active markers, salidroside and rosavin, which affect the quality of HMP and dietary supplements. However, it is not clear which analytical markers are important for assessing the efficacy of *R. rosea* preparations intended for use in aging-induced mild cognitive disorders, such as attenuated memory, attention, and learning. Furthermore, the activity of various commercial *R. rosea* extracts has not been correlated with their content. Here, the biological activities of salidroside, rosavin, and seven commercial extracts of underground parts of *R. rosea* were assessed using a synaptic model of memory: long-term potentiation (LTP) of synaptic transmission in hippocampus slices. A high degree of variation in the content of all active markers was observed. One extract from China lacked rosavin, and there was even variation in the extracts from the Altai geographic region. *In vitro*, rosavin, salidroside and all tested *R. rosea* extracts potentiated electric stimulation of an intra-hippocampal electric circuit, which resulted in higher responses of the pyramidal cells in isolated hippocampus slices. Rosavin was more active at higher concentrations than salidroside; while, salidroside was more effective at lower concentrations. The highest content of both active markers was found in the extracts that were active at the lowest concentrations tested; while, some extracts contained some other compounds that presumably reduced the efficacy due to antagonistic interactions. Standardized content of active markers is necessary for the quality control of herbal preparations containing *R. rosea* extracts, but insufficient for assessment of their potential efficacy. Additional bioassays are needed to assure the reproducible pharmacological activity of *R. rosea* extracts; therefore, the LTP of synaptic transmission in hippocampus slices may serve as a validation tool for the quality control of *R. rosea* extracts.

## Introduction

*Rhodiola rosea* L. [Crassulaceae, syn. *Sedum rhodiola* - DC. *Sedum rosea* - (L.) Scop cop, known as roseroot, rosenroot, golden root, arctic root, orpin rose, rhodiole rougeâtre] (Currier and Ampong-Nyarko, 2015) has a long history as a valuable medicinal plant and has appeared in the Materia Medica of several European countries (Panossian et al., 2010). *Rhodiola rosea* L. roots and rhizome extracts are active ingredients in adaptogenic herbal medicinal products (HMP) and dietary supplements for temporary relief of symptoms of stress, such as fatigue and weakness (Panossian and Wagner, 2005; Panossian and Wikman, 2009, 2010, 2014; EFSA, 2010; Panossian et al., 2010; European Medicine Authority [EMA], 2011). A growing body of evidence has indicated the extract’s potential use in the prevention and treatment of stress- and age-related impairments of cognitive functions and mental disorders (Panossian et al., 2010, 2014; Panossian, 2013; Panossian and Gerbarg, 2016; Amsterdam and Panossian, 2016; Nabavi et al., 2016). The stimulating effects of *R. rosea* on the CNS were demonstrated long ago and suggested there were potential benefits on cognitive functions, memory, learning, and attention (Saratikov et al., 1965, 1978; Marina and Alekseeva, 1968; Kurkin and Zapesochnaya, 1986; Petkov et al., 1986; Marina et al., 1994; Saratikov and Krasnov, 2004). An active compound, named rhodioloside was isolated and identified as salidroside (Aksenova et al., 1968; Saratikov et al., 1968). A pilot study of rhodioloside (syn. salidroside) in 46 healthy human volunteers showed that 2.5 mg salidroside increased attention in cognitive tests 1 h after a single dose was administered in 83% of subjects, compared with 54% of volunteers who were administered placebo (Aksenova et al., 1968). Further studies provided evidence that *R. rosea* and salidroside exhibit neuroprotective activity (Qu et al., 2009; Panossian et al., 2010, 2012; Jacob et al., 2013; Lee et al., 2013), suggesting they may be effective in treating neurodegenerative disorders, such as Alzheimer’s disease (Nabavi et al., 2016).

**FIGURE 1:**
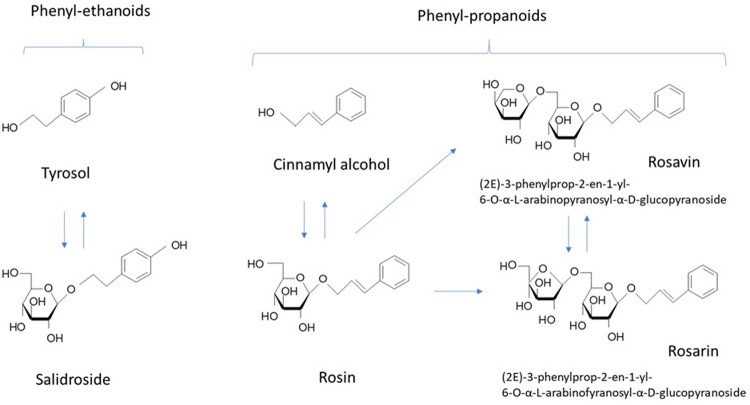
Chemical structures of active markers of *R. rosea* extracts.

Along with salidroside and its aglycone tyrosol (**Figure 1**), cinnamyl alcohol, glycosides, and rosavins (collective name of rosavin, rosarin, and rosin) also exhibited stress-protective (Barnaulov et al., 1986), stimulating, and neurotropic activities in rodents; reduced sleep induced by barbital, hexanal, and chloral hydrate in mice (Sokolov et al., 1985, 1990); increased locomotor activity in mice (Sokolov et al., 1990); and induced anti-depressant-like effects in animal models of depression (Panossian et al., 2008). Salidroside is common for all species of *Rhodiola*, while phenylpropanoids, rosavin, rosarin, and rosin are specific only for *R. rosea* and *R. sachalinensis* (Kurkin et al., 1985; Nakamura et al., 2007; Booker et al., 2016b). Many publications have reported on the neuroprotective and neurotropic activity of salidroside (Sokolov et al., 1985, 1990; Barnaulov et al., 1986; Panossian et al., 2008; Cifani et al., 2010; Lee et al., 2013); while, there is limited evidence supporting the importance of rosavin, the major active marker (Sokolov et al., 1985, 1990; Panossian et al., 2008; Cifani et al., 2010; Marchev et al., 2017). Rosavin was inactive is rats during a behavioral test of binge eating; while, salidroside dose-dependently reduced or abolished binge eating for the period in which it was elicited (Cifani et al., 2010). In another study, salidroside was more effective than rosarin and rosin in inhibiting the expression of IL-1β, and IL-6 in microglial cells, while rosavin was not tested (Lee et al., 2013). Rosavin inhibited the expression of the TNF-related apoptosis-inducing ligand in concanavalin A activated Jurkat T cells, while salidroside was inactive and rosarin had an opposite effect (Marchev et al., 2017).

It is unclear which analytical markers are important for assessing the quality and efficacy of *R. rosea* herbal preparations intended for treating aging-induced mild cognitive disorders, such as attenuated memory, attention, and learning ability. *Rhodiola* preparations are usually standardized for salidroside (1%) and rosavin (3%). The content of active ingredients in herbal preparations depends on many factors, such as the geographic and climate zone it was grown in, which season and under what conditions it was harvested, and how it was dried, extracted, and prepared to give the final dosage form. For example, a high degree of inter-clonal variation was found for all tested constituents (salidroside, tyrosol, rosavin, rosarin, rosin, and cinnamyl alcohol) in six samples of *R. rosea* roots collected in various regions of Norway. The highest variation was found for salidroside and tyrosol, showing inter-clonal variations of 92.8 and 87.8%, respectively (Hellum et al., 2010). Therefore, the preparations obtained by various producers can have quite different active dose levels. Furthermore, the contribution of these active markers to the overall activity of the total extracts was not systematically assessed. It was suggested that these phenolic compounds (rosavin, rosarin, rosin, salidroside/rhodioloside, and tyrosol) have no impact on activity of CYP450 enzymes and do not inhibit CYP3A4, CYP2D6, or CYP1A2 (Hellum et al., 2010; Xu et al., 2013; Thu et al., 2016a). The presence of minor amounts of herbacetin rhamnosides (rhodiosin and rhodionin) may presumably induce inhibition of CYP2D6 (Xu et al., 2013) in some commercial preparations of *Rhodiola* (Thu et al., 2016b, 2017).

It is a challenge to obtain reproducible efficacy and quality of HMP, particularly for preparations of the underground parts of *R. rosea* (Panossian et al., 2010; Ioset et al., 2011; Booker et al., 2016a). There may be unpredictable, complex interactions between the active constituents of the *R. rosea* extracts that affect the regulation of molecular networks playing an important role in cellular and physiological functions of human organisms (Panossian et al., 2014). The pharmacological activity of *R. rosea* crude extract is related to many compounds, such as salidroside, tyrosol, rosavin, and other phenolic compounds (Sokolov et al., 1985, 1990; Barnaulov et al., 1986; Panossian et al., 2008; Lee et al., 2013; Marchev et al., 2017). The batch to batch reproducible content of key active markers and the UPLC fingerprint are not a guaranty of reproducible efficacy and safety. Additional bioassays are required to assure reproducible pharmacological activity of HMP. These bioassays may serve as validation tools for the quality assurance of complex HMP where the total extract contains active pharmaceutical ingredients. In this context, assessment of the correlation between the content of active markers and pharmacological activity of HMP is important. Although the dose-response relationship of salidroside and rosavin and dietary supplements was studied (Cifani et al., 2010; Lee et al., 2013; Marchev et al., 2017), to the our best of our knowledge, the correlation between the content and biological activity of various commercial *R. rosea* extracts has not been investigated.

The aim of our study was to assess the biological activity of a selection of commercial extracts of the underground parts of *R. rosea* and their analytical markers, salidroside and rosavin, in a synaptic model of memory: the long-term potentiation (LTP) of synaptic transmission in the hippocampus (Bliss and Collingridge, 1993). An interesting result using this model was the ability of memantine, a substance used in the treatment of dementia, to increase the population spike amplitude in response to single stimuli (SS) and to increase LTP (Dimpfel, 1995). We used this method earlier for characterization of electrophysiological response of *R. rosea* in hippocampal slices and have demonstrated a concentration-dependent increase of the amplitude of the population spike (Dimpfel et al., 2016b). These results relate very well to previous clinical results where neurophysiological effects of *R. rosea* extract in healthy subjects were characterized (Dimpfel, 2014).

## Materials and Methods

### Test Samples

Seven dry commercial extracts were obtained from different suppliers via the Internet. Our selection strategy was to compare *Rhodiola* extracts containing glycosides of cinnamyl alcohols (rosavins) with extracts containing only tyrosol and its glycoside salidroside. Some of the extracts were from plants grown in the Altai mountains of Siberia. The samples consisted of bulk powders, obtained from water. According to the manufacturers’ certificates of analysis and origin, they were hydroalcoholic extracts of *R. rosea* roots and rhizome (harvest of 2015). Six extracts were preparations containing only root and rhizome powders, while one, SHR-5 contained maltodextrin as a carrier (for details see Supplementary Data S1 and **Table 1**). Our inclusion criteria were that products must be consumed as a solid dose or soft-gel manufactured item. Our exclusion criteria included ethanolic tinctures and raw materials including dried roots, rhizomes, and bulk tinctures. Rhodiola SHR-5 extract has been previously tested for efficacy and safety (Panossian and Wikman, 2014) and was included as a control (i.e., registered or licensed product). The samples of *R. rosea* L. roots and rhizomes extracts were identified by thin-layer chromatography (TLC) and ultra-performance liquid chromatography (UPLC) using salidroside, tyrosol, rosavin, rosarin, rosin, and cinnamyl alcohol as reference standards (Supplementary Data S2). The voucher specimens were deposited in EuroPharma USA Inc.

**Table 1:** Content of active markers (mean ± SD, %) in dry extracts of *R. rosea* rhizome and roots.

Sample code	Tyrosol	Salidroside	Rosarin	Rosin	Rosavin	Cinnamyl alcohol	Phenylethanoids^∗∗^	Phenylpropanoids^∗∗∗^	Total phenolics^§^	Rosavins^∗^
Alt-X	0.38 ± 0.001	1.85 ± 0.001	1.00 ± 0.001	0.51 ± 0.001	0.03 ± 0.002	1.06 ± 0.001	2.23	2.6	4.83	1.54
Alt-S	0.24 ± 0.001	1.91 ± 0.002	1.16 ± 0.001	0.70 ± 0.001	1.33 ± 0.002	0.84 ± 0.001	2.15	4.03	6.18	3.19
Alt-B	0.34 ± 0.000	0.91 ± 0.000	0.71 ± 0.000	0.35 ± 0.000	1.08 ± 0.001	0.13 ± 0.000	1.25	2.27	3.52	2.14
Chi-R	0.33 ± 0.002	2.53 ± 0.001	0.61 ± 0.001	0.31 ± 0.000	1.20 ± 0.002	0.16 ± 0.000	2.86	2.28	5.14	2.12
Chi-S	0.16 ± 0.000	1.14 ± 0.004	0.00	0.00	0.00	0.00	1.3	0	1.3	0
EPR-7	0.46 ± 0.001	3.08 ± 0.005	0.96 ± 0.015	0.40 ± 0.016	3.67 ± 0.012	0.00	3.54	5.03	8.57	5.03
SHR-5	0.15 ± 0.001	2.14 ± 0.036	1.08 ± 0.002	0.59 ± 0.001	3.10 ± 0.003	0.36 ± 0.006	2.29	5.13	7.42	4.77

*^∗^Rosavins is a collective name of phenylpropanoid glycosides rosavin, rosarin, and rosin. ^∗∗^Phenylethanoids: tyrosol and salidroside. ^∗∗∗^Phenylethanoids: rosarin, rosavin, rosin, and cinnamyl alcohol. ^§^Total phenolics: the sum of phenylpropanoids and phenylethanoids. For details related to the country of origin of herbal substance, the manufacturers of the extracts, batch number, year and month of harvest, extraction solvent, evaporation temperature, DERnative, declared content of markers see the Supplementary Data S1.*

### Reference Standards and Solvents

Salidroside, tyrosol, rosavin, rosarin, rosin, and cinnamyl alcohol reference standards were purchased from Phytolab GmbH & Co. KG (Vestenbergsgreuth, Germany) and used for standard curve development. The solvents (water, methanol, and acetonitrile) used for extraction and chromatography were high performance liquid chromatographic grade (Waters Corporation, United States and Merck, Darmstadt, Germany).

### Preparation of the Analytical and Reference Standard Samples

Powdered extract (0.3 g) was dissolved in 25 mL of solvent system water:acetonitrile (90:10) using an ultrasonic bath for 30 min. It was then filtered through a 0.45 μm pore size filter and analyzed by UPLC. Powdered extract (1 g) was dissolved in 10 mL of methanol using an ultrasonic bath for 30 min through a 00H filter. The filtrate was applied (10 μL) to high performance TLC (HPTLC) plates. Stock solutions of the reference standard (2 mg/mL) in methanol was further diluted with methanol to 200 μg/mL, 20 μg/mL, 2 μg/mL, 200 ng/mL, 20 ng/mL, and 2 ng/mL.

### Analytical Methods

The *R. rosea* extracts were analyzed with two basic chromatographic techniques – HPTLC and UPLC. Quantitative analysis of extracts was performed using a UPLC method, validated for linearity (Correlation coefficient *R* > 0.999), repeatability and the levels 50, 100, and 150% (RSD < 5%), intermediate precision at different days and analysts (RSD < 5%), accuracy (recovery in the range from 90 to 110%), selectivity (peak purity angle less than purity threshold with resolution > 2), range from 80 to 120% and robustness (RSD < 2%). Actual results are shown in tabulated form in the Supplementary Data S1, **Table 2**. The limits of detection (LOD) and quantification (LOQ) of UPLC methods were evaluated by calculations based upon the standard deviation of the response (σ) and the slope (S) of calibration curve and the following formulas: LOD = 3.3 σ/S and LOQ = 10 σ/S. The specificity, generally defined as the ability of the UPLC methods to unequivocally assess the sample of interest in the presence of potential interferences, was evaluated in accordance with the new regulatory guideline (USP 25). In addition to the evaluation of the resolution between the sample peak and the next peak, a peak purity test based on photodiode array (PDA) detection was tested to demonstrate that the sample was pure with no co-eluting impurities. Specificity of the TLC method was based on the colors and *R*_f_ value of reference standards bands on the TLC plates visualized as described below.

**Table 2:** Effective concentrations (%) of *Rhodiola* extracts inducing single shock stimulation (SS) and theta burst stimulation (TBS) in the hippocampus slice preparation.

	Salidroside %	Rosavin %	Salidroside + Rosavins,%	SS EC_50_, mg/L	TBS EC_50_, mg/L
Rosavin		100		0.44	0.57
Salidroside	100			0.50	0.49
EPR-7	3.08 ± 0.005	3.67 ± 0.012	8.57	4.81	6.5
Alt-S	1.91 ± 0.002	1.33 ± 0.002	5.34	8.9	7.7
Alt-B	0.91 ± 0.000	1.08 ± 0.001	3.39	15.7	13.8
Alt-X	1.85 ± 0.001	0.03 ± 0.002	3.77	10.7	14.4
Chi-S	1.14 ± 0.002	0.00	1.30	9.8	15.3
SHR-5	2.14 ± 0.036	3.10 ± 0.003	7.06	12.4	16.1
Chi-R	2.53 ± 0.001	1.20 ± 0.002	4.98	14.6	19.5

*Rosavins is a collective name of phenylpropanoid glycosides rosavin, rosarin, and rosin.*

#### UPLC Method

##### Analytical instrumentation and chromatography details

The UPLC fingerprints of the *Rhodiola* extracts were analyzed using a Waters Acquity UPLC system consisting of Quaternary Pumps Manager, Sample Manager, Column Manager, Photodiode Detector, and Empower 3 software (Waters Corporation, Milford, MA, United States). The UPLC column (Waters ACQUITY UPLC BEH C18, column; 100 mm × 2.1 mm i.d., 1.8 μm, Waters Corporation, United States) solvent system was gradually increasing concentrations (2.5 to 100% in 14 min) of acetonitrile in water with a flow rate of 0.6 mL/min at 75°C. The injection volume was 2 μL, detection was at 221 nm (phenylethanoids tyrosol and salidroside) and 252 nm (phenylpropanoids). All quantitative results were calculated per dry weight of the extracts.

#### TLC Method

Test solutions (10 μL) were manually applied on HPTLC plates by a capillary. The solvent system used for HPTLC was ethyl acetate:methanol:water:acetic acid, 90:8:1:8. The bands on the silica gel 60 F254 pre-coated HPTLC plates were visualized by UV light at 254 nm and in the daylight after derivatization with anisaldehyde-sulfuric acid reagent (anisaldehyde:acetic acid:sulfuric acid:methanol, 0.5:10:5:85 v/v/v/v) at 105°C for 10 min. The plates were documented using a “Reprostar” TLC/HPTLC imaging and documentation system for a UV system instrument (CAMAG, Switzerland). Images were captured under UV light at 254 and 366 nm prior to derivatization and in the daylight after derivatization.

### *In Vitro* Assay on Hippocampus Slices

Hippocampus slices were obtained from 48 adult male Sprague-Dawley rats at the age of 40 days (Charles River Wiga, Sulzbach, Germany). Rats were kept under a reversed day/night cycle for 2 weeks prior to the start of the experiments to allow recording of *in vitro* activity from slices during the active phase of their circadian rhythm (Dimpfel et al., 1994). Animals were exsanguinated under ether anesthesia, the brain was removed in total and the hippocampal formation was isolated under a microstereoscopic vision system. The midsection of the hippocampus was fixed to the table of a vibrating microtome (Rhema Labortechnik, Hofheim, Germany) using a cyanoacrylate adhesive, submerged in chilled bicarbonate-buffered saline [artificial cerebrospinal fluid (ACSF): NaCl: 124 mM, KCl: 5 mM, CaCl_2_: 2 mM, MgSO_4_: 2 mM, NaHCO_3_: 26 mM, glucose: 10 mM], and cut into slices of 400 μm thickness. All slices were pre-incubated for at least 1 h in Carbogen saturated ACSF (pH 7.4) in a pre-chamber before use (Dimpfel et al., 1991).

The stimulation of Schaffer Collaterals leads to release of glutamate, resulting in excitation of the postsynaptic pyramidal cells. The result of the electrical stimulation is recorded as a so-called population spike. The amplitude of the resulting population spike represents the number of recruited pyramidal cells. The response of the pyramidal cells to electric stimulation in the form of the amplitude of the population spike indicates activation as increase of the amplitude as reported earlier for Sideritis extract (Dimpfel et al., 2016a) or calming and sedating effects (attenuation of the amplitude). Of special interest is the response to theta burst stimulation (TBS) resulting in LTP, which relates to an increase of time- and space-dependent memory.

During the experiment, the slices were held and treated in a special super-fusion chamber (List Electronics, Darmstadt, Germany) (Haas et al., 1979) at 35°C (Schiff and Somjen, 1985). The preparation was super-fused with ACSF at 180–230 mL/h. Electrical stimulation (200 μA constant current pulses of 200 μs pulse width) of the Schaffer Collaterals within the CA2 area and recording of extracellular field potentials from the pyramidal cell layer of CA1 (Dimpfel et al., 1991) was performed according to conventional electrophysiological methods using the “Labteam” Computer system “NeuroTool” software package (MediSyst GmbH, Linden, Germany). Measurements were performed at 10 min intervals to avoid potentiation mechanisms. Four stimulations, each 20 s apart, were averaged for each time point. After obtaining three stable responses to SS, LTP was induced by applying a TBS. The mean amplitudes of three signals were averaged to give the mean of absolute voltage values (Microvolt) ± standard error (SE) of the mean for four slices for one of the experimental conditions. Four slices were used per day.

### Statistical Analysis

The results are reported as means ± SD (standard deviation) or ±SE for the indicated number of experiments. The significance of differences between samples and controls was determined with one-way independent measures ANOVA, followed by the *post hoc* Tukey’s test for multiple comparisons. The correlations were evaluated using *F*-test. All calculations were performed using GraphPad (San Diego, CA, United States) Prism software (version 3.03) for Windows. GraphPad Prism was also used for supplemental graphs. All statistical tests were two-sided tests with *p*-values < 0.05 regarded as significant (Supplementary Data S3). Wilcoxon–Mann–Whitney *U* test was also used throughout all experimental data for comparison to results obtained by vehicle administration at the particular timing with respect to electrophysiological data (Supplementary Data S4).

## Results

### UPLC and HPTLC Metabolite Profiling

**FIGURE 2:**
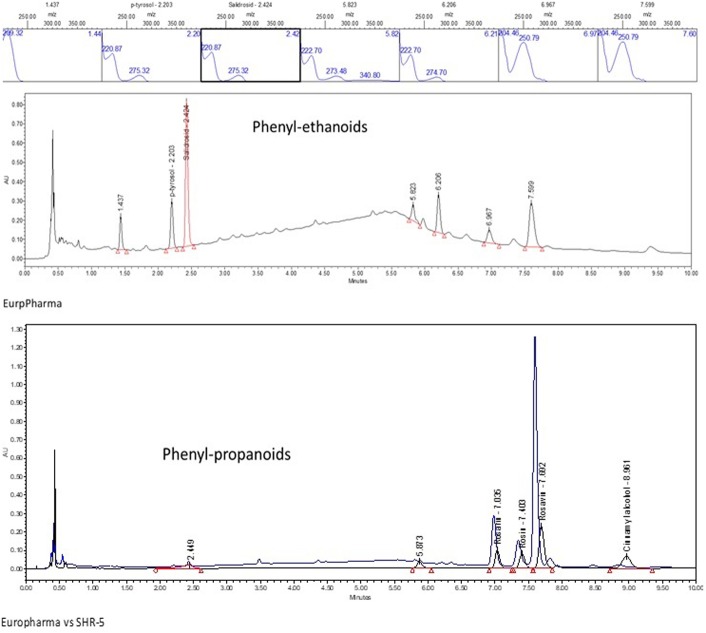
UPLC-UV fingerprint of the *R. rosea* rhizome extracts EPR-7 and SHR-5: lower panel – overlay of chromatograms of EPR-7 and SHR-5 detected at 252 nm, middle panel – EPR-7 detected at 221 nm, upper panel – UV spectra of tyrosol, salidroside, rosarin, rosin, rosavin, and cinnamyl alcohol.

**FIGURE 3:**
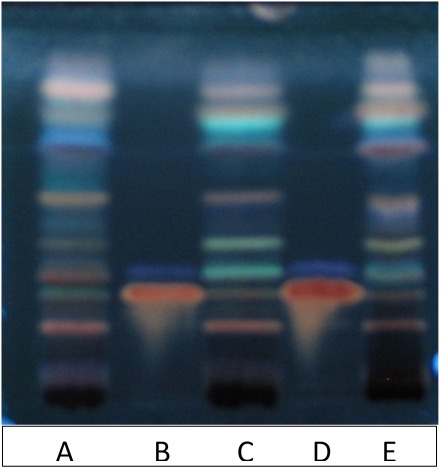
High performance TLC (HPTLC) fingerprint of extracts exposed to UV 366 nm, after derivatization with 10% sulfuric acid in methanol. Track A – EPR-7, track C – SHR-5, track E – Alt-S, track B and D – reference standards rosarin *R*_f_ = 0.28 and salidroside *R*_f_ = 0.36. Solvent system: EtOAc:MeOH:H_2_O:HCOOH, 77:13:10:2.

Representative UPLC and HPTLC fingerprints of two *R. rosea* extracts, EPR-7 and the reference standard SHR-5, are shown in **Figures 2**, **3**. They are almost identical except for the peaks corresponding to rosavins, which are higher on the chromatogram of EPR-7 (**Figure 2**) and the presence of some fluorescent compounds in SHR-5, which are absent in EPR-7 (**Figure 3**).

The contents of all active markers in all studied extracts (**Table 1**) were quite different, with a lack of rosavin, rosin, and rosarin in one extract from China. Even the extracts from the same geographic region – Altai (Russia) differed; e.g., the content of rosavin in Alt-X was 100-fold lower (0.03%) compared with SHR-5 (3.1%) or EPR-7 (3.7%), despite that all other phenyl- and ethyl propanoids were within common limits. This may have a significant impact on the activity of the *Rhodiola* Alt-X extract in T cell activation and apoptosis (Marchev et al., 2017). Overall, the highest content of rosavin and salidroside was in the *Rhodiola* EPR-7 extract.

### Electrophysiological Activity in a Synaptic Model of Memory: Hippocampal Long-Term Potentiation

**FIGURE 4:**
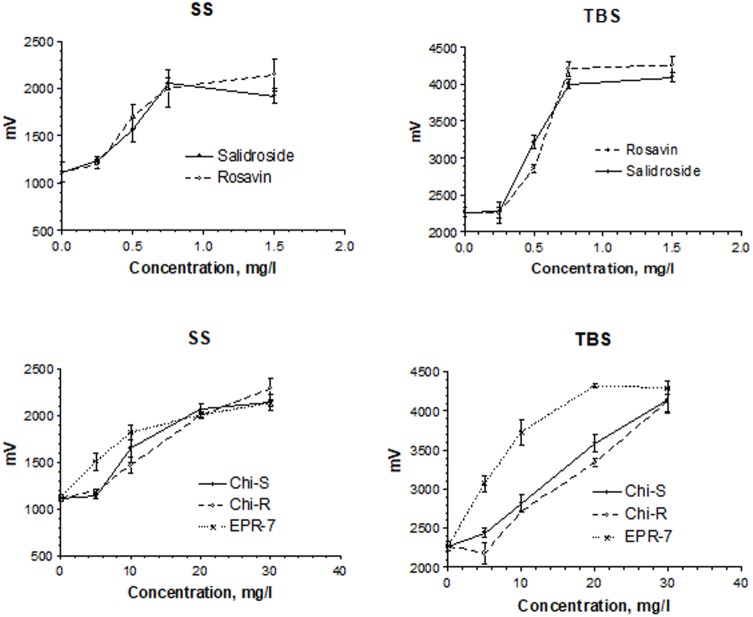
Concentration-dependent effects of salidroside and rosavin on pyramidal cell activity in terms of changes of population spike amplitudes (millivolts). Results were obtained after a single stimuli (SS) or burst stimuli (TBS). Data represent the mean ± SEM of *n* = 4 slices (all concentrations). The details of statistical analysis see in Supplementary Data S3.

**Figure 4** shows the concentration-dependent effects of salidroside and rosavin on pyramidal cell activity in terms of changes of population spike amplitudes (millivolts) in hippocampus slices. In the presence of salidroside, the amplitudes of the population spike were enhanced in a concentration-dependent manner. During SS, amplitudes reached about 2.3 mV and about 4.3 mV during TBS. At 0.5 mg/L, salidroside was more effective than rosavin in the TBS test, while at the higher concentrations of 0.75 and 1.5 mg/L, the effect of rosavin was superior (**Figure 4**, upper part).

**FIGURE 5:**
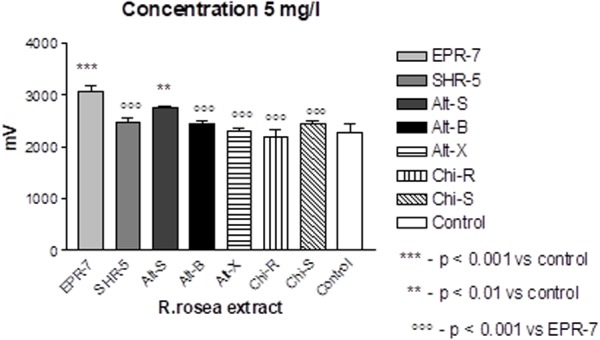
Effects of *Rhodiola* extracts at the lowest active concentration (5 mg/L) on pyramidal cell activity in terms of changes in population spike amplitudes (millivoltage). Results are obtained after burst stimuli. Data represent the mean ± SEM of *n* = 4 slices (all concentrations). The details of statistical analysis see in Supplementary Data S3.

A comparison of the *Rhodiola* extract Chi-R, containing the phenyl ethanoids, tyrosol and salidroside, with the extracts containing both salidroside and rosavin, e.g., Chi-R vs. Rhodiola EPR-7, demonstrated that the content of salidroside in both extracts was almost the same 2.5–3.0%, while the content of rosavin in EPR-7 was 3.5-fold higher than in Chi-R (**Table 1**). That is in line with results where the EPR-7 extract was as active as Chi-R at a 2.5-fold lower concentration (**Figure 4**, lower part, and **Figure 5**). **Figure 5** shows that EPR-7 was the most active extract at 5 mg/L, which corresponds to a concentration of rosavin of 0.18 mg/L (0.4 μM).

**FIGURE 6:**
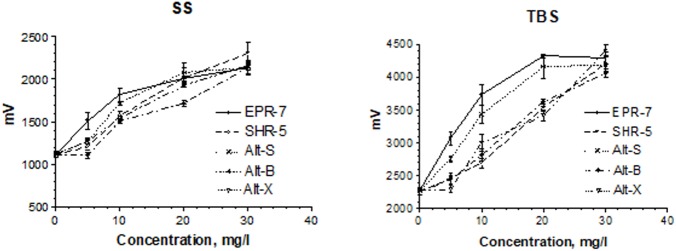
Concentration-dependent effects of *Rhodiola* extracts on pyramidal cell activity in terms of changes in the population spike amplitudes (millivolts). Results were obtained after a single stimuli (SS) or burst stimuli (TBS). Data represent the mean ± SEM of *n* = 4 slices (all concentrations). The details of statistical analysis see in Supplementary Data S3.

The EC_50_ values (**Table 2**) were calculated during SS and TBS. All results were fitted using a hyperbolic tangent function to give EC_50_ values (effective concentration to induce a half-maximal effect). The lower the EC_50_ values, the less compound is needed to exert its pharmacological effect. The lower the effective concentration, the less side effects are expected. According to this bioassay, the EPR-7 extract had the lowest EC_50_ value among the seven *Rhodiola* extracts compared in this study (**Table 2** and **Figures 5**, **6**).

## Discussion

In this study, we compared the biological activity of various commercial extracts of *R. rosea* in a synaptic model of memory: the LTP of synaptic transmission in the hippocampus (Bliss and Collingridge, 1993). Synaptic transmission is critical in learning, memory, and functioning of the nervous system. As memories are thought to be determined by alteration of synaptic strength, a persistent increase in synaptic strength following high-frequency stimulation of a chemical synapse (LTP) is generally considered one of the major cellular mechanisms that triggers learning and memory.

When comparing the content of rosavin and/or salidroside in seven extracts with their EC_50_ values (**Table 2**), one can conclude that the EPR-7 extract, which has the highest content of both active markers, was active at the lowest concentrations. However, this correlation did not apply to other extracts, e.g., SHR-5, which presumably contained compounds that reduced the overall efficacy of the total extracts (**Figure 3**). Active compounds other than rosavin or salidroside might account for the efficacy of the extracts in this bioassay through synergistic or antagonistic modes of action. In this study, the brain slice is exposed directly to the samples, circumventing the blood–brain barrier. However, both preclinical and clinical studies have proven that *Rhodiola* extract exerts actions on the brain, suggesting enough compound passes the blood–brain barrier.

The efficacy of *Rhodiola* SHR-5 extract was demonstrated previously on healthy subjects (Spasov et al., 2000; Shevtsov et al., 2003; Dimpfel, 2014), subjects experiencing stress and fatigue (Darbinyan et al., 2000), and patients with chronic fatigue (Olsson et al., 2009) and major depressive disorder (Darbinyan et al., 2007; Mao et al., 2015). In a double blind, placebo-controlled study on 20 healthy subjects, Dimpfel demonstrated that a single dose administration of two capsules containing 200 mg *R. rosea* SHR-5 extract changed the spectral signature of electric brain activity in a stimulating way compared with placebo. The effect was regarded as a safe booster of mental activity during cognitive and emotional challenges. *Rhodiola* EPR-7 extract was also studied earlier in isolated skeletal muscle cells (Hernández-Santana et al., 2014), animals (Dimpfel et al., 2016b), and healthy human subjects (Shanely et al., 2014; Ahmed et al., 2015). Oral administration of 100 mg/kg of *R. rosea* root extract led to significant attenuation of α1, α2, β1, β2, δ, and θ waves of the electropharmacograms, which are associated with the activation of dopamine, serotonin, glutamate, GABA, acetylcholine, and norepinephrine-mediated signaling pathways (Dimpfel et al., 2016b). The most affected were α2 (dopaminergic transmission – CNS stimulating effect) and β1 (glutaminergic transmission – CNS stimulating effect) in the frontal cortex. The next strongest changes were seen in the striatum, and the weakest changes in the reticular formation. Spectral changes lasted up to 4 h after administration.

These results are in line with those of our recent publication, where we evaluated the effects of *Rhodiola* extract and salidroside on gene expression profiling in the T98G human neuroglia cell line (Panossian et al., 2014). The most significantly affected canonical pathways across the entire dataset, which contains the 1062 genes deregulated by *Rhodiola* and salidroside, were G-protein coupled receptor signaling, glutamate receptor signaling, ephrin receptor signaling, cAMP-mediated pathways, and dopamine signaling pathways associated with the expression of cell survival genes (Panossian et al., 2014). A meta-analysis on the putative antidepressant action of *Rhodiola* extract revealed it was effective on major depressive disorder (146 subjects) and stress-induced mild depression (714 individuals) (Amsterdam and Panossian, 2016). Rosavin was not included in that study. The results of our study are in line with a previous publication where the LTP effect of Rhodiola extract was tested *in vitro* in the hippocampal slice (Dimpfel et al., 2016b). A concentration of 5 mg/L induced a slight increase in the amplitude of the population spike and an increase in LTP. Further increases were observed by increasing the concentration up to 30 mg/L. During TBS, amplitudes of more than 4 mV were measured, indicating their effect on LTP.

## Conclusion

In conclusion, rosavin, salidroside and various *R. rosea* extracts potentiated the *in vitro* electric stimulation of an intra-hippocampal electric circuit, which resulted in higher responses of pyramidal cells in isolated hippocampus slices. Rosavin was more active in higher concentrations than salidroside; while, salidroside was more effective at lower concentrations. The highest content of both active markers was found in the extracts that were active at the lowest concentrations. Although, this correlation was not applicable to some extracts containing other compounds that presumably reduced the efficacy due to antagonistic interactions. The standardized content of active markers is necessary for the quality control of herbal preparations containing *Rhodiola* extracts, but insufficient for assessment of their potential efficacy. The application of bioassays should be required for adequate assessment of the quality and efficacy of *R. rosea* extracts.

## Availability of Data and Material

The datasets used and/or analyzed during the current study are available from the corresponding author upon reasonable request.

## Ethics Statement

The principles of laboratory animal care were followed in all trials and the local authority (“Regierungspräsidium” Giessen) – responsible for animal care – was informed according to German Health Guidelines. Details of the acclimatization, housing conditions, and surgery have been reported (Dimpfel et al., 1991). Experiments were performed according to §4 German Animal Protection Law (Tierschutzgesetz), which states, that animals are allowed to be killed for taking out organs for scientific purposes. Allowance to keep animals is renewed by governmental authority every 3 years.

## Author Contributions

AP planned the experiments and wrote the manuscript. LS performed the experiments. WD carried out data analysis and wrote a report on the results. All authors critically revised and approved the final version of the manuscript.

## Conflict of Interest Statement

AP is currently a consultant to EuroPharma USA Inc., Founder of Phytomed AB (Sweden), and former Head of Research Development at the Swedish Herbal Institute, Gothenburg, Sweden. He is not a member of any pharmaceutical industry-sponsored advisory board or speaker’s bureau and has no significant financial interest in any pharmaceutical company. WD and LS are not members of any pharmaceutical industry-sponsored advisory board or speaker’s bureau, and have no significant financial interest in any pharmaceutical company. The samples of Rhodiola extracts used in the study were donated by the manufacturer EuroPharma USA Inc. EuroPharma USA Inc. had no other involvement in the study.
