# Supplementary material for: Assessing the Quality and Potential Efficacy of Commercial Extracts of Rhodiola rosea L. by Analyzing the Salidroside and Rosavin Content and the Electrophysiological Activity in Hippocampal Long-Term Potentiation, a Synaptic Model of Memory
Source: Front Pharmacol. 2018 May 24;9:425. doi: 10.3389/fphar.2018.00425 (PMC5976749; doi:10.3389/fphar.2018.00425)
Supplement: Supplementary file 1 [file Data_Sheet_1.docx]

**Supplementary data S1. Table 1. Test samples codes,** the country of origin of herbal substance, the batch number, year and month of harvest, extraction solvent, evaporation temperature, DERnative, declared content of markers

| **No** | **Sample code** | **Country of origin of herbal**  **substance** | **Batch #** | **Harvest**  **Month**  **Year** | **Extraction solvent** | **Evaporation temperature** | **DER_native_** | **Declared**  **Content of**  **markers** | **Found**  **Content of**  **markers** |
| --- | --- | --- | --- | --- | --- | --- | --- | --- | --- |
| 1 | SHR-5 | Altai, Russia | 1421325 | July august  2015 | 70% ehthanol | +60 ^o^ C | 5:1 | 3,1% rosavin, 2.1% salidroside  4.8% rosavins | 3,1% rosavin,  2.1% salidroside  4.8% rosavins |
| 2 | EPR-7 | Altai, Russia | RRE05F17-0901 | Not specified | 70% ehthanol | -70 ^o^ C | 7-10:1 | 5% rosavin, 3% salidroside | 3,7% rosavin,  3.1% salidroside  **5.1% rosavins** |
| 3 | Chi-S | China | SQT161220 | Not specified | Not specified | Not specified | Not specified | 1 % salidroside, | 1,1 % salidroside, no rosavins |
| 4 | Chi-R | China | SQT170110 | Not specified | Not specified | Not specified | Not specified | 3% rosavins,  1% salidroside | 1.2% rosavin,  2.5% salidroside  2.1% rosavins |
| 5 | Alt-B | Altai, Russia | 08072016-01 | July august  2016 | water | +60 ^o^ C | 7:1 | Not specified | 1.1% rosavin,  0.9% salidroside  2.1% rosavins |
| 6 | Alt-S | Altai, Russia | 000103 | July-August 2015 | 70% ehthanol | -70 ^o^ C | 5:1 | 7.2% rosavins, 2.1% salidroside | 1.3% rosavin,  1.9% salidroside  3.2% rosavins |
| 7 | Alt-X | Altai, Russia | 2502-2016 | August-September  2016 | 35% ehthanol | +50 ^o^ C | 6:1 | 2 % salidroside | 0,3% rosavin,  1.9% salidroside  1.5% rosavins |
| 8 | Rosavin | Sigma Aldrich  PhytoLab GmbH |  |  |  |  |  |  |  |
| 9 | Salidroside | Sigma Aldrich  PhytoLab GmbH |  |  |  |  |  |  |  |

| **Parameter** | **Acceptance criteria** | | **Results** | |
| --- | --- | --- | --- | --- |
|  |  | | **Salidroside** | **Rosavin** |
| Method linearity | Correlation coefficient R≥0,999 | | 0,999871 | 0,999986 |
| Method precision | Repeatability | RSD< 5 % | Level 50%=0,22%  Level 100%=0,45 %  Level 150%=0,21 % | Level 50%=0,14 %  Level 100%=0,17 %  Level 150%=0,16 % |
|  | Intermediate precision (diff. days) | RSD< 5 % | 2,36% | 2,10% |
|  | Intermediate precision (diff. operator) | RSD< 5 % | 1,17% | 0,74% |
| Method accuracy | Recovery: 90-110 % | | 101,41 % | 101,20% |
| Selectivity | Purity: PA less than PT  Resolution > 2 | | PA=2,775  PT=3,130  Resolution=4,28 | PA=0,289  PT=1,288  Resolution=2,93 |
| Range | Min 80-120% | | 50-150% | 50-150% |
| Robustness | Different columns  %RSD ≤ 2 % | | 0,46 % | 1,87% |
|  | Stability of the solutions RSD ≤ 2 % | | 0,61 % (same day) | 0,17 % (same day) |
|  | Extraction time: 25 min RSD ≤ 2 % | | 0,45 % | 0,19 % |
|  | Extraction time: 35 min RSD ≤ 2 % | | 0,70 % | 0,42 % |

**Table 2.** UPLC method validation results.
