# Supplementary material for: Assessing the Quality and Potential Efficacy of Commercial Extracts of Rhodiola rosea L. by Analyzing the Salidroside and Rosavin Content and the Electrophysiological Activity in Hippocampal Long-Term Potentiation, a Synaptic Model of Memory
Source: Front Pharmacol. 2018 May 24;9:425. doi: 10.3389/fphar.2018.00425 (PMC5976749; doi:10.3389/fphar.2018.00425)
Supplement: Supplementary file 2 [file Data_Sheet_2.pdf]

Empower2 Software

Sample: RR 1 Alt-x Rapport_extrakt_sicc

Method: SM-127		Acquired By:	Alexander
Sample Type:	Unknown	Date Acquired:	2017-05-23 10:33:54 CEST,
Vial:	1:C,1	Acq. Method Set:	Rhodiola 6 markers
Injection #:	1, 2, 3	Date Processed:	2017-05-23 14:51:59 CEST,
Injection Volume:	2.00 ul	Processing Method	Rhodiola_221, Rhodiola_252
Run Time:	10.0 Minutes	Channel Name:	221 nm, 252 nm
Sample Set Name	RR T 0105 UOC	Column no.: 0216331121573ε	

Component Results
Name: p-tyrosol

	SampleName	Injection Volume (uL)	SampleWeight	RT	Area	Height	Name	Percent_amount
1	RR 1	2.00	15.02000	2.226	529583	20112C	p-tyrosol	0.3841
2	RR 1	2.00	15.02000	2.227	531221	200918	p-tyrosol	0.3853
3	RR 1	2.00	15.02000	2.232	527236	201559	p-tyrosol	0.3824
Mean								0.3839
Std. Dev.								0.0015
% RSD								0.38

Component Results
Name: Salidrosid

	SampleName	Injection Volume (uL)	SampleWeight	RT	Area	Height	Name	Percent_amount
1	RR 1	2.00	15.02000	2.453	111545(	406329	Salidrosid	1.8513
2	RR 1	2.00	15.02000	2.453	111479:	405886	Salidrosid	1.8502
3	RR 1	2.00	15.02000	2.459	111629‹	409567	Salidrosid	1.8527
Mean								1.8514
Std. Dev.								0.0012
% RSD								0.07

Component Results
Name: Rosarin

	SampleName	Injection Volume (uL)	SampleWeight	RT	Area	Height	Name	Percent_amount
1	RR 1	2.00	15.02000	7.016	1264412	274936	Rosarin	1.0041
2	RR 1	2.00	15.02000	7.021	1265292	275499	Rosarin	1.0048
3	RR 1	2.00	15.02000	7.028	1266709	276896	Rosarin	1.0059
Mean								1.0049
Std. Dev.								0.0009
% RSD								0.09

Component Results
Name: Rosin

	SampleName	Injection Volume (uL)	SampleWeight	RT	Area	Height	Name	Percent_amount
1	RR 1	2.00	15.02000	7.383	878634	162934	Rosin	0.5050
2	RR 1	2.00	15.02000	7.389	879802	163342	Rosin	0.5057
3	RR 1	2.00	15.02000	7.397	881148	163945	Rosin	0.5064
Mean								0.5057
Std. Dev.								0.0007
% RSD								0.14

Sign.: [signature]

Component Results
Name: Rosav in

	SampleName	Injection Volume (uL)	SampleWeight	RT	Area	Height	Name	Percent_amount
1	RR 1	2.00	15.02000	7.673	46777	8548	Rosavin	0.0282
2	RR 1	2.00	15.02000	7.679	48360	8648	Rosavin	0.0295
3	RR 1	2.00	15.02000	7.688	42449	8245	Rosavin	0.0248
Mean								0.0275
Std. Dev.								0.0024
% RSD								8.80

Component Results
Name: Cinnamyl alcohol

	SampleName	Injection Volume (uL)	SampleWeight	RT	Area	Height	Name	Percent_amount
1	RR 1	2.00	15.02000	8.500			Cinnamyl alcohol	
2	RR 1	2.00	15.02000	8.930	3864972	420802	Cinnamyl alcohol	1.0646
3	RR 1	2.00	15.02000	8.943	3868105	421280	Cinnamyl alcohol	1.0654
Mean								1.0650
Std. Dev.								0.0006
% RSD								0.06

Result   ***%***   ***Calculated***   ***Recovery***

p-tyrosol:

Salidrosid

Rosarin:

Rosin:

Rosavin:

Cinnamylalcohol:

Sign.

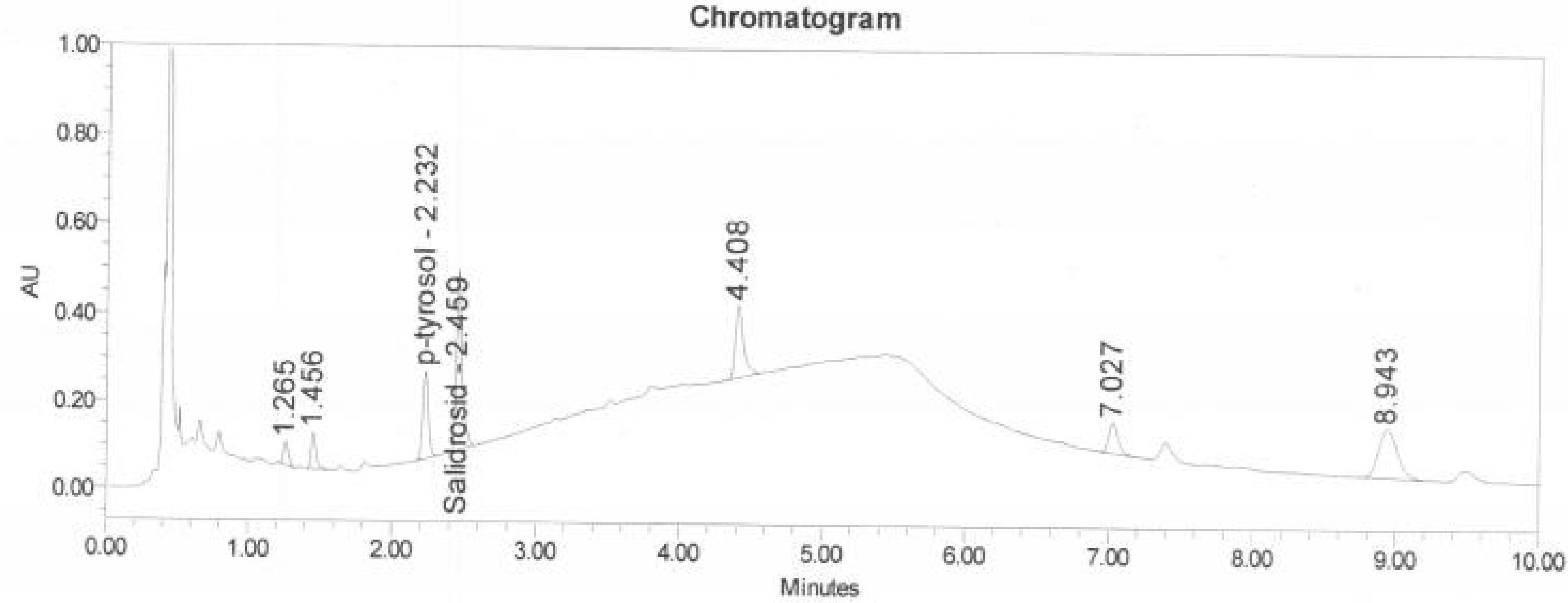

SampleName RR 1; Vial 1:C,1; Date Acquired 2017-05-23 10:33:54 CEST; Channel Name 221 nm

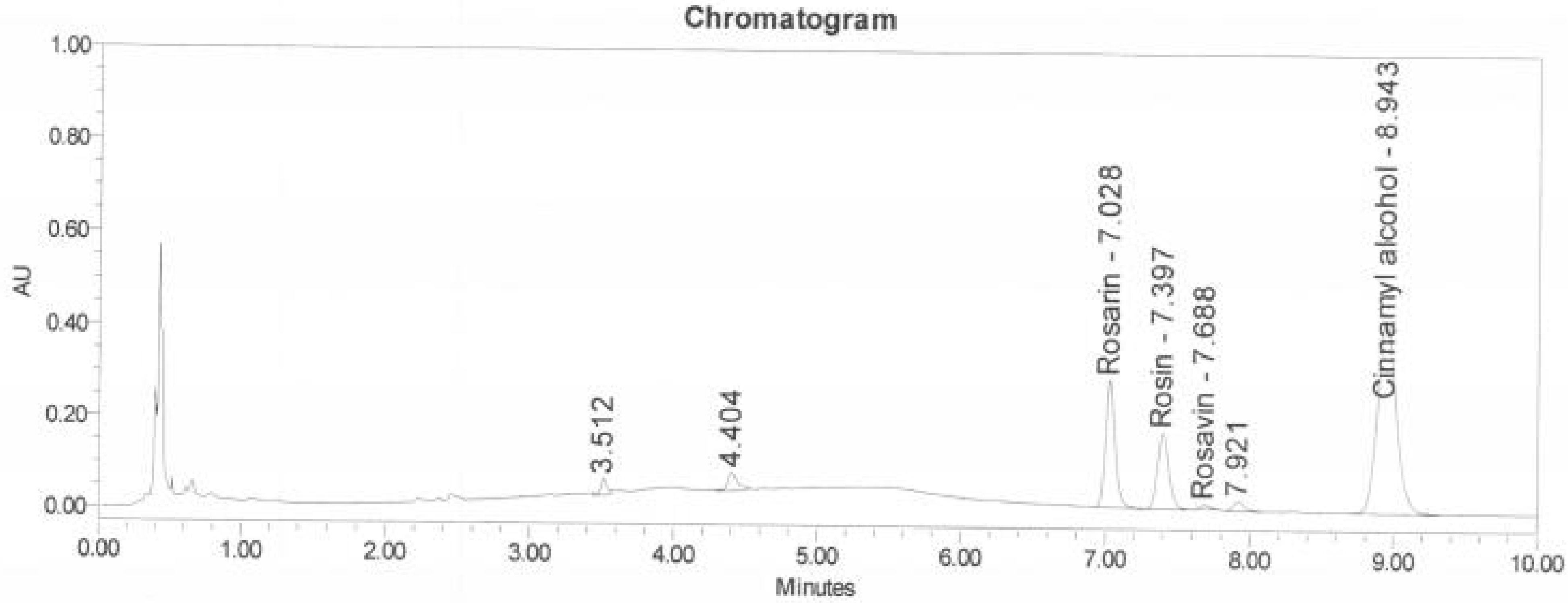

SampleName RR 1; Vial 1:C,1; Date Acquired 2017-05-23 10:33:54 CEST; Channel Name 252 nm

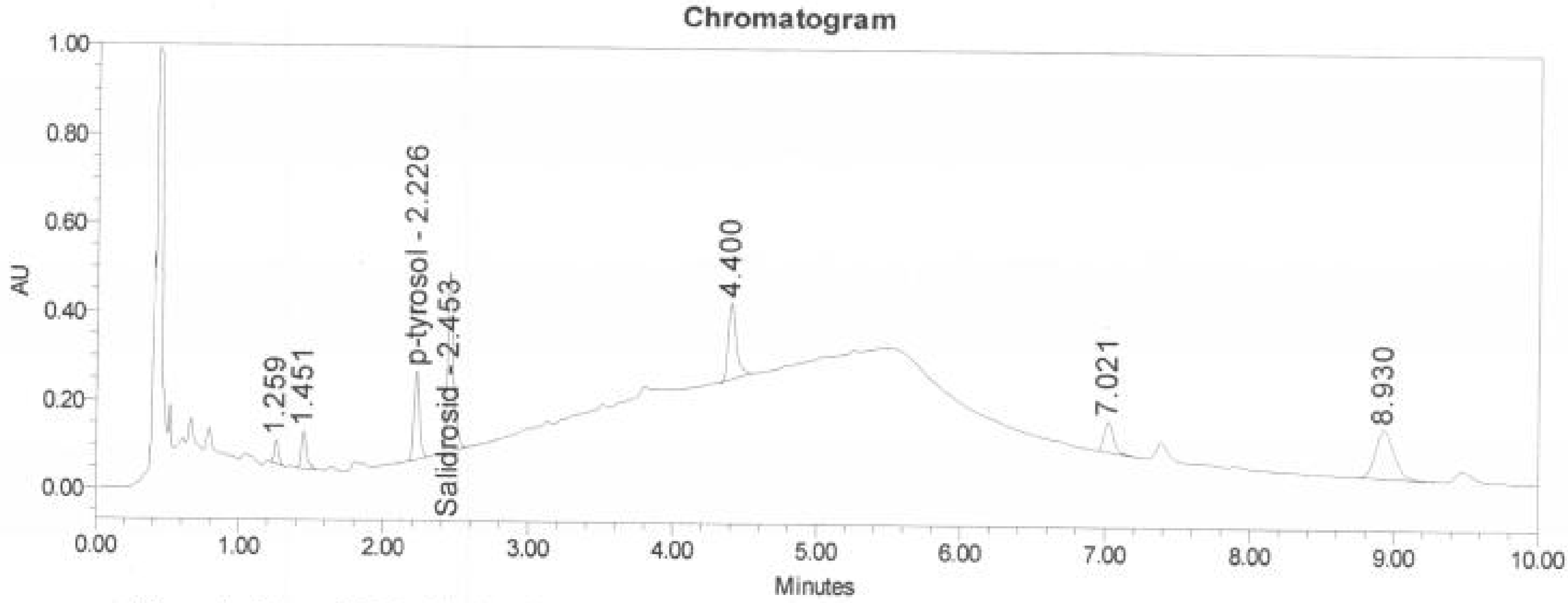

SampleName RR 1; Vial 1:C,1; Date Acquired 2017-05-23 11:13:56 CEST; Channel Name 221 nm

Sign.:

Chromatogram

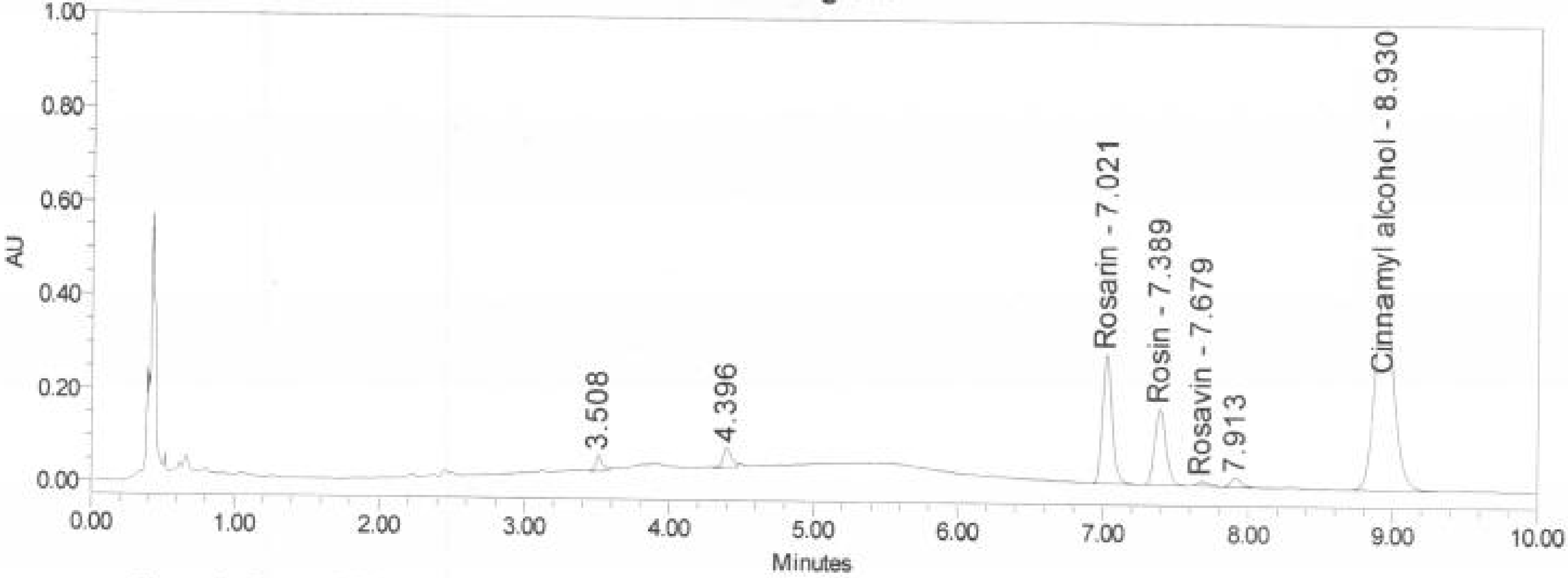

SampleName RR 1; Vial 1:C,1; Date Acquired 2017-05-23 11:13:56 CEST; Channel Name 252 nm

Chromatogram

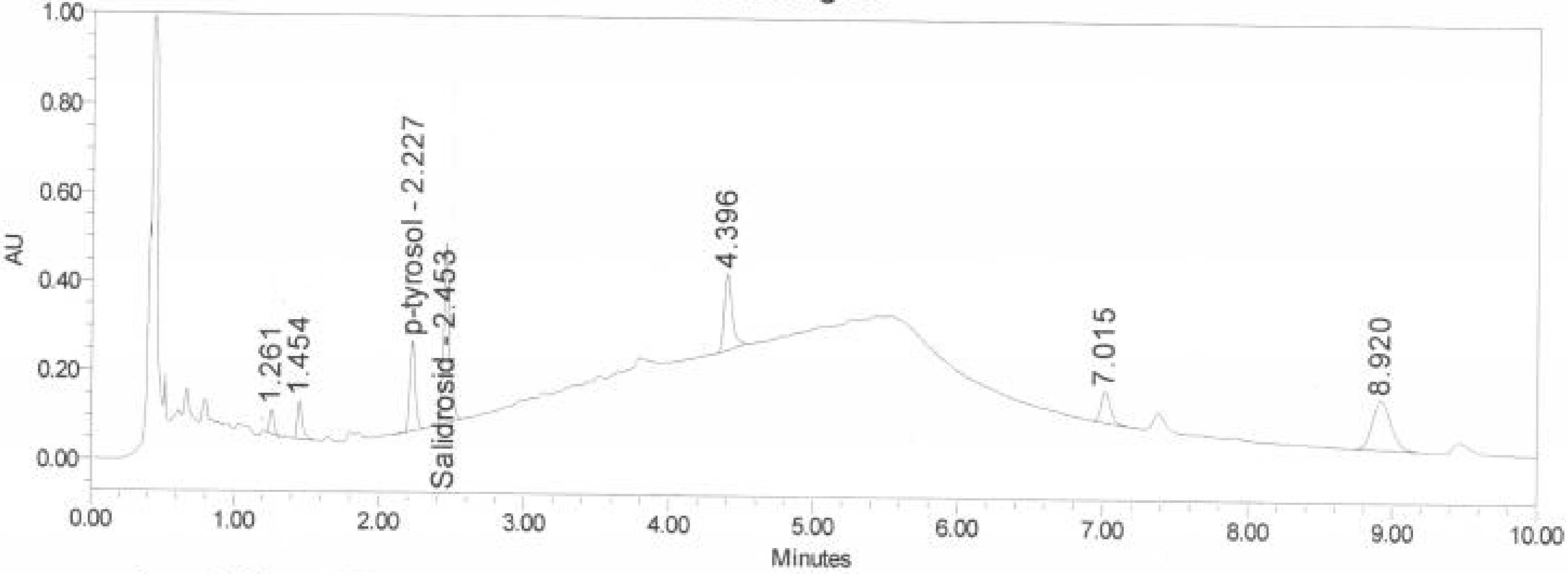

SampleName RR 1; Vial 1:C,1; Date Acquired 2017-05-23 11:54:01 CEST; Channel Name 221 nm

Chromatogram

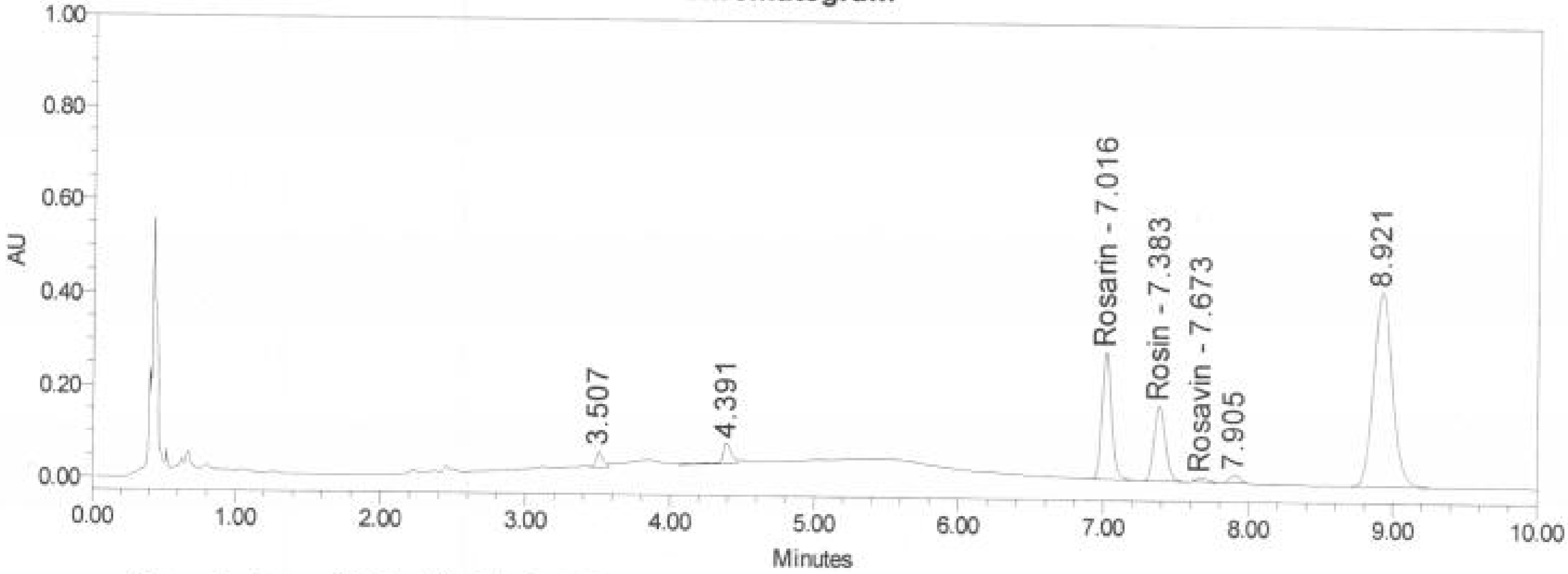

SampleName RR 1; Vial 1:C,1; Date Acquired 2017-05-23 11:54:01 CEST; Channel Name 252 nm

Sign.:

Empower2 software

Sample: RR 2 Alt-S Rapport_extrakt_sicc

Method: SM-127
Sample Type: Unknown
Vial: 1:C,2
Injection #: 1, 2, 3
Injection Volume: 2.00 ul
Run Time: 10.0 Minutes
Sample Set Name RR T 0105 UOC

Acquired By: Alexander
Date Acquired: 2017-05-23 12:34:03 CEST,
Acq. Method Set: Rhodiola 6 markers
Date Processed: 2017-05-23 15:03:41 CEST,
Processing Method Rhodiola_221, Rhodiola_252
Channel Name: 221 nm, 252 nm
Column no.: 021633112157З8

Component Results
Name: p-tyrosol

	SampleName	Injection Volume (uL)	SampleWeight	RT	Area	Height	Name	Percent_amount
1	RR 2	2.00	16.29000	2.217	356302	136108	p-tyrosol	0.2383
2	RR 2	2.00	16.29000	2.220	357679	136794	p-tyrosol	0.2392
3	RR 2	2.00	16.29000	2.221	356654	136375	p-tyrosol	0.2385
Mean								0.2387
Std. Dev.								0.0005
% RSD								0.20

Component Results
Name: Salidrosid

	SampleName	Injection Volume (uL)	SampleWeight	RT	Area	Height	Name	Percent_amount
1	RR 2	2.00	16.29000	2.441	1251578	490354	Salidrosid	1.9135
2	RR 2	2.00	16.29000	2.445	1253171	493788	Salidrosid	1.9159
3	RR 2	2.00	16.29000	2.446	1253586	492672	Salidrosid	1.9166
Mean								1.9153
Std. Dev.								0.0016
% RSD								0.08

Component Results
Name: Rosarin

	SampleName	Injection Volume (uL)	SampleWeight	RT	Area	Height	Name	Percent_amount
1	RR 2	2.00	16.29000	6.992	1580714	343253	Rosarin	1.1584
2	RR 2	2.00	16.29000	6.995	1583368	344149	Rosarin	1.1604
3	RR 2	2.00	16.29000	7.000	1583759	344997	Rosarin	1.1607
Mean								1.1598
Std. Dev.								0.0012
% RSD								0.11

Component Results
Name: Rosin

	SampleName	Injection Volume (uL)	SampleWeight	RT	Area	Height	Name	Percent_amount
1	RR 2	2.00	16.29000	7.357	1320204	245833	Rosin	0.7004
2	RR 2	2.00	16.29000	7.361	1322014	246494	Rosin	0.7014
3	RR 2	2.00	16.29000	7.366	1322373	246572	Rosin	0.7016
Mean								0.7011
Std. Dev.								0.0006
% RSD								0.09

Sign.: [signature]

Component Results
Name: Rosavin

	SampleName	Injection Volume (uL)	SampleWeight	RT	Area	Height	Name	Percent_amount
1	RR 2	2.00	16.29000	7.636	1836037	352947	Rosavin	1.3293
2	RR 2	2.00	16.29000	7.639	1839620	353800	Rosavin	1.3319
3	RR 2	2.00	16.29000	7.645	1841258	355043	Rosavin	1.3331
Mean								1.3315
Std. Dev.								0.0019
% RSD								0.15

Component Results
Name: Cinnamyl alcohol

	SampleName	Injection Volume (uL)	SampleWeight	RT	Area	Height	Name	Percent_amount
1	RR 2	2.00	16.29000	8.883	3320271	364184	Cinnamyl alcohol	0.8431
2	RR 2	2.00	16.29000	8.890	3325718	364808	Cinnamyl alcohol	0.8444
3	RR 2	2.00	16.29000	8.899	3327393	364120	Cinnamyl alcohol	0.8449
Mean								0.8441
Std. Dev.								0.0009
% RSD								0.11

Result ***%*** ***Calculated*** ***Recovery***

p-tyrosol:

Salidrosid

Rosarin:

Rosin:

Rosavin:

Cinnamylalcohol:

Sign.:

Chromatogram

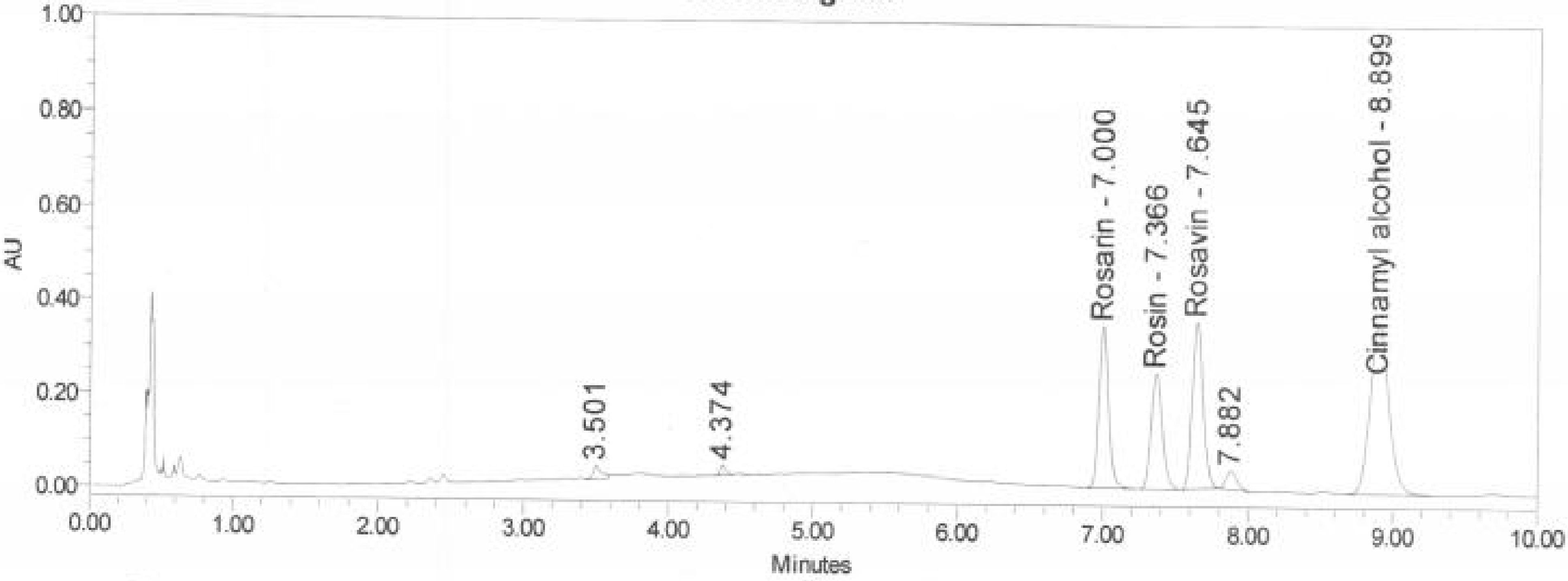

SampleName RR 2; Vial 1:C,2; Date Acquired 2017-05-23 12:34:03 CEST; Channel Name 252 nm

Chromatogram

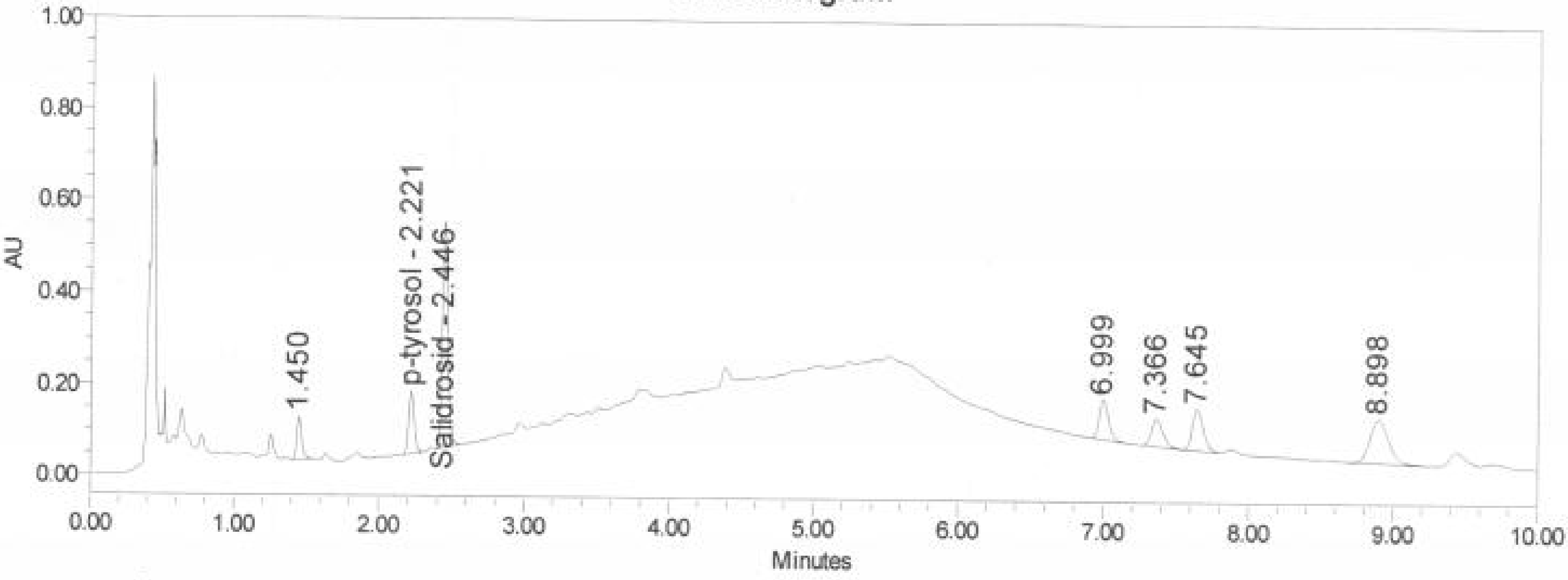

SampleName RR 2; Vial 1:C,2; Date Acquired 2017-05-23 12:34:03 CEST; Channel Name 221 nm

Chromatogram

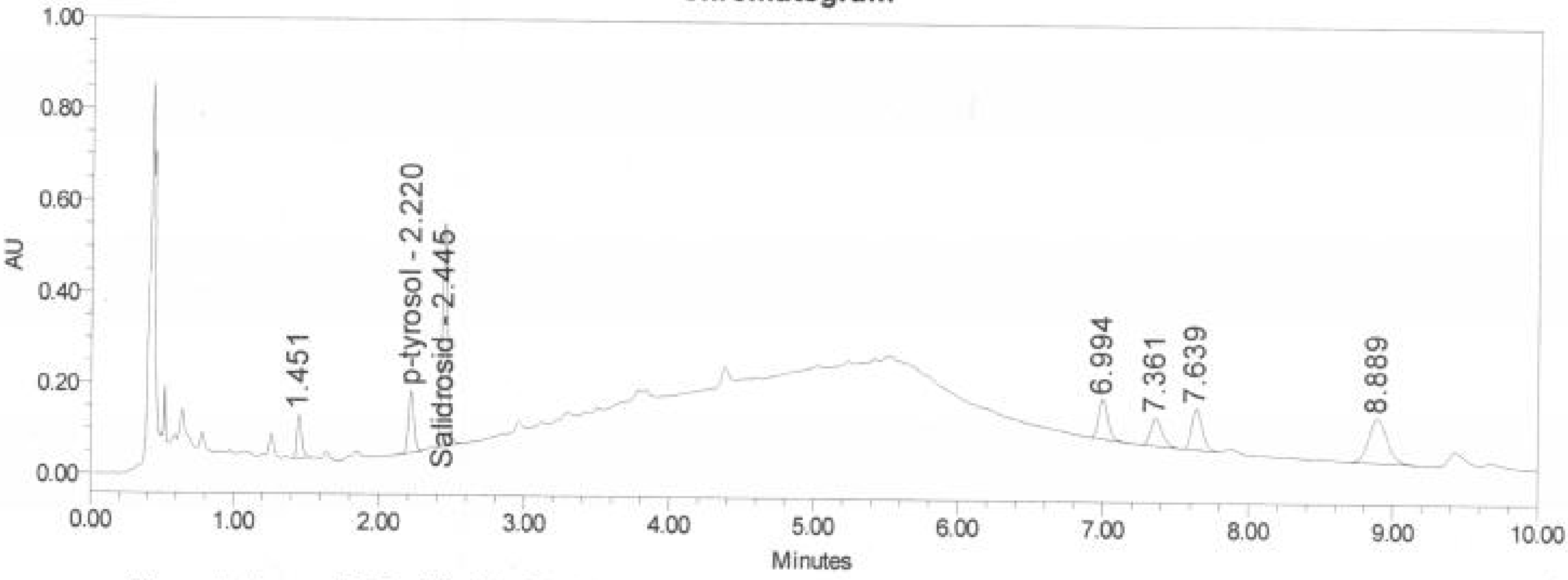

SampleName RR 2; Vial 1:C,2; Date Acquired 2017-05-23 13:14:06 CEST; Channel Name 221 nm

Sign.:

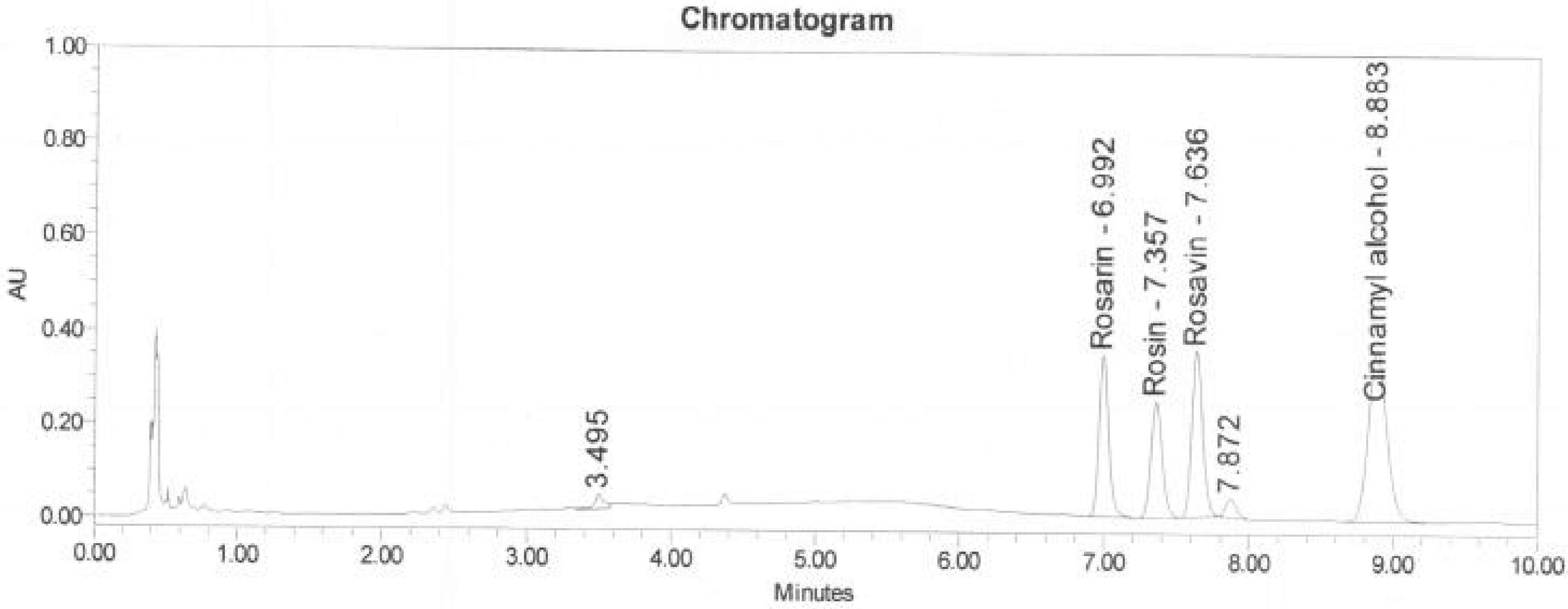

SampleName RR 2; Vial 1:C,2; Date Acquired 2017-05-23 13:54:06 CEST; Channel Name 252 nm

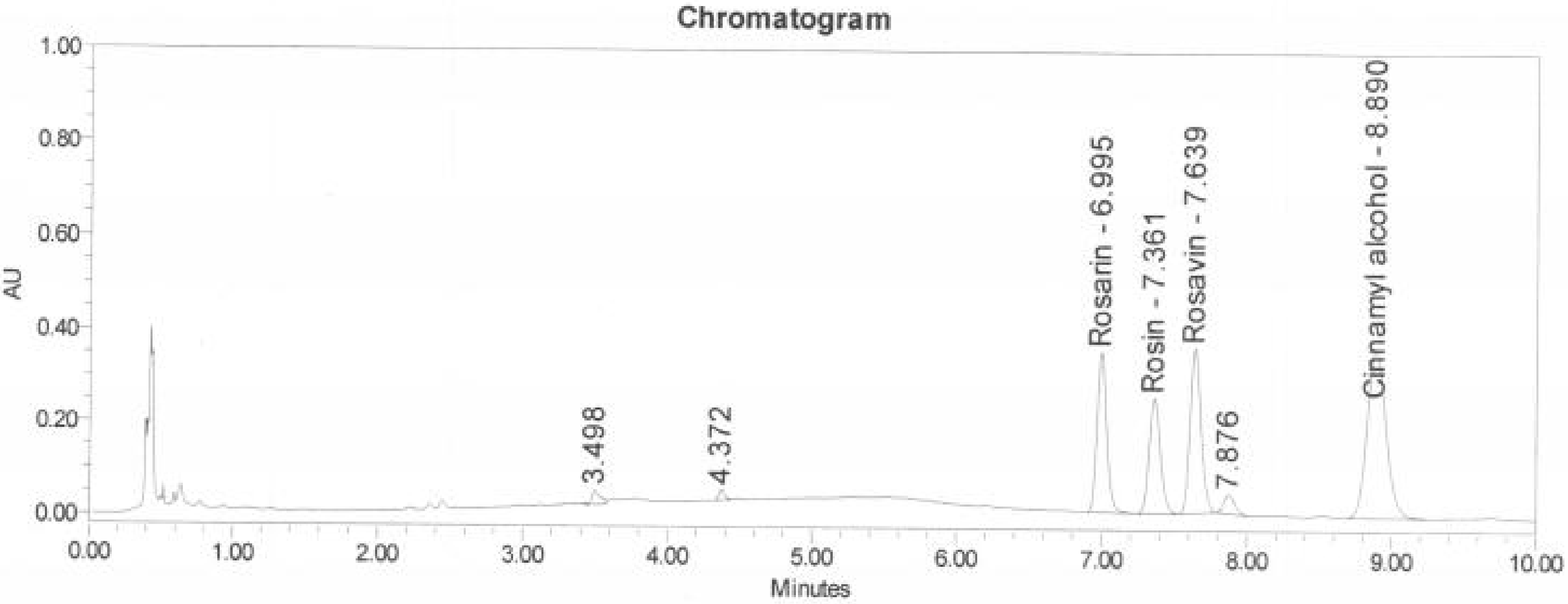

SampleName RR 2; Vial 1:C,2; Date Acquired 2017-05-23 13:14:06 CEST; Channel Name 252 nm

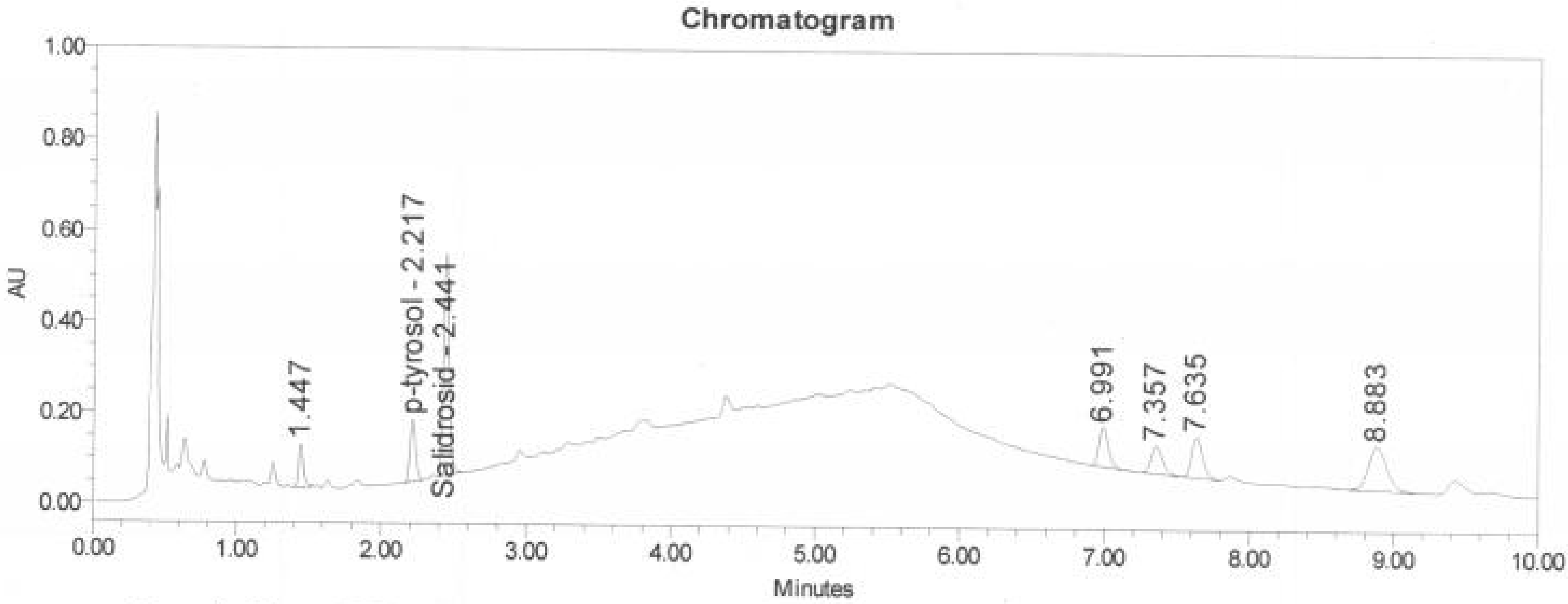

SampleName RR 2; Vial 1:C,2; Date Acquired 2017-05-23 13:54:06 CEST; Channel Name 221 nm

Sign.:

Empower2 software

Sample: RR 3 Alt-B Rapport_extrakt_sicc

Method: SM-127		Acquired By:	Alexander
Sample Type:	Unknown	Date Acquired:	2017-05-23 14:34:13 CEST,
Vial:	1:C,3	Acq. Method Set:	Rhodiola 6 markers
Injection #:	1, 2, 3	Date Processed:	2017-05-23 15:23:48 CEST,
Injection Volume:	2.00 ul	Processing Method	Rhodiola_221, Rhodiola_252
Run Time:	10.0 Minutes	Channel Name:	221 nm, 252 nm
Sample Set Name	RR T 0105 UOC	Column no.:	0216331121573[illegible]

Component Results
Name: p-tyrosol

	SampleName	Injection Volume (uL)	SampleWeight	RT	Area	Height	Name	Percent_amount
1	RR 3	2.00	12.24000	2.204	385691	149795	p-tyrosol	0.3433
2	RR 3	2.00	12.24000	2.206	385439	150266	p-tyrosol	0.3431
3	RR 3	2.00	12.24000	2.220	384903	150187	p-tyrosol	0.3426
Mean								0.3430
Std. Dev.								0.0004
% RSD								0.10

Component Results
Name: Salidrosid

	SampleName	Injection Volume (uL)	SampleWeight	RT	Area	Height	Name	Percent_amount
1	RR 3	2.00	12.24000	2.431	440580	173711	Salidrosid	0.9088
2	RR 3	2.00	12.24000	2.433	440339	174645	Salidrosid	0.9083
3	RR 3	2.00	12.24000	2.449	440203	175518	Salidrosid	0.9081
Mean								0.9084
Std. Dev.								0.0004
% RSD								0.04

Component Results
Name: Rosarin

	SampleName	Injection Volume (uL)	SampleWeight	RT	Area	Height	Name	Percent_amount
1	RR 3	2.00	12.24000	6.966	735656	163708	Rosarin	
2	RR 3	2.00	12.24000	6.971	735942	164577	Rosarin	0.7147
3	RR 3	2.00	12.24000	6.992	735855	164400	Rosarin	0.7147
Mean								0.7147
Std. Dev.								0.0001
% RSD								0.01

Component Results
Name: Rosin

	SampleName	Injection Volume (uL)	SampleWeight	RT	Area	Height	Name	Percent_amount
1	RR 3	2.00	12.24000	7.327	493966	94191	Rosin	
2	RR 3	2.00	12.24000	7.332	494524	94567	Rosin	0.3479
3	RR 3	2.00	12.24000	7.356	494460	94358	Rosin	0.3479
Mean								0.3479
Std. Dev.								0.0000
% RSD								0.01

Sign.

Component Results
Name: Rosavin

	SampleName	Injection Volume (uL)	SampleWeight	RT	Area	Height	Name	Percent_amount
1	RR 3	2.00	12.24000	7.608	1121486	216852	Rosavin	
2	RR 3	2.00	12.24000	7.613	1122931	217959	Rosavin	1.0779
3	RR 3	2.00	12.24000	7.637	1123793	217875	Rosavin	1.0787
Mean								1.0783
Std. Dev.								0.0006
% RSD								0.05

Component Results
Name: Cinnamyl alcohol

	SampleName	Injection Volume (uL)	SampleWeight	RT	Area	Height	Name	Percent_amount
1	RR 3	2.00	12.24000	8.835	401640	45524	Cinnamyl alcohol	
2	RR 3	2.00	12.24000	8.843	402127	45611	Cinnamyl alcohol	0.1344
3	RR 3	2.00	12.24000	8.877	403015	45457	Cinnamyl alcohol	0.1347
Mean								0.1345
Std. Dev.								0.0002
% RSD								0.16

Result	***%***	***Calculated***	***Recovery***
p-tyrosol:			
Salidrosid			
Rosarin:			
Rosin:			
Rosavin:			
Cinnamylalcohol:			

Sign.:

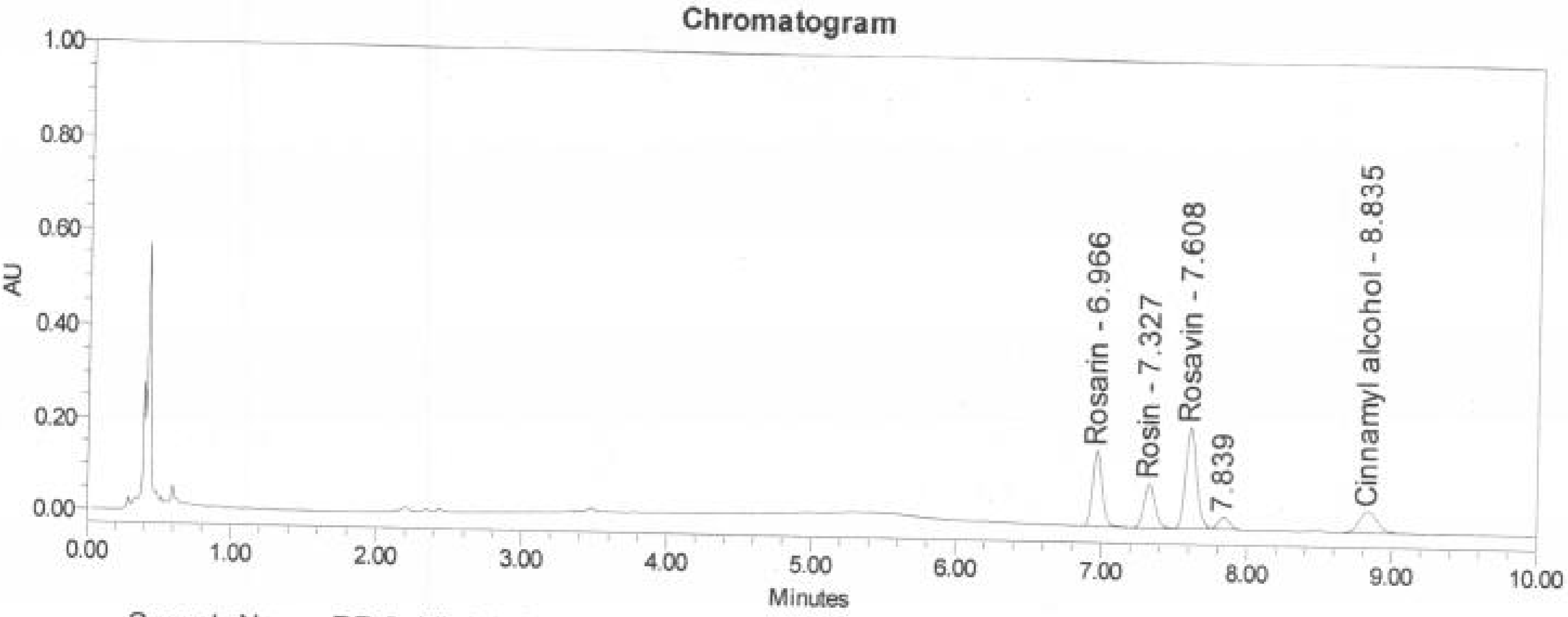

SampleName RR 3; Vial 1:C,3; Date Acquired 2017-05-23 15:54:20 CEST; Channel Name 252 nm

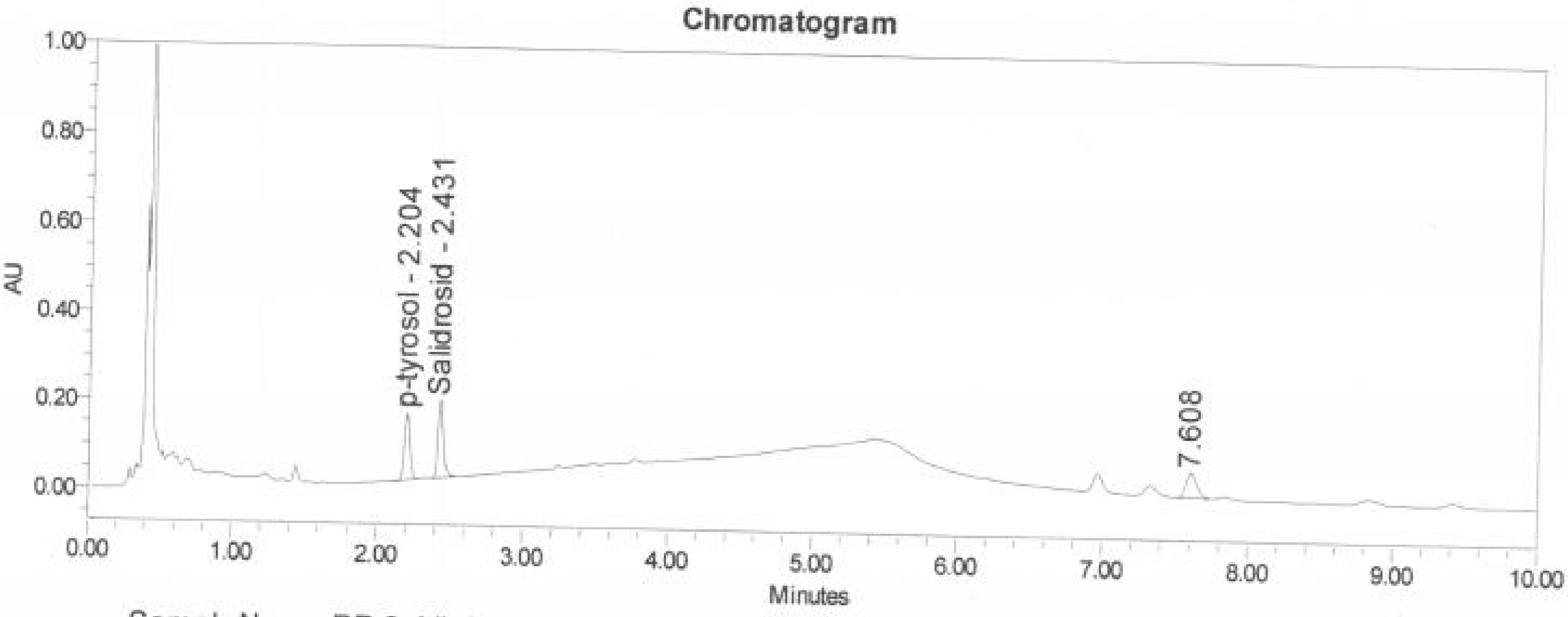

SampleName RR 3; Vial 1:C,3; Date Acquired 2017-05-23 15:54:20 CEST; Channel Name 221 nm

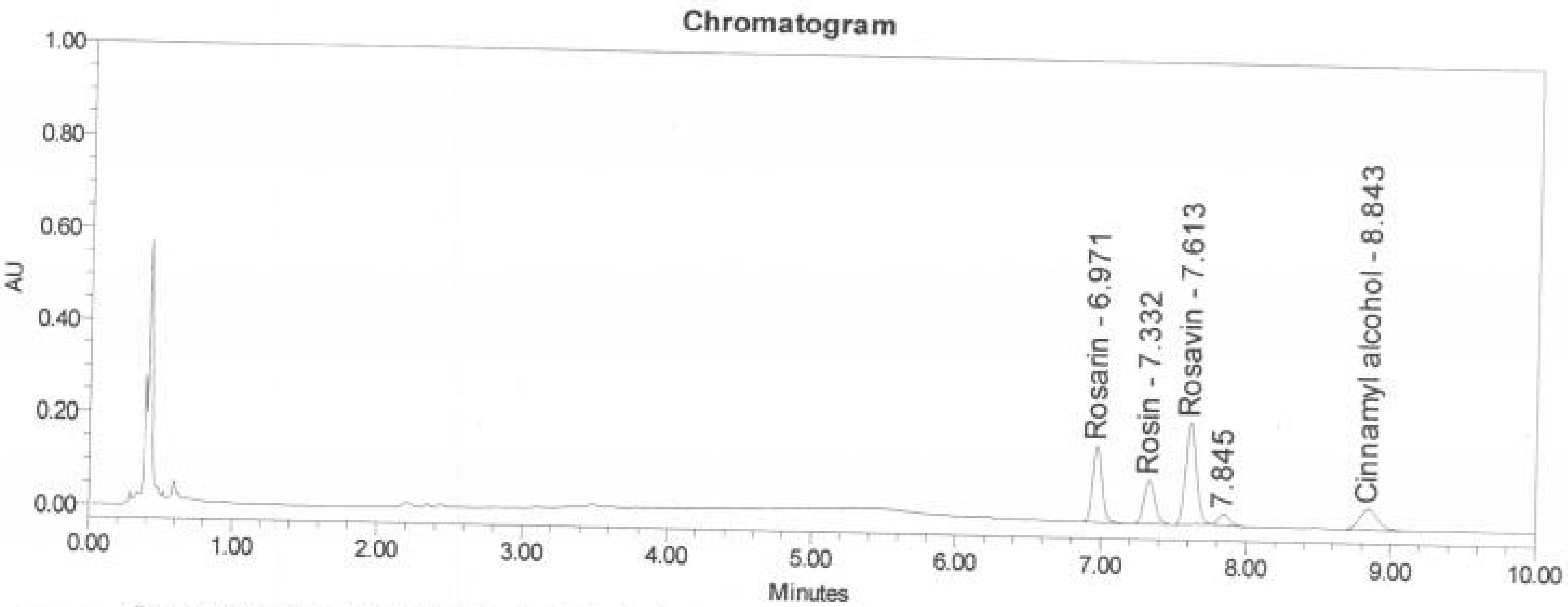

SampleName RR 3; Vial 1:C,3; Date Acquired 2017-05-23 15:14:16 CEST; Channel Name 252 nm

Sign.:

Chromatogram

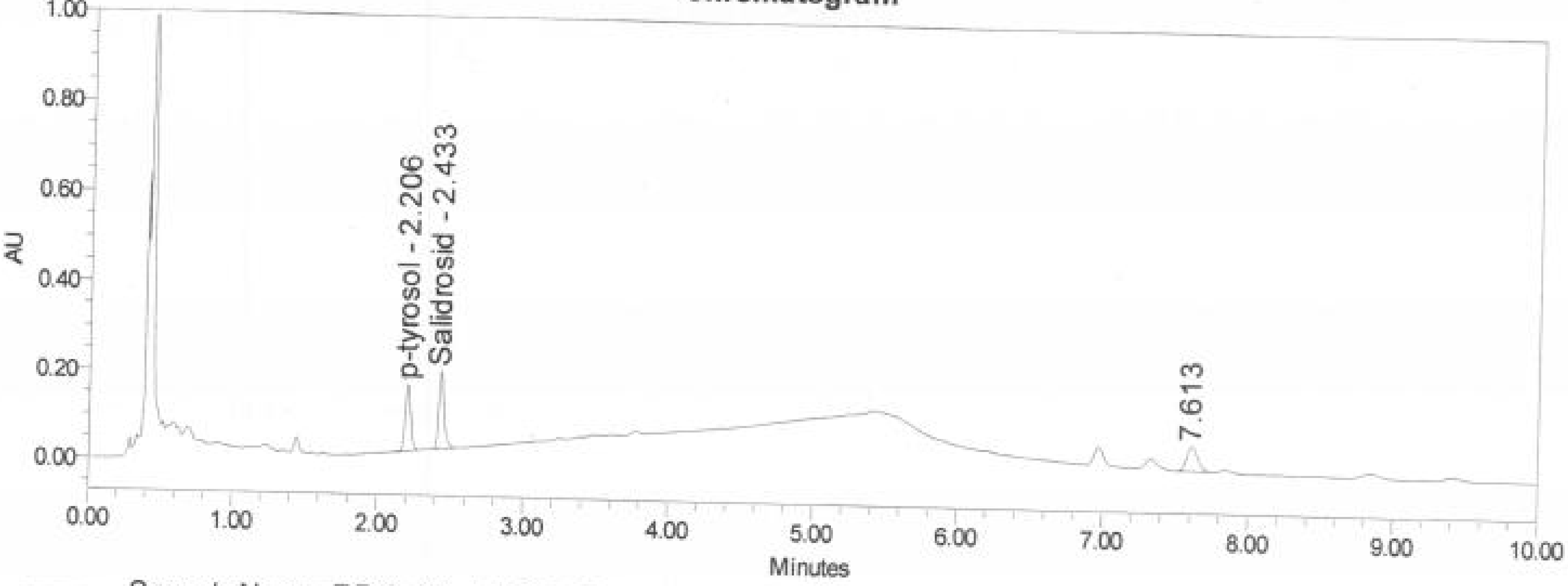

SampleName RR 3; Vial 1:C,3; Date Acquired 2017-05-23 15:14:16 CEST; Channel Name 221 nm

Chromatogram

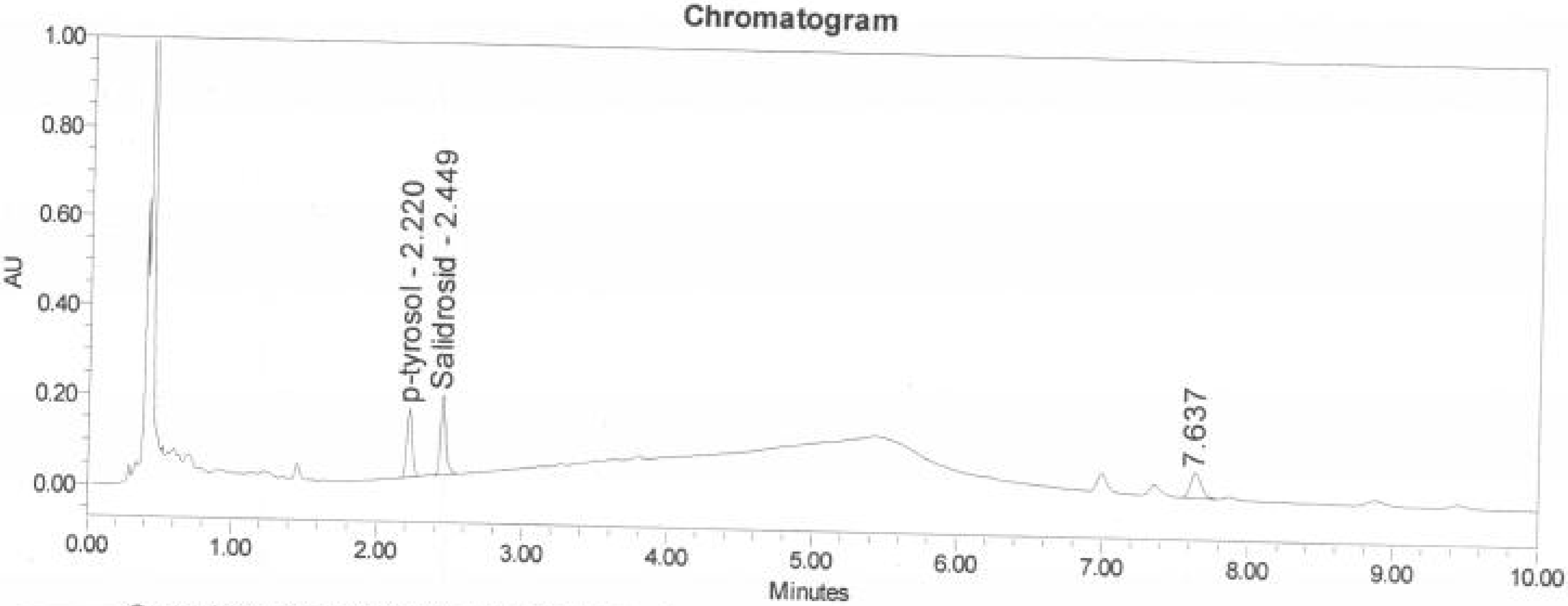

SampleName RR 3; Vial 1:C,3; Date Acquired 2017-05-23 14:34:13 CEST; Channel Name 221 nm

Chromatogram

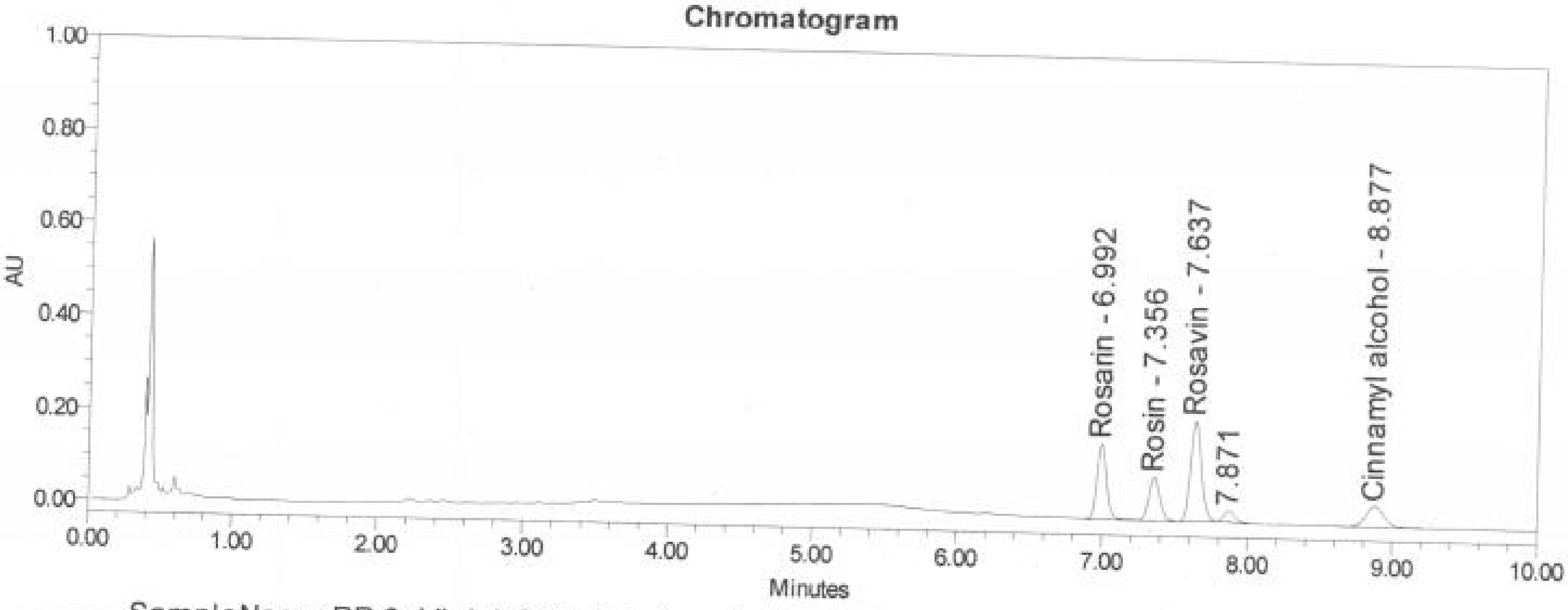

SampleName RR 3; Vial 1:C,3; Date Acquired 2017-05-23 14:34:13 CEST; Channel Name 252 nm

Sign.:

Empower2 software

Sample: RR 4 Chi R Rapport_extrakt_sicc

Method: SM-127		Acquired By:	Alexander
Sample Type:	Unknown	Date Acquired:	2017-05-23 16:34:26 CEST,
Vial:	1:C,4	Acq. Method Set:	Rhodiola 6 markers
Injection #:	1, 2, 3	Date Processed:	2017-05-24 08:30:46 CEST,
Injection Volume:	2.00 ul	Processing Method	Rhodiola_221, Rhodiola_252
Run Time:	10.0 Minutes	Channel Name:	221 nm, 252 nm
Sample Set Name	RR T 0105 UOC	Column no.:	0216331121573E

Component Results
Name: p-tyrosol

	SampleName	Injection Volume (uL)	SampleWeight	RT	Area	Height	Name	Percent_amount
1	RR 4	2.00	11.0500C	2.200	334061	120395	p-tyrosol	0.3294
2	RR 4	2.00	11.0500C	2.203	336260	121092	p-tyrosol	0.3316
3	RR 4	2.00	11.0500C	2.207	337515	121232	p-tyrosol	0.3328
Mean								0.3313
Std. Dev.								0.0017
% RSD								0.52

Component Results
Name: Salidrosid

	SampleName	Injection Volume (uL)	SampleWeight	RT	Area	Height	Name	Percent_amount
1	RR 4	2.00	11.0500C	2.424	1121728	434238	Salidrosid	2.5304
2	RR 4	2.00	11.0500C	2.428	1120922	434957	Salidrosid	2.5286
3	RR 4	2.00	11.0500C	2.432	1122107	434880	Salidrosid	2.5313
Mean								2.5301
Std. Dev.								0.0014
% RSD								0.05

Component Results
Name: Rosarin

	SampleName	Injection Volume (uL)	SampleWeight	RT	Area	Height	Name	Percent_amount
1	RR 4	2.00	11.0500C	6.965	573227	124833	Rosarin	0.6153
2	RR 4	2.00	11.0500C	6.972	573043	124486	Rosarin	0.6151
3	RR 4	2.00	11.0500C	6.977	573952	124386	Rosarin	0.6160
Mean								0.6155
Std. Dev.								0.0005
% RSD								0.08

Component Results
Name: Rosin

	SampleName	Injection Volume (uL)	SampleWeight	RT	Area	Height	Name	Percent_amount
1	RR 4	2.00	11.0500C	7.326	403952	75360	Rosin	0.3144
2	RR 4	2.00	11.0500C	7.334	404102	75180	Rosin	0.3145
3	RR 4	2.00	11.0500C	7.339	404568	75146	Rosin	0.3149
Mean								0.3146
Std. Dev.								0.0003
% RSD								0.08

Sign.: [signature]

Component Results
Name: Rosavin

	SampleName	Injection Volume (uL)	SampleWeight	RT	Area	Height	Name	Percent_amount
1	RR 4	2.00	11.0500C	7.602	2055092	387816	Rosavin	2.1949
2	RR 4	2.00	11.0500C	7.611	2054850	386870	Rosavin	2.1947
3	RR 4	2.00	11.0500C	7.616	2058098	386223	Rosavin	2.1982
Mean								2.1959
Std. Dev.								0.0019
% RSD								0.09

Component Results
Name: Cinnamyl alcohol

	SampleName	Injection Volume (uL)	SampleWeight	RT	Area	Height	Name	Percent_amount
1	RR 4	2.00	11.0500C	8.834	446244	49342	Cinnamyl alcohol	0.1654
2	RR 4	2.00	11.0500C	8.846	445716	49236	Cinnamyl alcohol	0.1652
3	RR 4	2.00	11.0500C	8.854	446717	49213	Cinnamyl alcohol	0.1656
Mean								0.1654
Std. Dev.								0.0002
% RSD								0.11

Result ***%*** ***Calculated*** ***Recovery***

p-tyrosol:

Salidrosid

Rosarin:

Rosin:

Rosavin:

Cinnamylalcohol:

Sign:

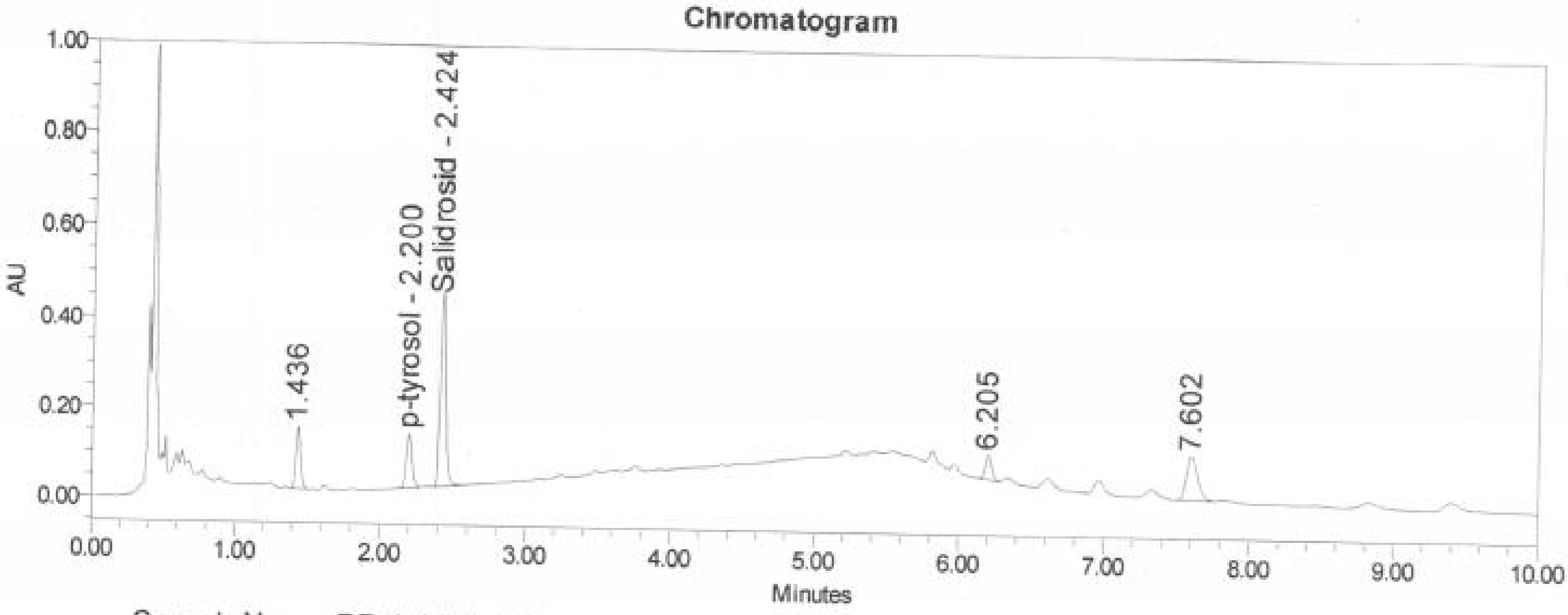

SampleName RR 4; Vial 1:C,4; Date Acquired 2017-05-23 16:34:26 CEST; Channel Name 221 nm

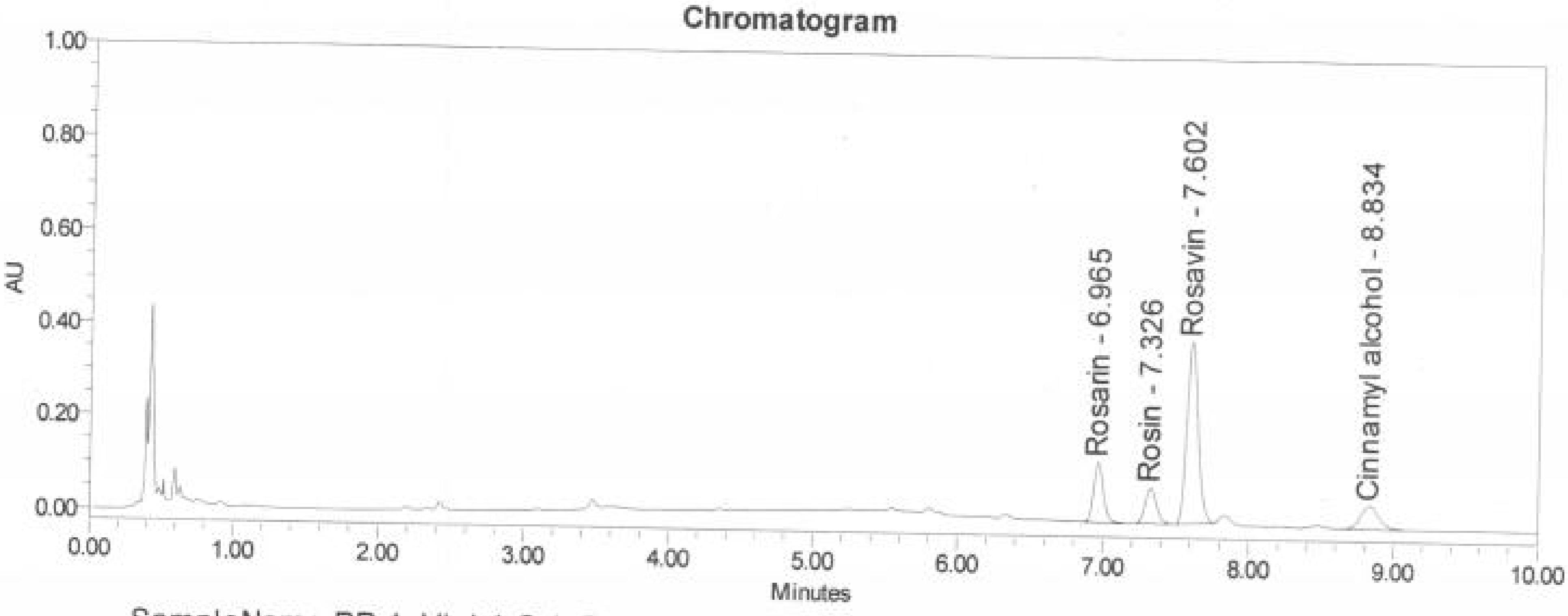

SampleName RR 4; Vial 1:C,4; Date Acquired 2017-05-23 16:34:26 CEST; Channel Name 252 nm

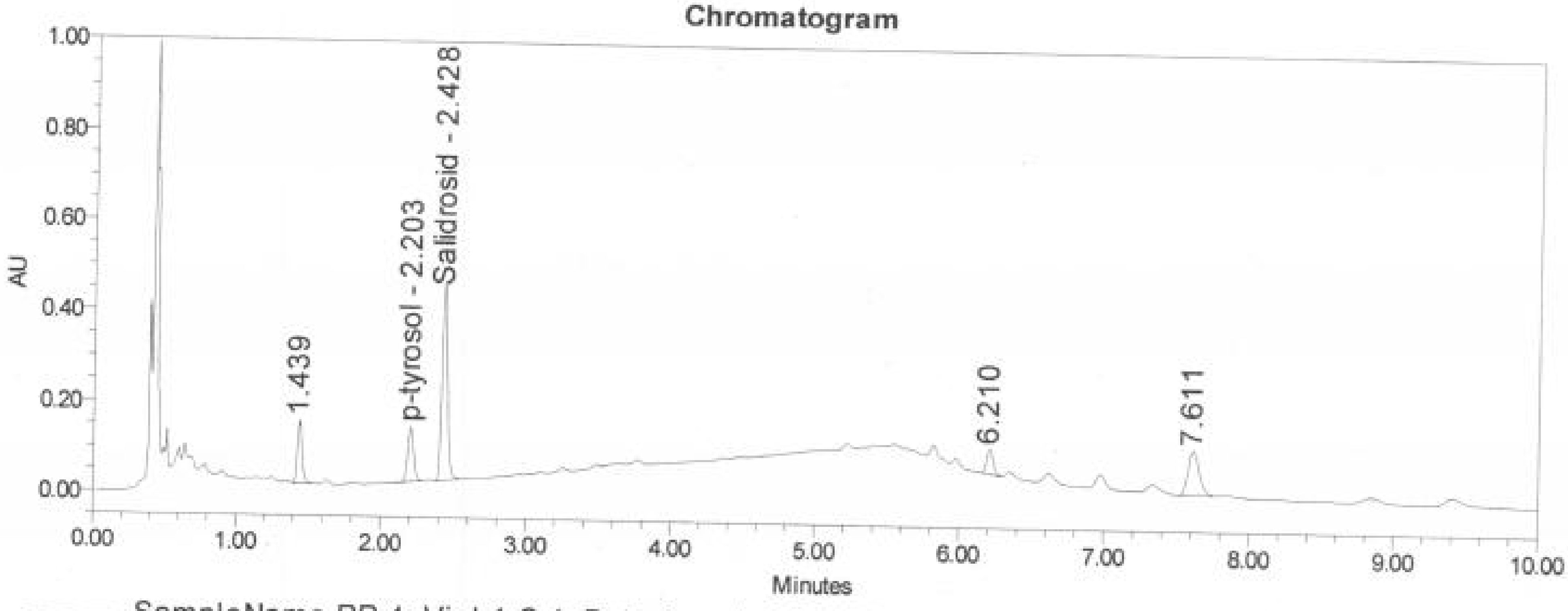

SampleName RR 4; Vial 1:C,4; Date Acquired 2017-05-23 17:14:30 CEST; Channel Name 221 nm

Sign.:

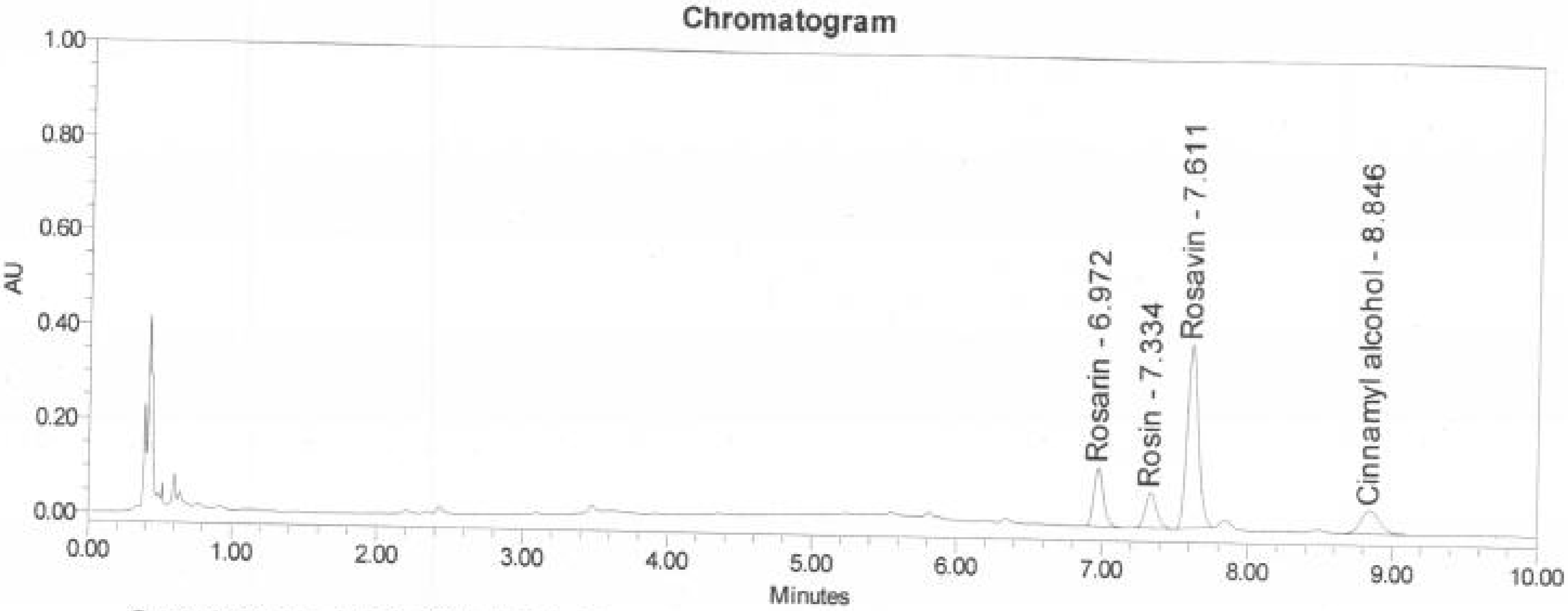

SampleName RR 4; Vial 1:C,4; Date Acquired 2017-05-23 17:14:30 CEST; Channel Name 252 nm

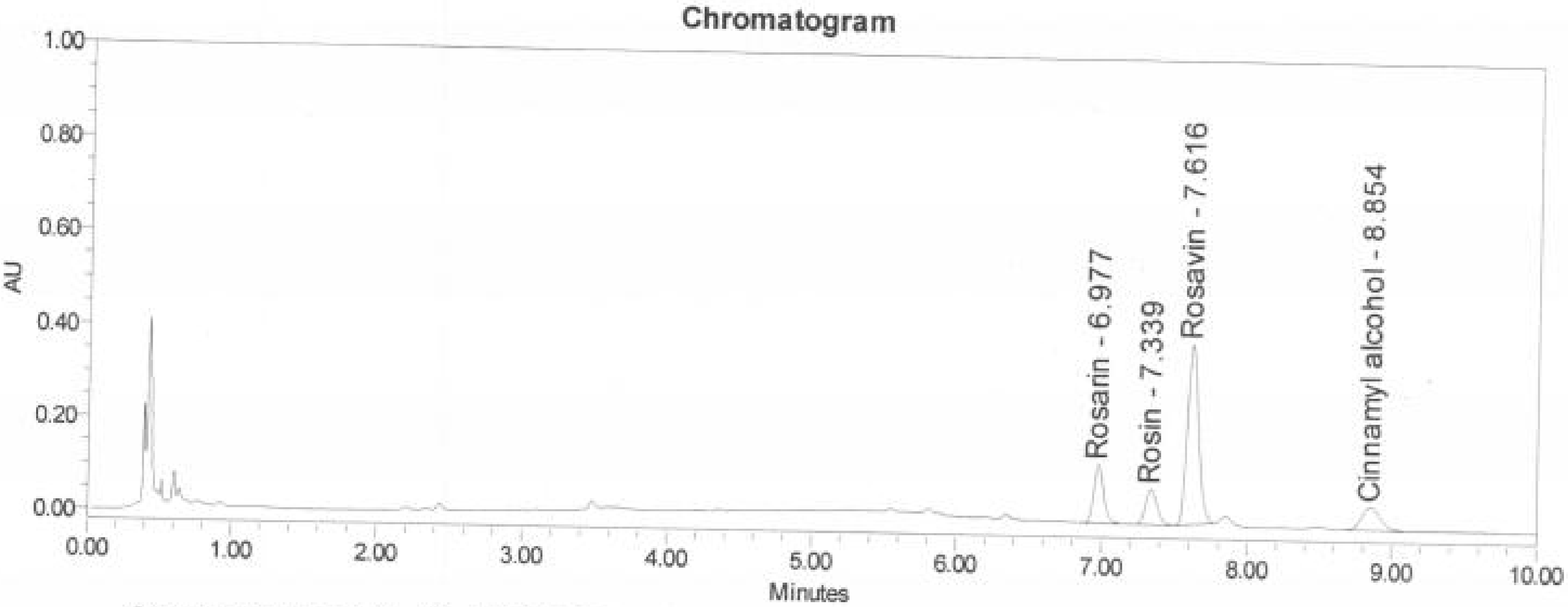

SampleName RR 4; Vial 1:C,4; Date Acquired 2017-05-23 17:54:34 CEST; Channel Name 252 nm

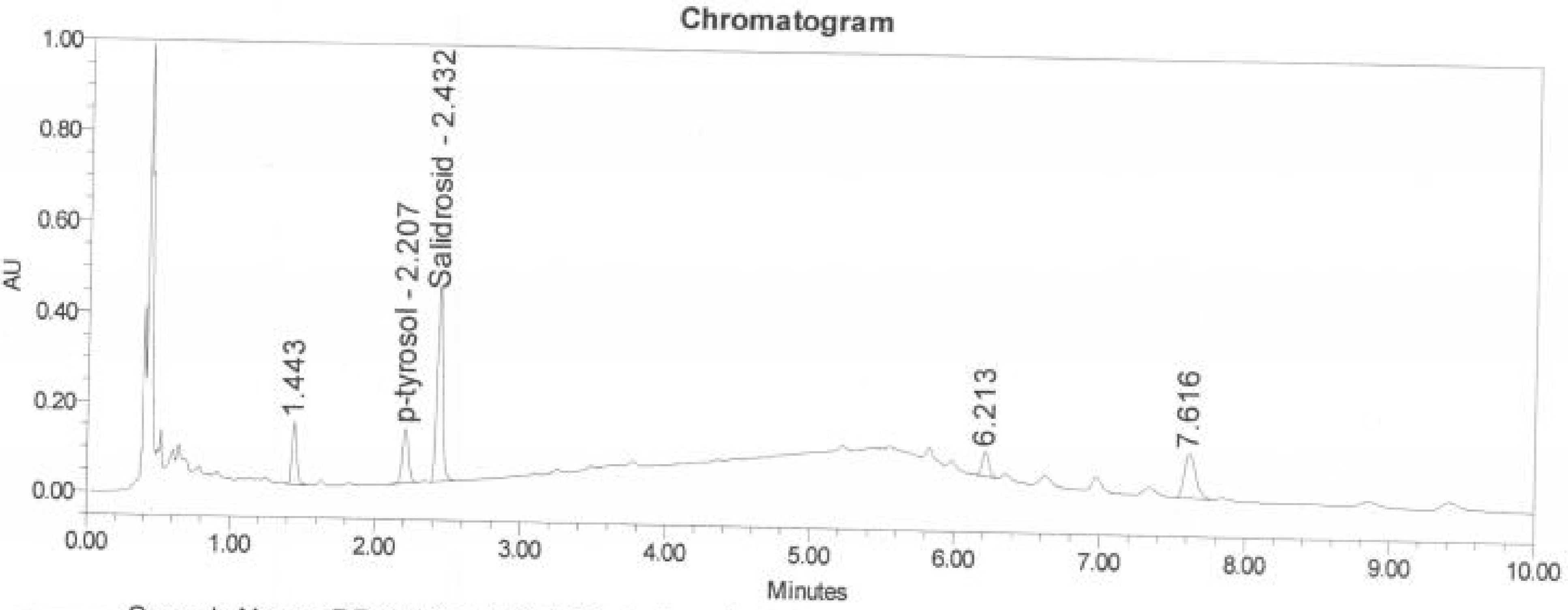

SampleName RR 4; Vial 1:C,4; Date Acquired 2017-05-23 17:54:34 CEST; Channel Name 221 nm

Sign.

Empower2 software

Sample: RR 5 Chi.S Rapport_extrakt_sicc

Method: SM-127		Acquired By:	Alexander
Sample Type:	Unknown	Date Acquired:	2017-05-23 18:34:40 CEST,
Vial:	1:C,5	Acq. Method Set:	Rhodiola 6 markers
Injection #:	1, 2, 3	Date Processed:	2017-05-23 19:13:50 CEST,
Injection Volume:	2.00 ul	Processing Method	Rhodiola_221, Rhodiola_252
Run Time:	10.0 Minutes	Channel Name:	221 nm, 252 nm
Sample Set Name	RR T 0105 UOC	Column no.:	0216331121573ε

Component Results
Name: p-tyrosol

	SampleName	Injection Volume (uL)	SampleWeight	RT	Area	Height	Name	Percent_amount
1	RR 5	2.00	13.1100C	2.207	187498	70412	p-tyrosol	0.1559
2	RR 5	2.00	13.1100C	2.208	187494	69943	p-tyrosol	0.1559
3	RR 5	2.00	13.1100C	2.209	187563	69903	p-tyrosol	0.1560
Mean								0.1559
Std. Dev.								0.0000
% RSD								0.02

Component Results
Name: Salidrosid

	SampleName	Injection Volume (uL)	SampleWeight	RT	Area	Height	Name	Percent_amount
1	RR 5	2.00	13.1100C	2.433	595515	233488	Salidrosid	1.1406
2	RR 5	2.00	13.1100C	2.434	595305	231948	Salidrosid	1.1402
3	RR 5	2.00	13.1100C	2.434	598818	232025	Salidrosid	1.1469
Mean								1.1426
Std. Dev.								0.0037
% RSD								0.33

Component Results
Name: Rosarin

	SampleName	Injection Volume (uL)	SampleWeight	RT	Area	Height	Name	Percent_amount
1	RR 5	2.00	13.1100C	6.721			Rosarin	
2	RR 5	2.00	13.1100C	6.721			Rosarin	
3	RR 5	2.00	13.1100C	6.721			Rosarin	
Mean								
Std. Dev.								
% RSD								

Component Results
Name: Rosin

	SampleName	Injection Volume (uL)	SampleWeight	RT	Area	Height	Name	Percent_amount
1	RR 5	2.00	13.1100C	7.034			Rosin	
2	RR 5	2.00	13.1100C	7.034			Rosin	
3	RR 5	2.00	13.1100C	7.034			Rosin	
Mean								
Std. Dev.								
% RSD								

Sign.:

Component Results
Name: Rosavin

	SampleName	Injection Volume (uL)	SampleWeight	RT	Area	Height	Name	Percent_amount
1	RR 5	2.00	13.1100C	7.339			Rosavin	
2	RR 5	2.00	13.1100C	7.339			Rosavin	
3	RR 5	2.00	13.1100C	7.339			Rosavin	
Mean								
Std. Dev.								
% RSD								

Component Results
Name: Cinnamyl alcohol

	SampleName	Injection Volume (uL)	SampleWeight	RT	Area	Height	Name	Percent_amount
1	RR 5	2.00	13.1100C	8.500			Cinnamyl alcohol	
2	RR 5	2.00	13.1100C	8.500			Cinnamyl alcohol	
3	RR 5	2.00	13.1100C	8.500			Cinnamyl alcohol	
Mean								
Std. Dev.								
% RSD								

Result % Calculated Recovery

p-tyrosol:

Salidrosid

Rosarin:

Rosin:

Rosavin:

Cinnamylalcohol:

Sign.:

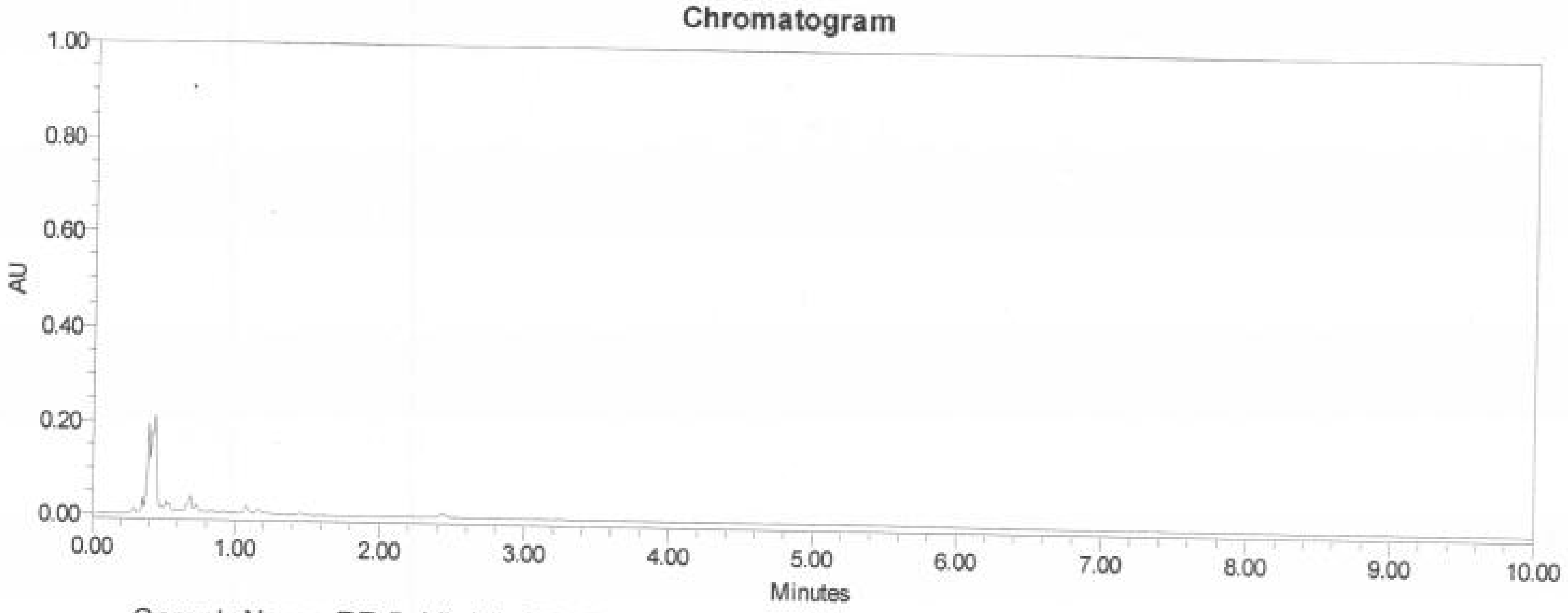

SampleName RR 5; Vial 1:C,5; Date Acquired 2017-05-23 19:54:47 CEST; Channel Name 252 nm

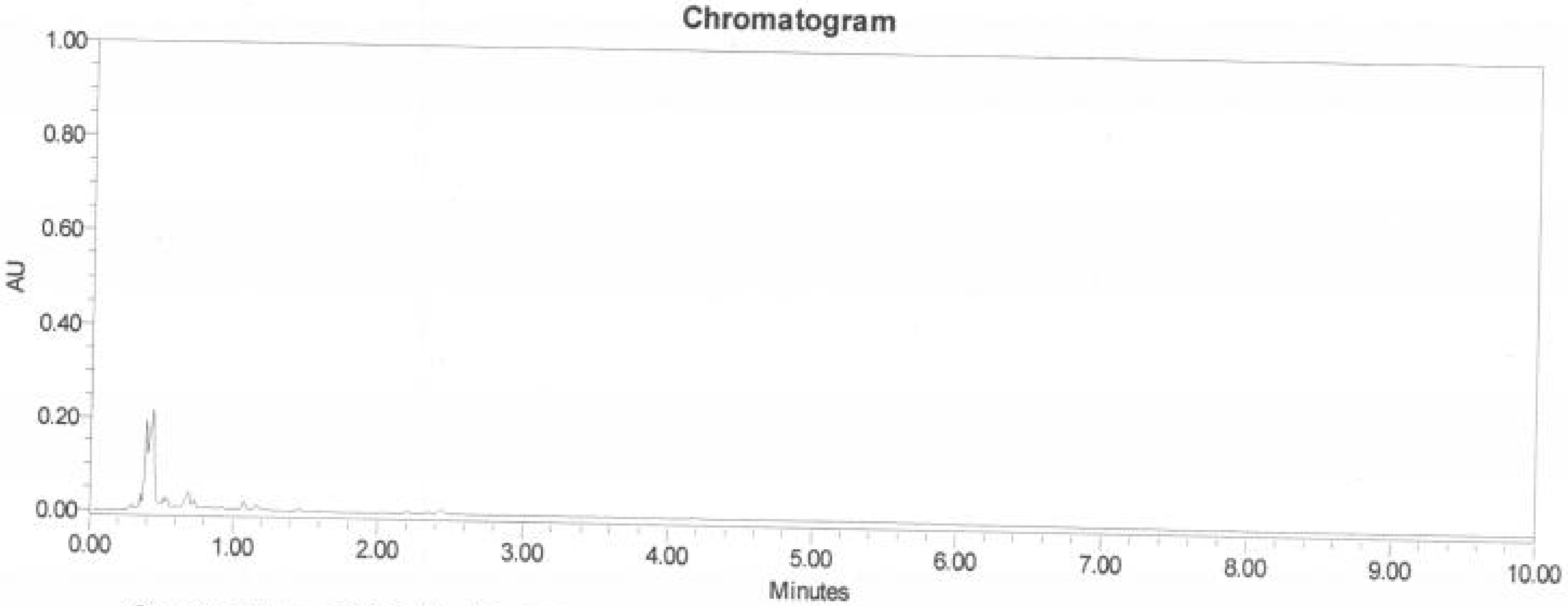

SampleName RR 5; Vial 1:C,5; Date Acquired 2017-05-23 19:14:43 CEST; Channel Name 252 nm

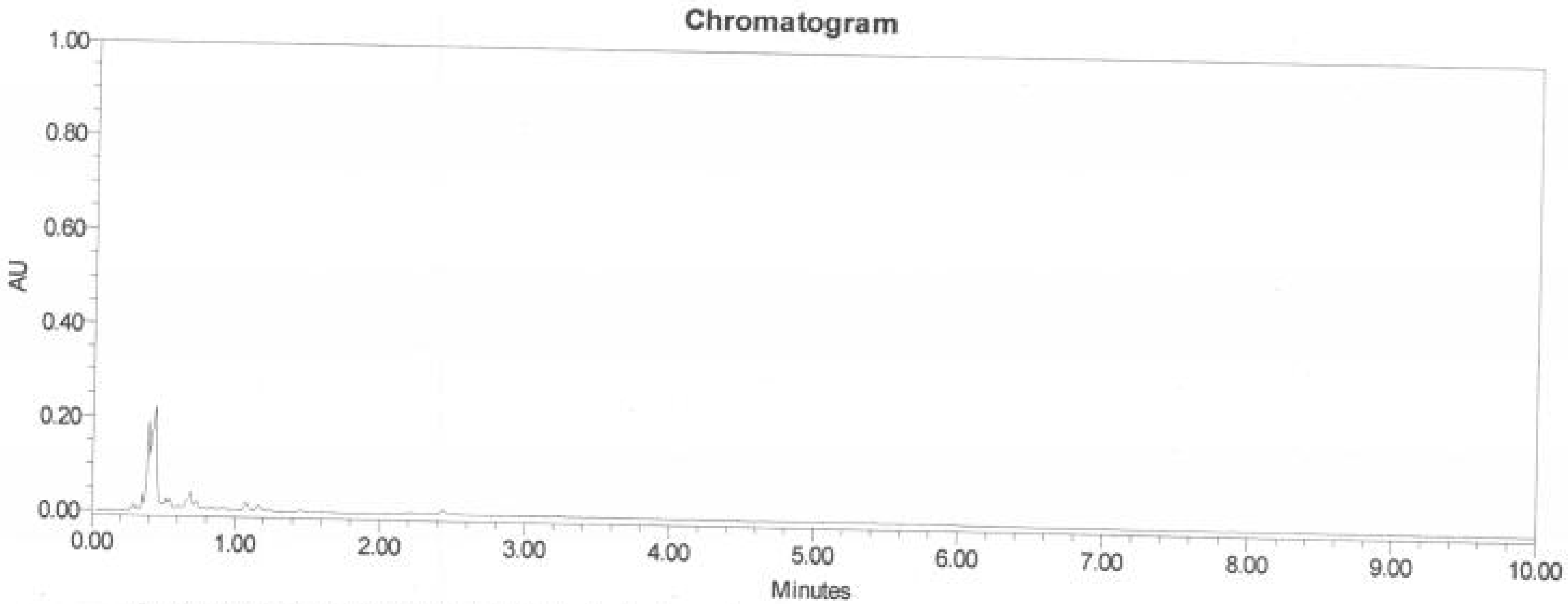

SampleName RR 5; Vial 1:C,5; Date Acquired 2017-05-23 18:34:40 CEST; Channel Name 252 nm

Sign.:

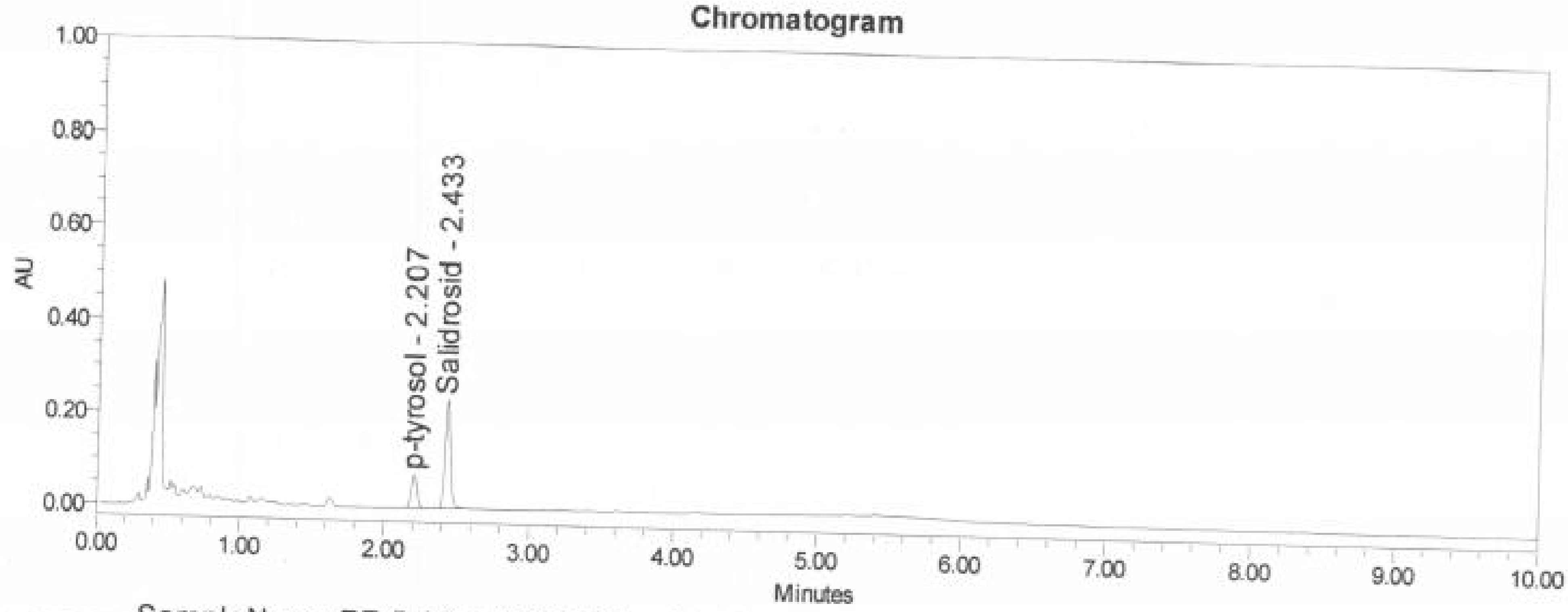

SampleName RR 5; Vial 1:C,5; Date Acquired 2017-05-23 18:34:40 CEST; Channel Name 221 nm

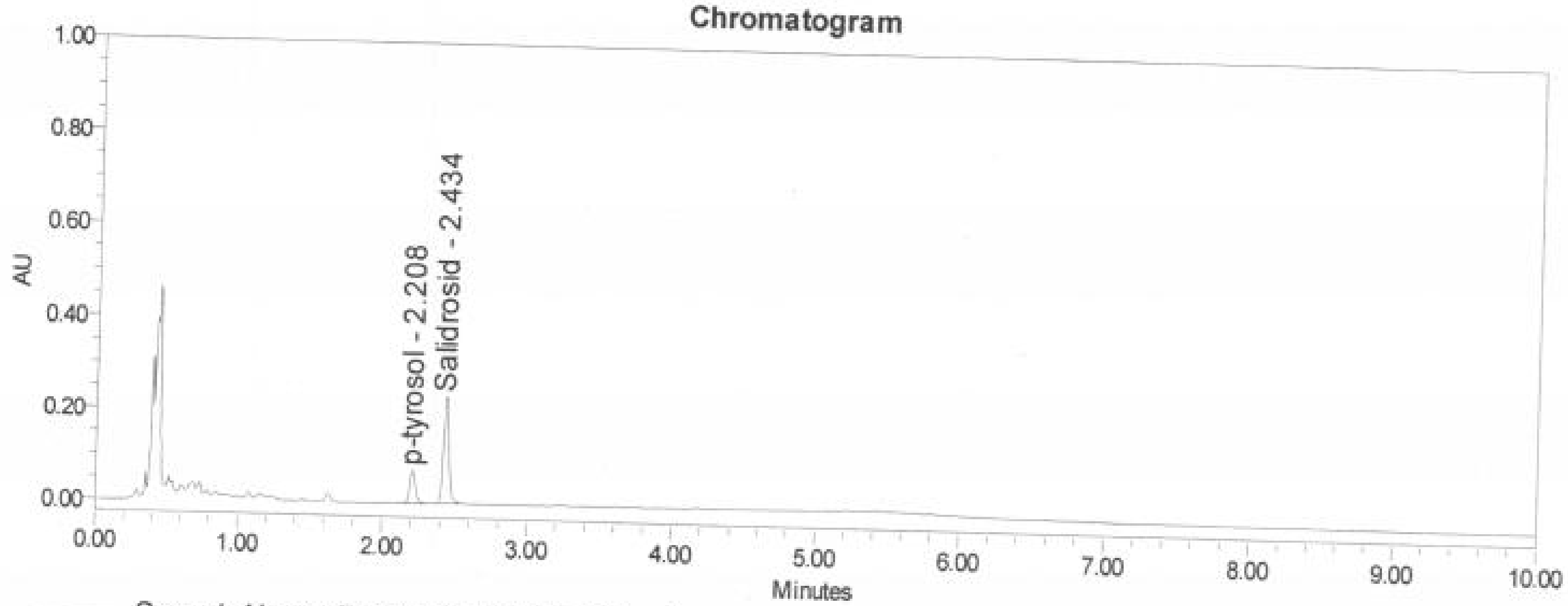

SampleName RR 5; Vial 1:C,5; Date Acquired 2017-05-23 19:14:43 CEST; Channel Name 221 nm

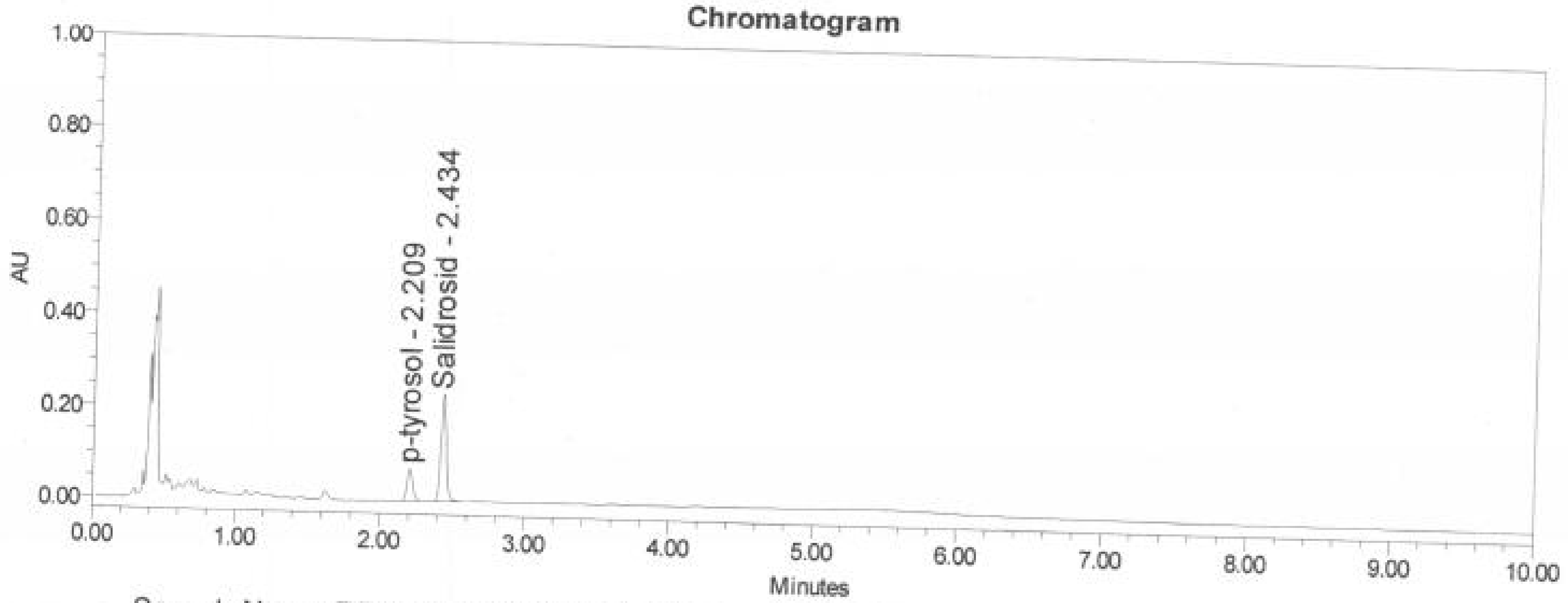

SampleName RR 5; Vial 1:C,5; Date Acquired 2017-05-23 19:54:47 CEST; Channel Name 221 nm

Sign.:

Empower2 software

Sample: RR 6 EPR-7 Rapport_extrakt_sicc

Method:	SM-127	Acquired By:	Alexander
Sample Type:	Unknown	Date Acquired:	2017-05-23 20:34:51 CEST,
Vial:	1:C,6	Acq. Method Set:	Rhodiola 6 markers
Injection #:	1, 2, 3	Date Processed:	2017-05-24 08:32:36 CEST,
Injection Volume:	2.00 ul	Processing Method	Rhodiola_221, Rhodiola_252
Run Time:	10.0 Minutes	Channel Name:	221 nm, 252 nm
Sample Set Name	RR T 0105 UOC	Column no.:	02163311215738

Component Results
Name: p-tyrosol

	SampleName	Injection Volume (uL)	SampleWeight	RT	Area	Height	Name	Percent_amount
1	RR 6	2.00	15.59000	2.200	652149	247043	p-tyrosol	0.4556
2	RR 6	2.00	15.59000	2.203	652306	245663	p-tyrosol	0.4557
3	RR 6	2.00	15.59000	2.206	653669	246319	p-tyrosol	0.4567
Mean								0.4560
Std. Dev.								0.0006
% RSD								0.13

Component Results
Name: Salidrosid

	SampleName	Injection Volume (uL)	SampleWeight	RT	Area	Height	Name	Percent_amount
1	RR 6	2.00	15.59000	2.423	1939348	761766	Salidrosid	3.0899
2	RR 6	2.00	15.59000	2.424	1933336	752786	Salidrosid	3.0804
3	RR 6	2.00	15.59000	2.427	1935209	754600	Salidrosid	3.0834
Mean								3.0846
Std. Dev.								0.0049
% RSD								0.16

Component Results
Name: Rosarin

	SampleName	Injection Volume (uL)	SampleWeight	RT	Area	Height	Name	Percent_amount
1	RR 6	2.00	15.59000	6.968	1241134	271198	Rosarin	0.9495
2	RR 6	2.00	15.59000	6.973	1242988	271890	Rosarin	0.9509
3	RR 6	2.00	15.59000	6.983	1275276	271552	Rosarin	0.9757
Mean								0.9587
Std. Dev.								0.0148
% RSD								1.54

Component Results
Name: Rosin

	SampleName	Injection Volume (uL)	SampleWeight	RT	Area	Height	Name	Percent_amount
1	RR 6	2.00	15.59000	7.333	730064	136736	Rosin	0.4040
2	RR 6	2.00	15.59000	7.337	731617	136731	Rosin	0.4049
3	RR 6	2.00	15.59000	7.348	692046	133274	Rosin	0.3829
Mean								0.3972
Std. Dev.								0.0125
% RSD								3.13

Sign.: [signature]

Component Results
Name: Rosavin

	SampleName	Injection Volume (uL)	SampleWeight	RT	Area	Height	Name	Percent_amount
1	RR 6	2.00	15.59000	7.597	4853137	1239388	Rosavin	3.6854
2	RR 6	2.00	15.59000	7.599	4820851	905756	Rosavin	3.6608
3	RR 6	2.00	15.59000	7.606	4835247	910068	Rosavin	3.6718
Mean								3.6727
Std. Dev.								0.0123
% RSD								0.34

Component Results
Name: Cinnamyl alcohol

	SampleName	Injection Volume (uL)	SampleWeight	RT	Area	Height	Name	Percent_amount
1	RR 6	2.00	15.59000	8.500			Cinnamyl alcohol	
2	RR 6	2.00	15.59000	8.500			Cinnamyl alcohol	
3	RR 6	2.00	15.59000	8.500			Cinnamyl alcohol	
Mean								
Std. Dev.								
% RSD								

Result	%	Calculated	Recovery
p-tyrosol:			
Salidrosid			
Rosarin:			
Rosin:			
Rosavin:			
Cinnamylalcohol:			

Sign.:

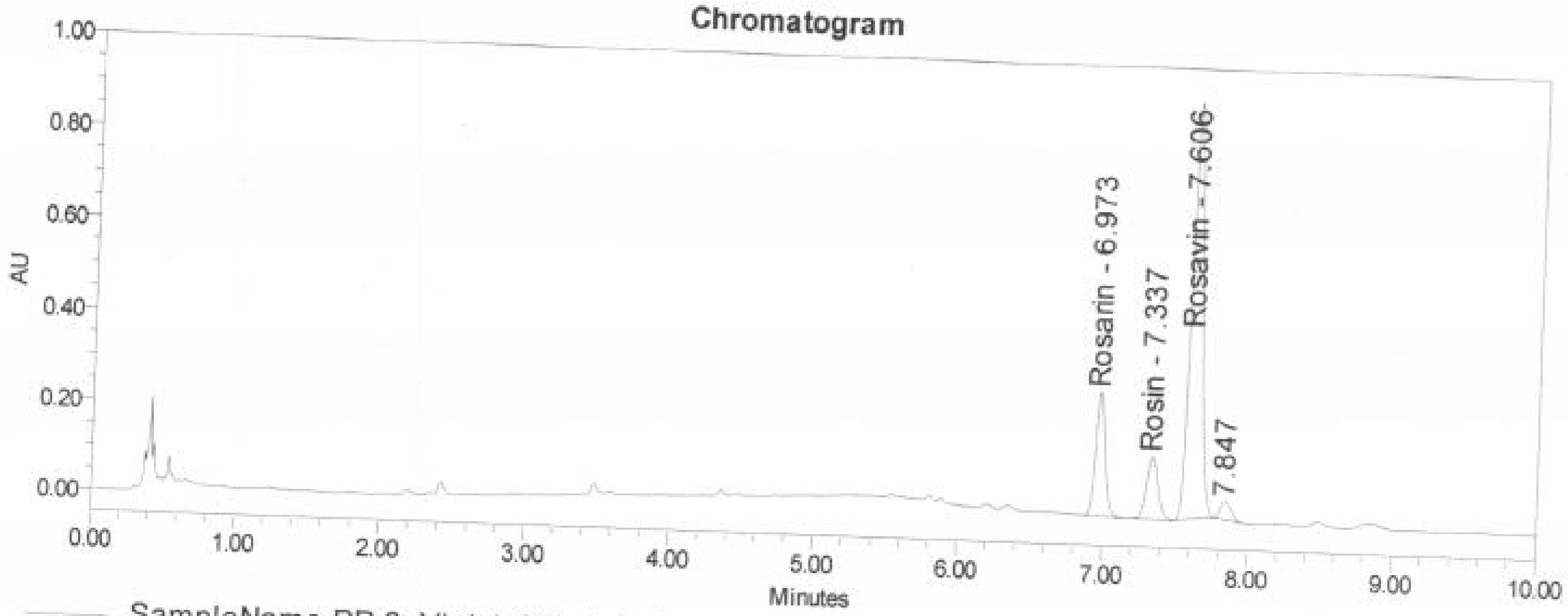

SampleName RR 6; Vial 1:C,6; Date Acquired 2017-05-23 20:34:51 CEST; Channel Name 252 nm

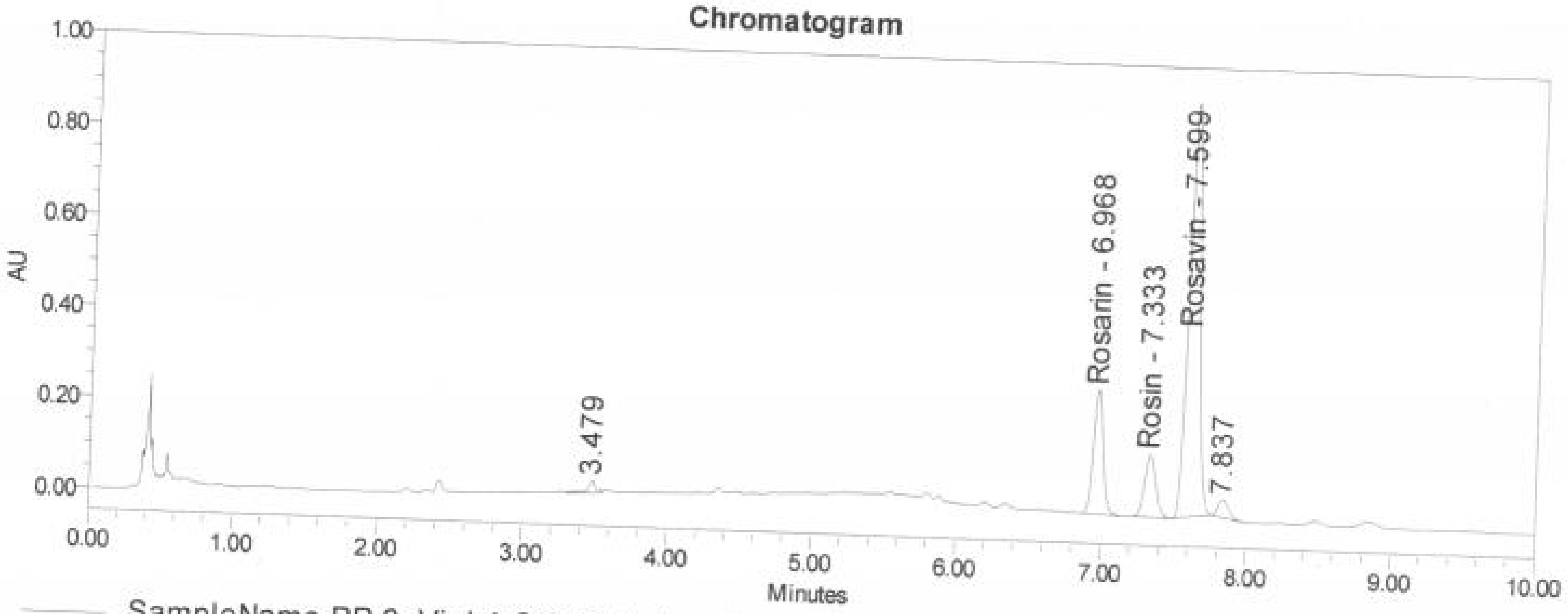

SampleName RR 6; Vial 1:C,6; Date Acquired 2017-05-23 21:54:58 CEST; Channel Name 252 nm

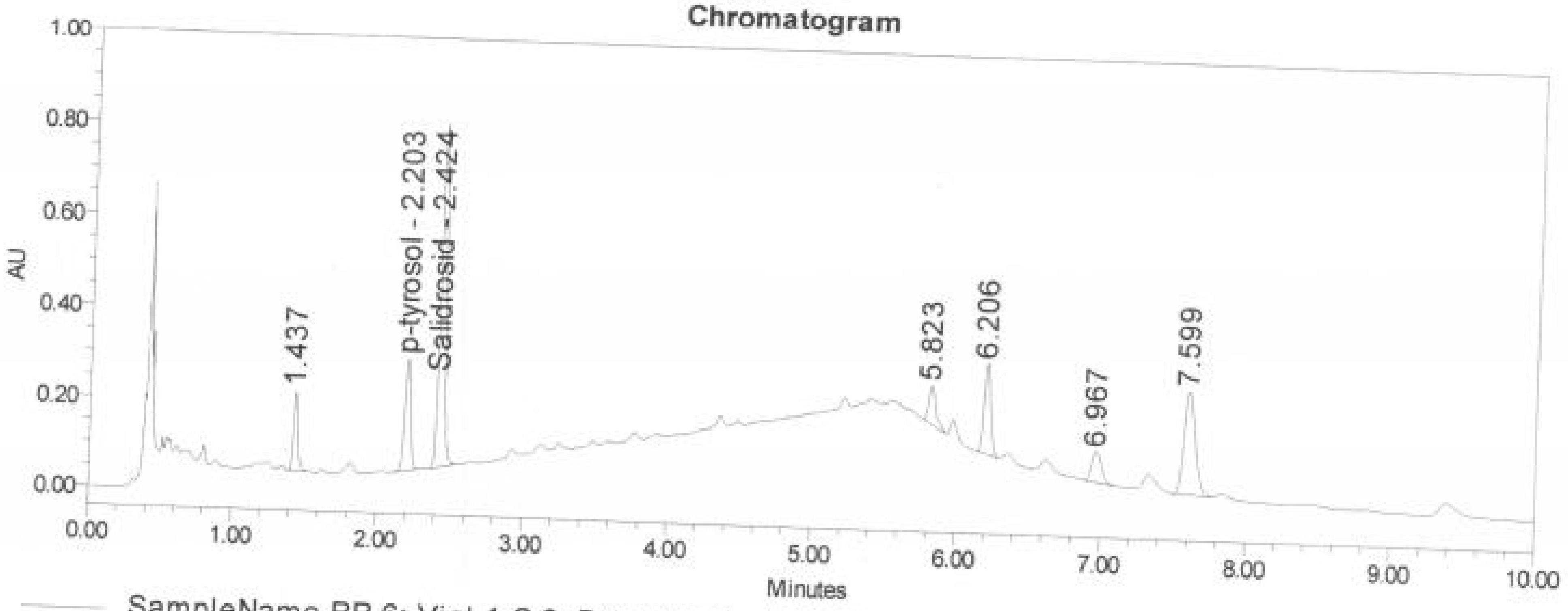

SampleName RR 6; Vial 1:C,6; Date Acquired 2017-05-23 21:54:58 CEST; Channel Name 221 nm

Sign.:

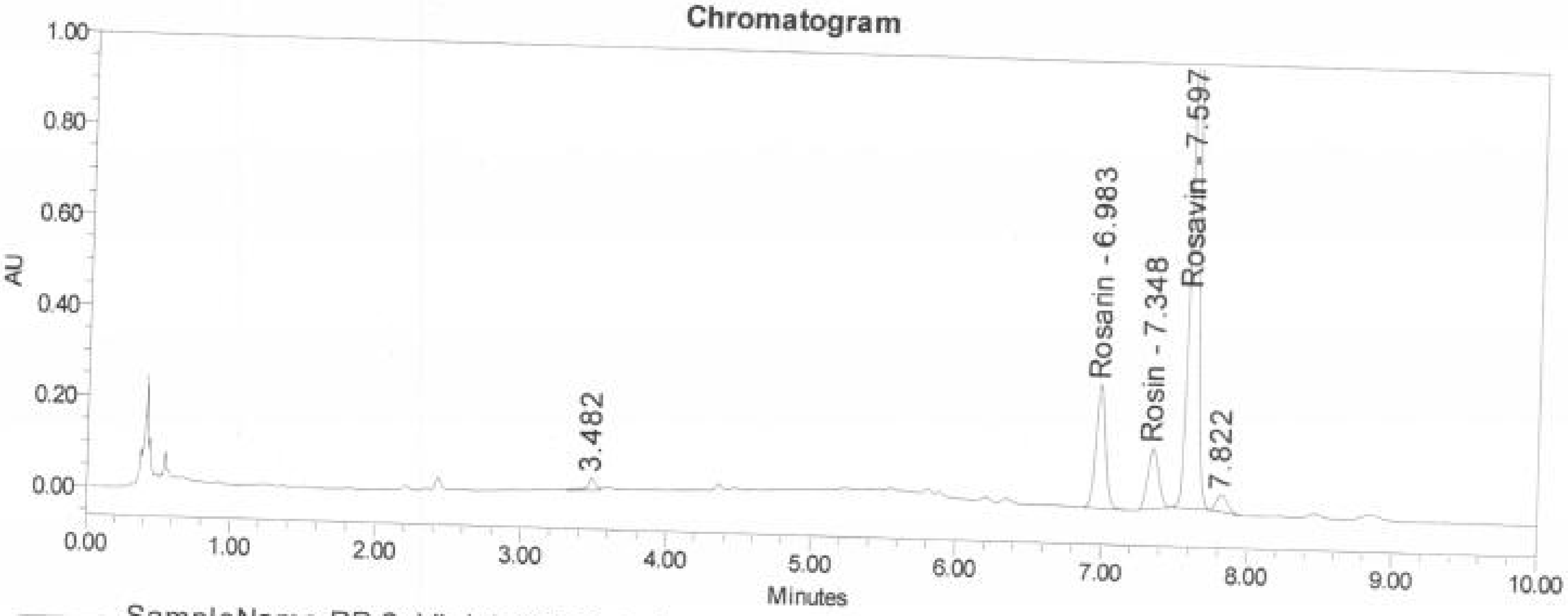

SampleName RR 6; Vial 1:C,6; Date Acquired 2017-05-23 21:14:55 CEST; Channel Name 252 nm

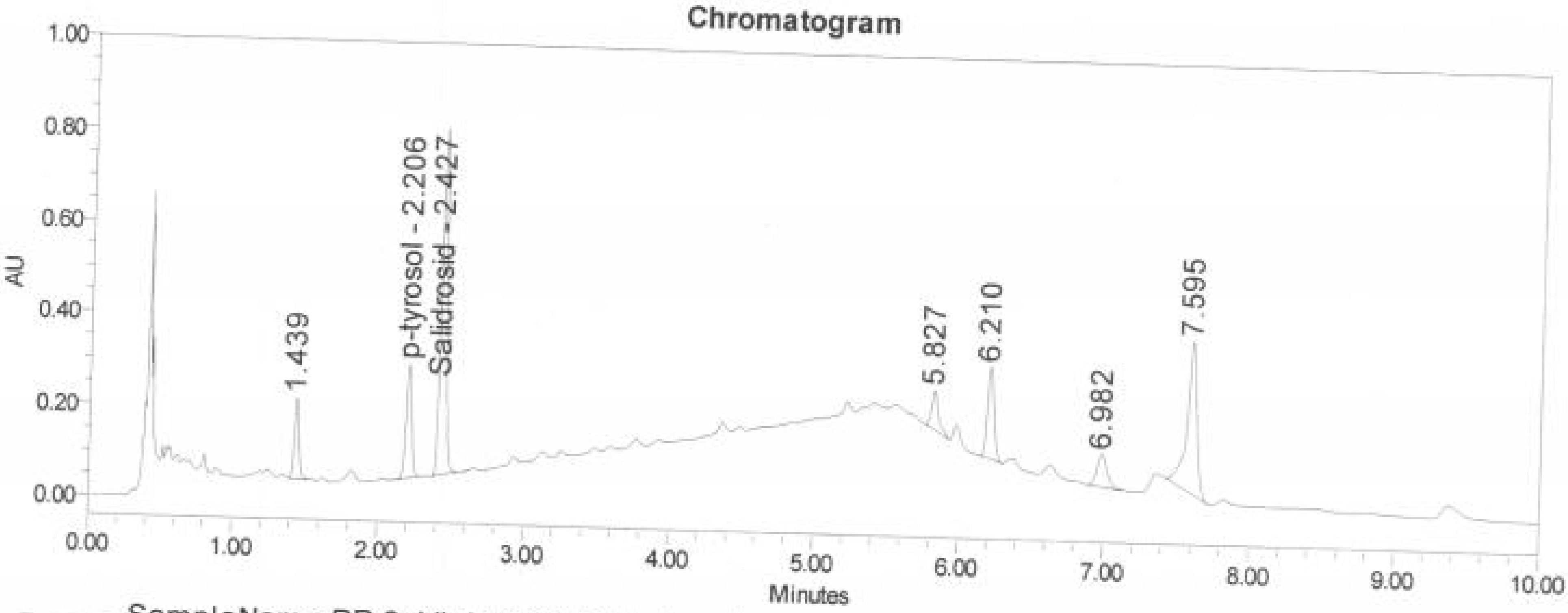

SampleName RR 6; Vial 1:C,6; Date Acquired 2017-05-23 21:14:55 CEST; Channel Name 221 nm

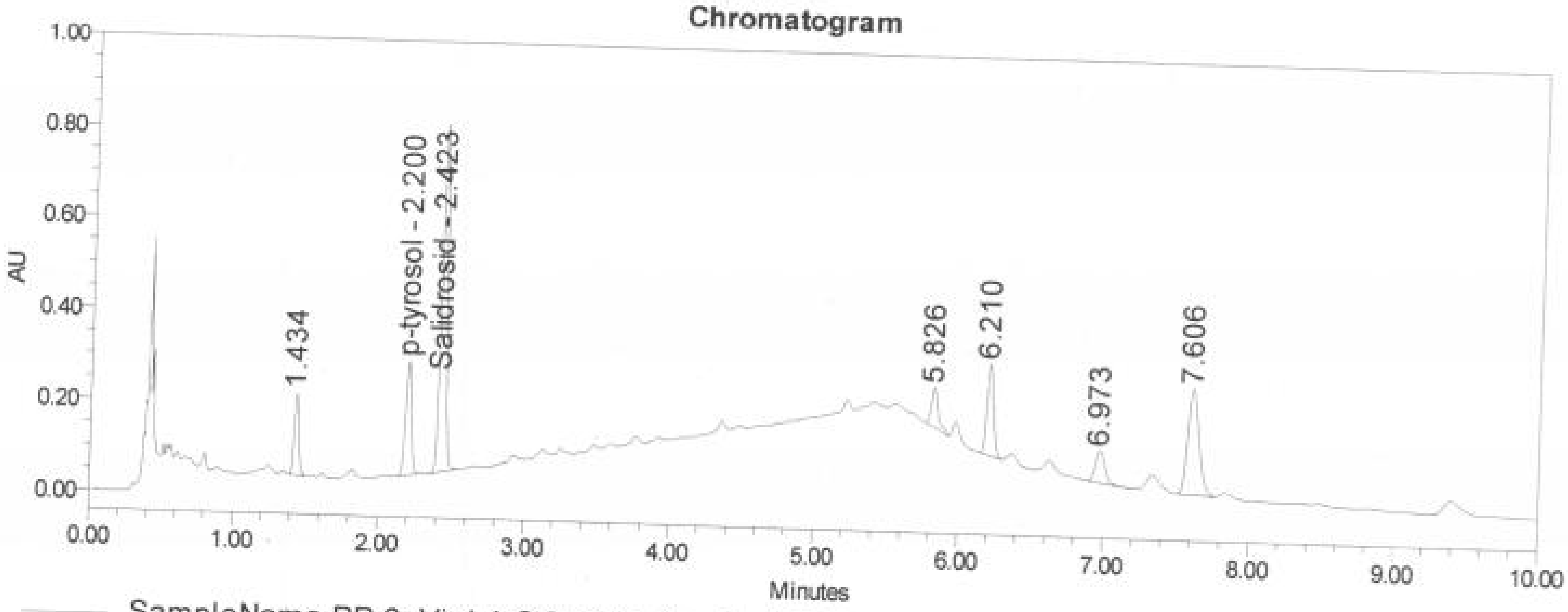

SampleName RR 6; Vial 1:C,6; Date Acquired 2017-05-23 20:34:51 CEST; Channel Name 221 nm

Sign.:

Empower2 software

Sample: RR scc 325 - SHR·5 Rapport_extrakt_sicc

Method: SM-127		Acquired By:	Alexander
Sample Type:	Unknown	Date Acquired:	2016-11-23 11:29:33 CET,
Vial:	1:A,3	Acq. Method Set:	Rhodiola 6 markers
Injection #:	1, 2, 3	Date Processed:	2016-11-23 12:52:42 CET,
Injection Volume:	2.00 ul	Processing Method	Rhodiola_221, Rhodiola_252
Run Time:	10.0 Minutes	Channel Name:	221 nm, 252 nm
Sample Set Name	VILAR	Column no.:	0216331121573[illegible]

Component Results
Name: p-tyrosol

	SampleName	Injection Volume (uL)	SampleWeight	RT	Area	Height	Name	Percent_amount
1	RR scc 325	2.00	13.03600	2.238	177338	74841	p-tyrosol	0.1483
2	RR scc 325	2.00	13.03600	2.239	177980	74940	p-tyrosol	0.1488
3	RR scc 325	2.00	13.03600	2.247	176758	74602	p-tyrosol	0.1478
Mean								0.1483
Std. Dev.								0.0005
% RSD								0.34

Component Results
Name: Salidrosid

	SampleName	Injection Volume (uL)	SampleWeight	RT	Area	Height	Name	Percent_amount
1	RR scc 325	2.00	13.03600	2.471	114067[illegible]	481421	Salidrosid	2.1809
2	RR scc 325	2.00	13.03600	2.474	111230[illegible]	478337	Salidrosid	2.1271
3	RR scc 325	2.00	13.03600	2.488	110448[illegible]	476428	Salidrosid	2.1122
Mean								2.1400
Std. Dev.								0.0361
% RSD								1.69

Component Results
Name: Rosarin

	SampleName	Injection Volume (uL)	SampleWeight	RT	Area	Height	Name	Percent_amount
1	RR scc 325	2.00	13.03600	7.126	1177871	300213	Rosarin	1.0773
2	RR scc 325	2.00	13.03600	7.137	1179841	301062	Rosarin	1.0791
3	RR scc 325	2.00	13.03600	7.183	1181922	297299	Rosarin	1.0810
Mean								1.0792
Std. Dev.								0.0019
% RSD								0.17

Component Results
Name: Rosin

	SampleName	Injection Volume (uL)	SampleWeight	RT	Area	Height	Name	Percent_amount
1	RR scc 325	2.00	13.03600	7.477	887179	188934	Rosin	0.5875
2	RR scc 325	2.00	13.03600	7.487	883631	189570	Rosin	0.5852
3	RR scc 325	2.00	13.03600	7.530	887306	187841	Rosin	0.5876
Mean								0.5868
Std. Dev.								0.0014
% RSD								0.24

Sign.

Component Results
Name: Rosavin

	SampleName	Injection Volume (uL)	SampleWeight	RT	Area	Height	Name	Percent_amount
1	RR scc 325	2.00	13.03600	7.814	341511[illegible]	740384	Rosavin	3.0985
2	RR scc 325	2.00	13.03600	7.825	3416772	744699	Rosavin	3.1000
3	RR scc 325	2.00	13.03600	7.879	3420523	732412	Rosavin	3.1034
Mean								3.1006
Std. Dev.								0.0025
% RSD								0.08

Component Results
Name: Cinnamyl alcohol

	SampleName	Injection Volume (uL)	SampleWeight	RT	Area	Height	Name	Percent_amount
1	RR scc 325	2.00	13.03600	9.009	114808[illegible]	145929	Cinnamyl alcohol	0.3632
2	RR scc 325	2.00	13.03600	9.017	114395[illegible]	145433	Cinnamyl alcohol	0.3619
3	RR scc 325	2.00	13.03600	9.053	111518[illegible]	142909	Cinnamyl alcohol	0.3528
Mean								0.3593
Std. Dev.								0.0057
% RSD								1.59

**Result**	**% Calculated**	**Recovery** ± %
**p-tyrosol:**	0,15 ± 0,0005	0,34
**Salidrosid**	2,14 ± 0,0361	1,69
**Rosarin:**	1,08 ± 0,0019	0,17
**Rosin:**	0,59 ± 0,0014	0,24
**Rosavin:**	3,10 ± 0.0025	0,08
**Cinnamylalcohol:**	0,36 ± 0,0057	1,59

Sign.: [signature]

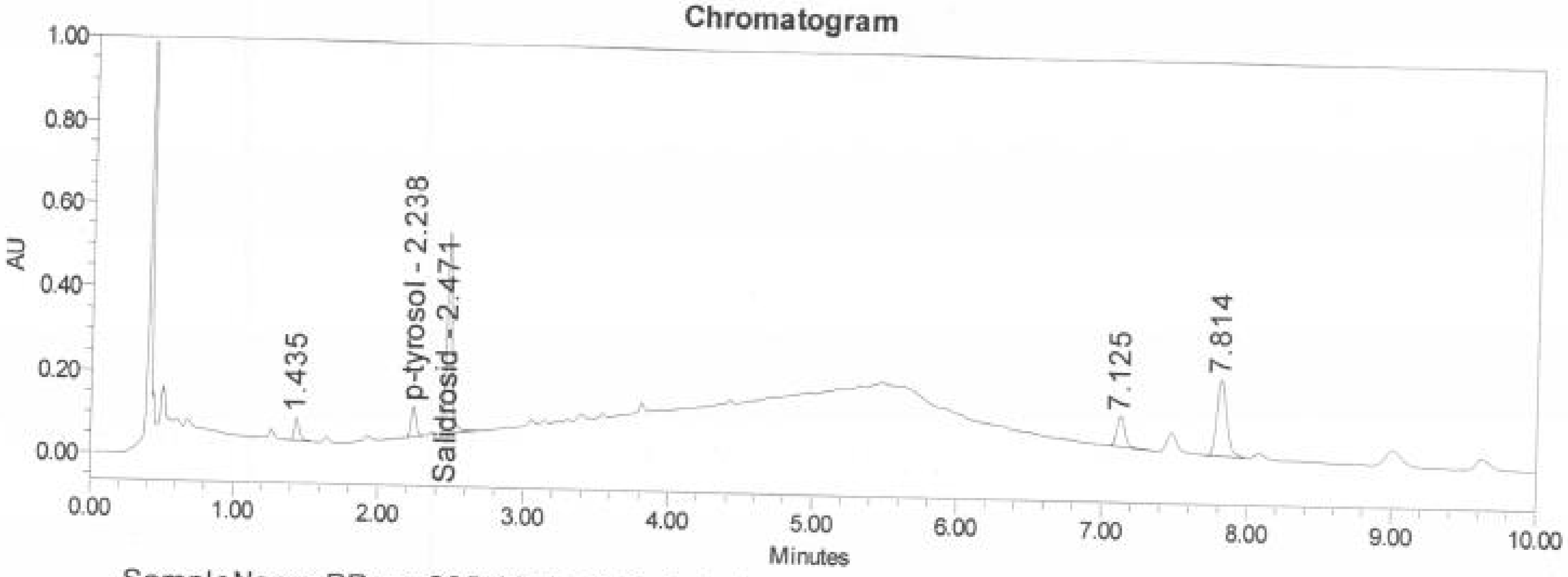

SampleName RR scc 325; Vial 1:A,3; Date Acquired 2016-11-23 11:29:33 CET; Channel Name 221 nm

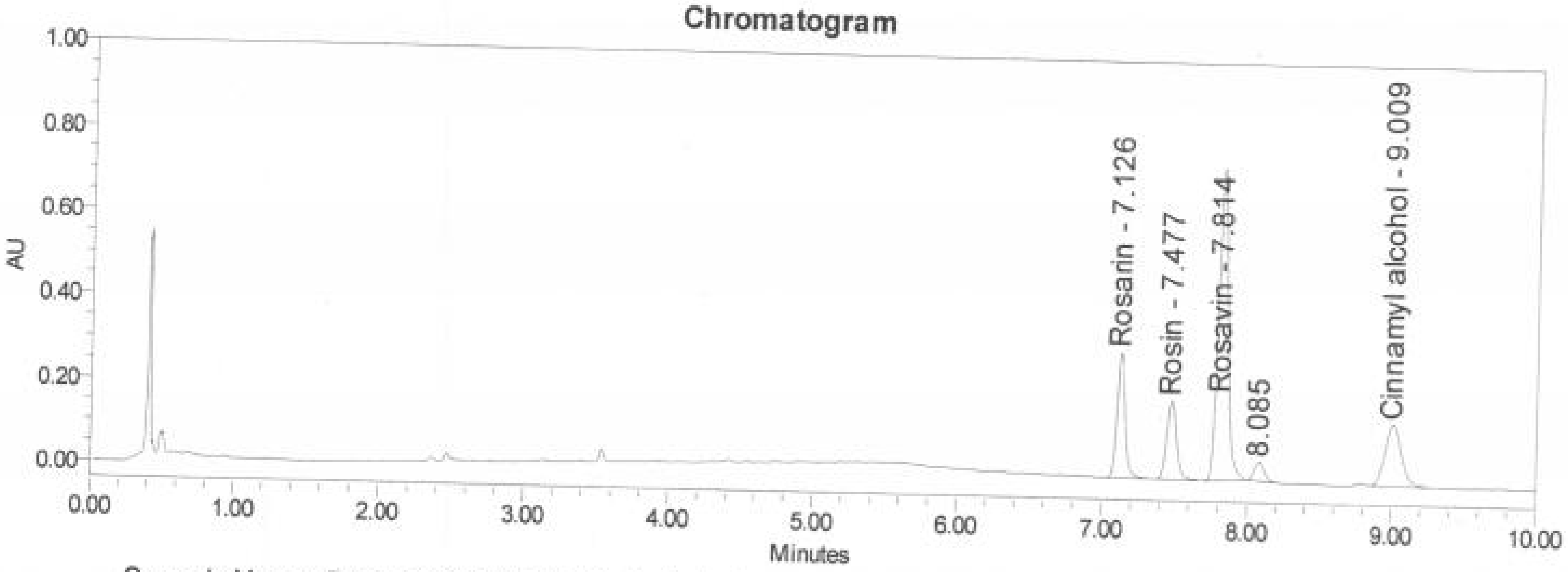

SampleName RR scc 325; Vial 1:A,3; Date Acquired 2016-11-23 11:29:33 CET; Channel Name 252 nm

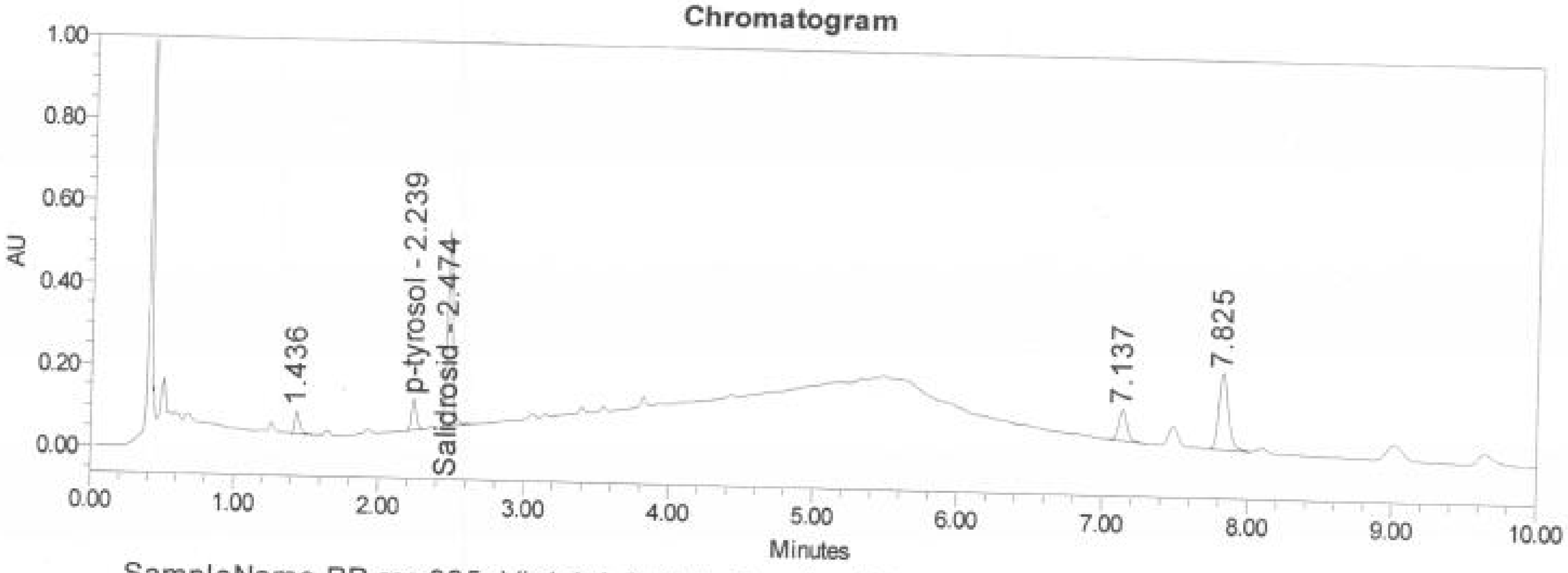

SampleName RR scc 325; Vial 1:A,3; Date Acquired 2016-11-23 11:56:34 CET; Channel Name 221 nm

Sign.

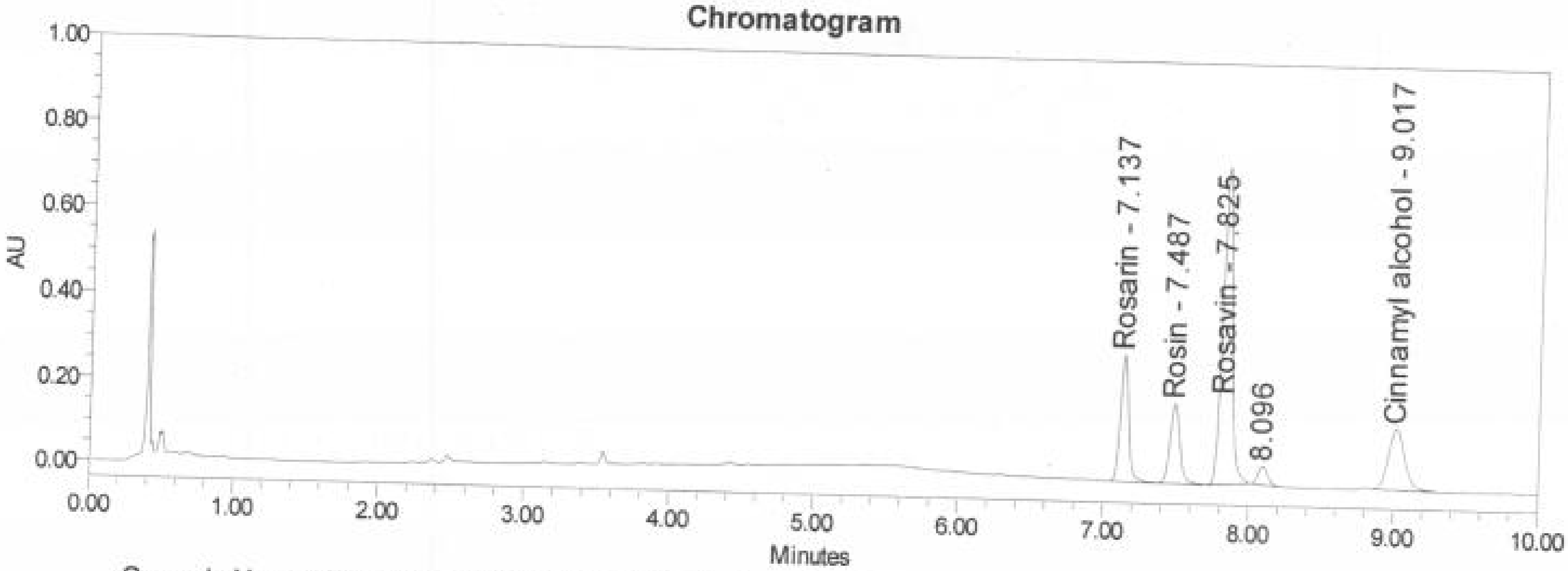

SampleName RR scc 325; Vial 1:A,3; Date Acquired 2016-11-23 11:56:34 CET; Channel Name 252 nm

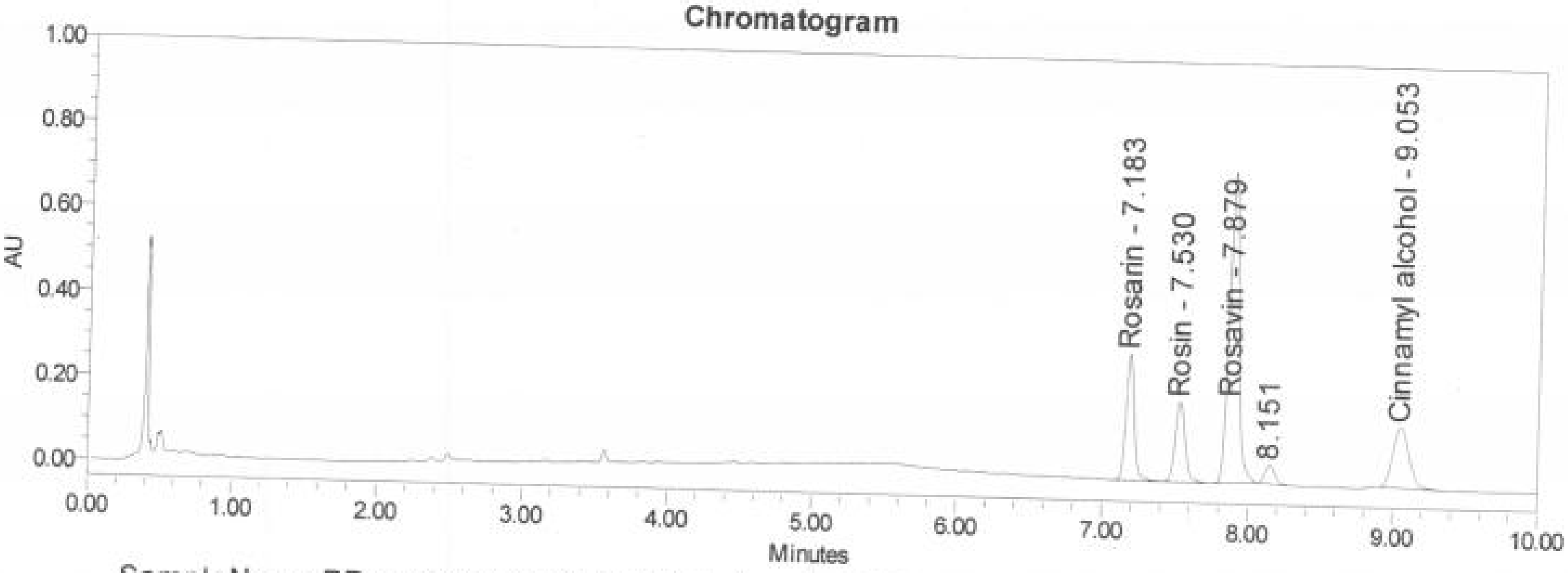

SampleName RR scc 325; Vial 1:A,3; Date Acquired 2016-11-23 12:23:32 CET; Channel Name 252 nm

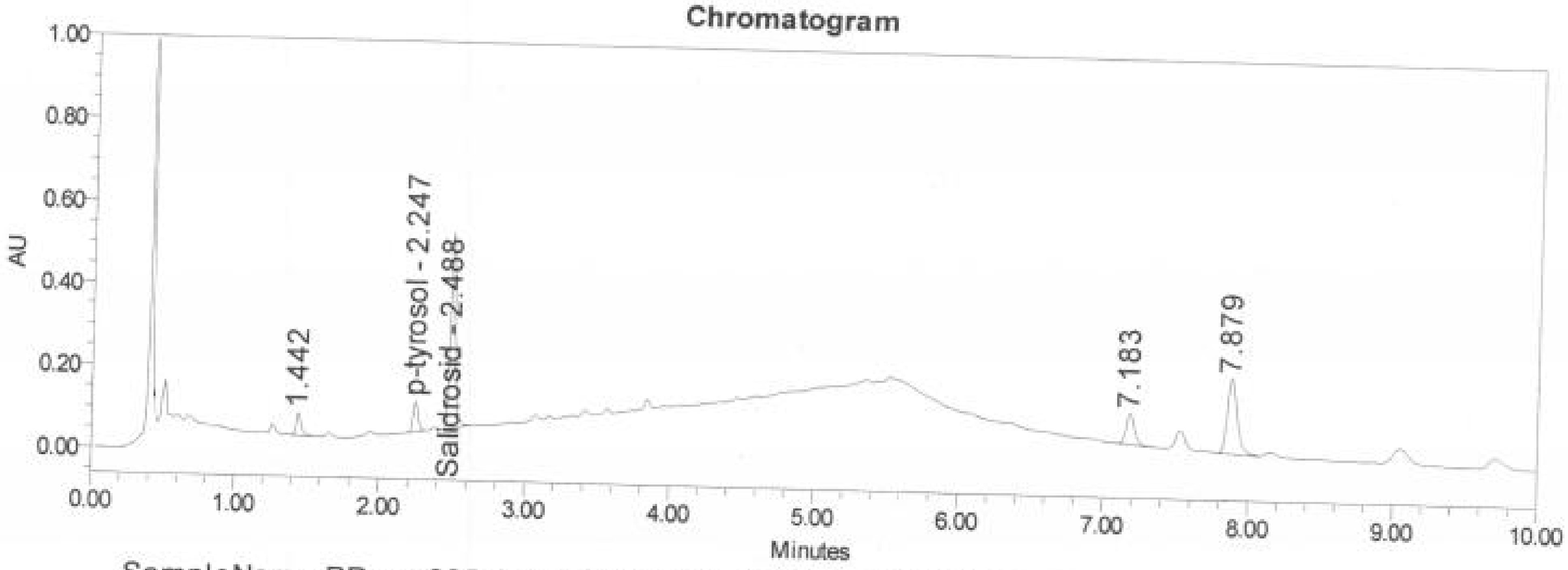

SampleName RR scc 325; Vial 1:A,3; Date Acquired 2016-11-23 12:23:32 CET; Channel Name 221 nm

Sign
