# Supplementary material for: Assessing the Quality and Potential Efficacy of Commercial Extracts of Rhodiola rosea L. by Analyzing the Salidroside and Rosavin Content and the Electrophysiological Activity in Hippocampal Long-Term Potentiation, a Synaptic Model of Memory
Source: Front Pharmacol. 2018 May 24;9:425. doi: 10.3389/fphar.2018.00425 (PMC5976749; doi:10.3389/fphar.2018.00425)
Supplement: Supplementary file 3 [file Data_Sheet_3.pdf]

Supplementary data S3:

Appendix to Figure 4. Statistical analysis

Conc.	Rosavin	Rosavin	Rosavin	Salidroside	Salidroside	Salidroside
	Mean	SD	n	Mean	SD	n
0.00	1118.27	106.00	10	1118.27	106.00	10
0.25	1200.938	39.35542	4	1246.50	31.43909	4
0.50	1701.688	125.435	4	1564.25	132.4152	4
0.75	2000.50	199.7579	4	2050.875	66.85759	4
1.50	2145.50	165.7442	4	1920.375	73.6256	4

Efficacy outcome measure: SS
Statistical tool: Two-way ANOVA

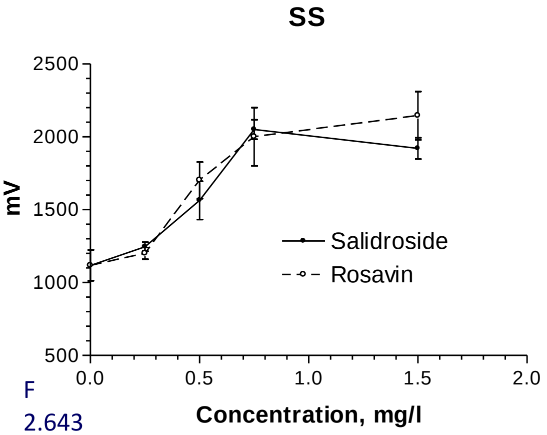

Source of Variation	% of total variation	P value
Treatment	0.43	0.1209
Concentration	90.49	P<0.0001

Source of Variation	P value summary	Significant?
Treatment	ns	No
Concentration	***	Yes

Source of Variation	Df	Sum-of-squares	Mean square	F
Interaction	4	136300	34070	2.643
Treatment	1	32310	32310	2.506
Concentration	4	67560001	689000	131.0
Residual	42	541500	12890	

Number of missing values -10

Bonferroni posttests

Concentration	Rosavin	Salidroside	Difference	95% CI of diff.
0.0000	1118	1118	0.0000	-137.0 to 137.0
0.2500	1201	1247	45.56	-171.1 to 262.2
0.5000	1702	1564	137.4	-354.1 to 79.18
0.7500	2001	2051	50.38	-166.2 to 267.0
1.500	2146	1920	225.1	-441.7 to -8.505

Concentration	Difference	t	P value	Summary
0.0000	0.0000	0.0000	P>0.05	ns
0.2500	45.56	0.5675	P>0.05	ns
0.5000	137.4	1.712	P>0.05	ns
0.7500	50.38	0.6274	P>0.05	ns
1.500	225.1	2.804	P<0.05	*

Appendix to Figure 4. Statistical analysis

Conc	Rosavin	Rosavin	Rosavin	Salidroside	Salidroside	Salidroside
	Mean	SD	n	Mean	SD	n
0.00	2266.00	187.00	10	2266.00	187.00	10
0.25	2258.00	282.00	4	2271.00	144.00	4
0.50	2861.00	99.00	4	3218.00	183.00	4
0.75	4210.00	191.00	4	4003.00	134.00	4
1.50	4258.00	214.00	4	4091.00	134.00	4

Efficacy outcome measure: TBS
Concentration of the extracts: 5 mg/ml
Statistical tool: One-way analysis of variance

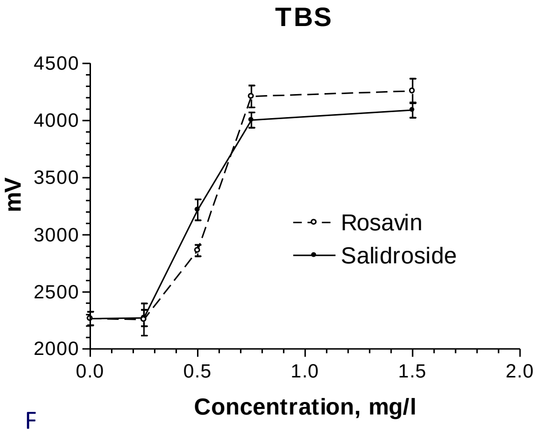

Efficacy outcome measure: TBS
Statistical tool: Two-way ANOVA

Source of Variation	% of total variation	P value
Treatment	0.00	0.9883
Concentration	94.52	P<0.0001

Source of Variation	P value summary	Significant?
Treatment	ns	No
Concentration	***	Yes

Source of Variation	Df	Sum-of-squares	Mean square	F
Interaction	4	450800	112700	3.346
Treatment	1	7.273	7.273	0.0002159
Concentration	4	32180000	8046000	238.9
Residual	42	1415000	33680	

Number of missing values -10

Bonferroni posttests

Concentration	Rosavin	Salidroside	Difference	95% CI of diff.
0.0000	2266	2266	0.0000	-221.4 to 221.4
0.2500	2258	2271	13.00	-337.1 to 363.1
0.5000	2861	3218	357.0	6.859 to 707.1
0.7500	4210	4003	207.0	-557.1 to 143.1
1.500	4258	4091	167.0	-517.1 to 183.1

Concentration	Difference	t	P value	Summary
0.0000	0.0000	0.0000	P>0.05	ns
0.2500	13.00	0.1002	P>0.05	ns
0.5000	357.0	2.751	P<0.05	*
0.7500	207.0	1.595	P>0.05	ns
1.500	167.0	1.287	P>0.05	ns

Appendix to Figure 5. Statistical analysis

Efficacy outcome measure: TBS
Concentration of the extracts: 5 mg/ml
Statistical tool: One-way analysis of variance

P value P<0.0001	
P value summary	***
Are means signif. different? (P < 0.05)	Yes
Number of groups	8
F	12.53
R squared	0.7451

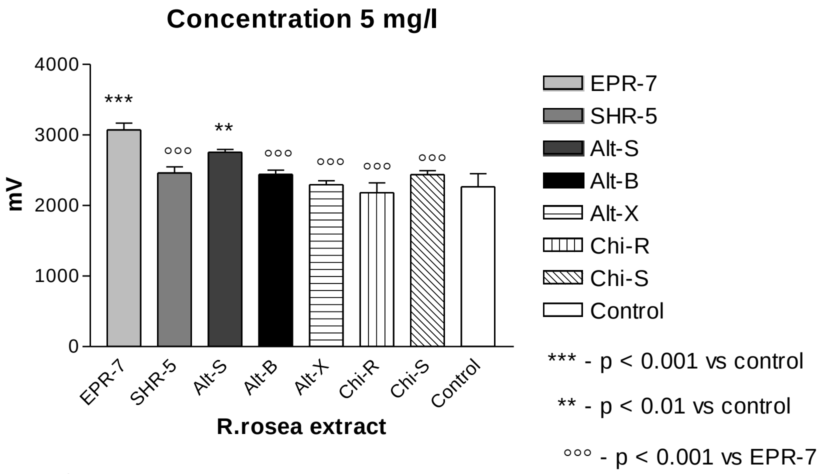

ANOVA Table	SS	df	MS
Treatment (between columns)	2636000	7	376500
Residual (within columns)	901500	30	30050
Total 353700037			

Tukey's Multiple Comparison Test	Mean Diff.	q	P value	95% CI of diff
**Control vs EPR-7**	**-804.9**	**11.10**	**P < 0.001**	**-1139 to -471.2**
Control vs SHR-5	-195.8	2.700	P > 0.05	-529.6 to 137.9
**Control vs Alt-S**	**-488.0**	**6.729**	**P < 0.01**	**-821.7 to -154.3**
Control vs Alt-B	-174.0	2.399	P > 0.05	-507.7 to 159.7
Control vs Alt-X	-30.00	0.4137	P > 0.05	-363.7 to 303.7
Control vs Chi-R	85.00	1.172	P > 0.05	-248.7 to 418.7
Control vs Chi-S	-172.0	2.372	P > 0.05	-505.7 to 161.7
**EPR-7 vs SHR-5**	**609.1**	**7.027**	**P < 0.001**	**210.2 to 1008**
EPR-7 vs Alt-S	316.9	3.656	P > 0.05	-81.95 to 715.8
**EPR-7 vs Alt-B**	**630.9**	**7.279**	**P < 0.001**	**232.0 to 1030**
**EPR-7 vs Alt-X**	**774.9**	**8.941**	**P < 0.001**	**376.0 to 1174**
**EPR-7 vs Chi-R**	**889.9**	**10.27**	**P < 0.001**	**491.0 to 1289**
**EPR-7 vs Chi-S**	**632.9**	**7.302**	**P < 0.001**	**234.0 to 1032**
SHR-5 vs Alt-S	-292.2	3.371	P > 0.05	-691.0 to 106.7
SHR-5 vs Alt-B	21.83	0.2519	P > 0.05	-377.0 to 420.7
SHR-5 vs Alt-X	165.8	1.913	P > 0.05	-233.0 to 564.7
SHR-5 vs Chi-R	280.8	3.240	P > 0.05	-118.0 to 679.7
SHR-5 vs Chi-S	23.83	0.2749	P > 0.05	-375.0 to 422.7
Alt-S vs Alt-B	314.0	3.623	P > 0.05	-84.87 to 712.9
**Alt-S vs Alt-X**	**458.0**	**5.284**	**P < 0.05**	**59.13 to 856.9**
**Alt-S vs Chi-R**	**573.0**	**6.611**	**P < 0.01**	**174.1 to 971.9**
Alt-S vs Chi-S	316.0	3.646	P > 0.05	-82.87 to 714.9
Alt-B vs Alt-X	144.0	1.661	P > 0.05	-254.9 to 542.9
Alt-B vs Chi-R	259.0	2.988	P > 0.05	-139.9 to 657.9
Alt-B vs Chi-S	2.000	0.02307	P > 0.05	-396.9 to 400.9
Alt-X vs Chi-R	115.0	1.327	P > 0.05	-283.9 to 513.9
Alt-X vs Chi-S	-142.0	1.638	P > 0.05	-540.9 to 256.9
Chi-R vs Chi-S	-257.0	2.965	P > 0.05	-655.9 to 141.9

Appendix to Figure 6. Statistical analysis

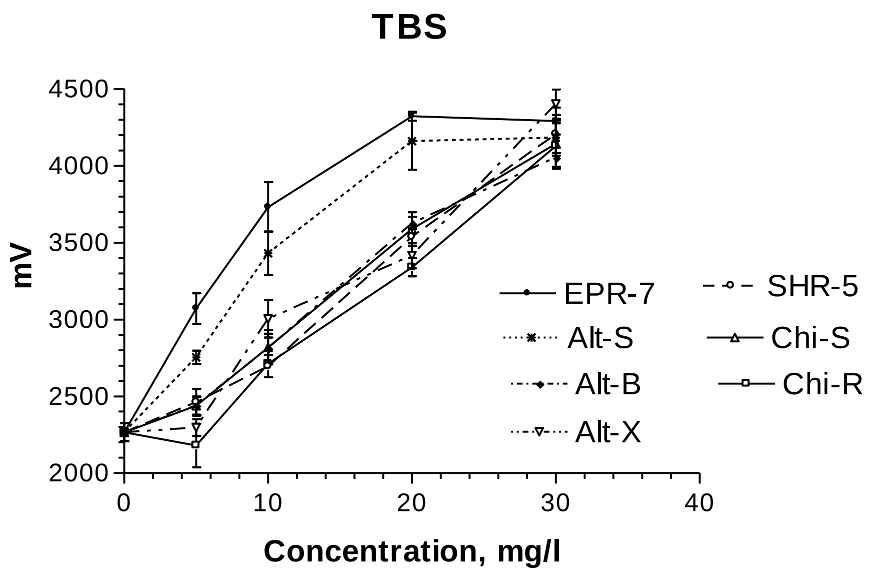

Two-way ANOVA

Source of Variation	% of total variation		P value	
Drug	6.46			
P<0.0001				
Concentration	83.22		P<0.0001	
Source of Variation	P value summary		Significant?	
Treatment	***		Yes	
Concentration	***		Yes	
Source of Variation	Df	Sum-of-squares	Mean square	F
Interaction	24	4581000	190900	4.902
Drug	6	6454000	1076000	27.63
Concentration	4	83140000	20790000	533.8
Residual	147	5724000	38940	

Appendix to Figure 6. Statistical analysis

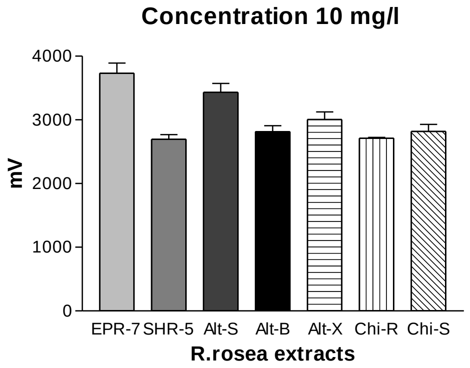

Efficacy outcome measure: TBS
Concentration of the extracts: 10 mg/ml
Statistical tool: One-way analysis of variance

P value	P<0.0001
P value summary	***
Are means signif. different? (P < 0.05)	Yes
Number of groups	7
F	12.74
R squared	0.7845

ANOVA Table	SS	df	MS
Treatment (between columns)	38280006		638000
Residual (within columns)	105100021		50070
Total	487900027		

Tukey's Multiple Comparison Test	Mean Diff.	q	P value	95% CI of diff
EPR-7 vs SHR-5	1036	9.260	P < 0.001	521.3 to 1551
EPR-7 vs Alt-S	301.0	2.690	P > 0.05	-213.7 to 815.7
EPR-7 vs Alt-B	917.0	8.196	P < 0.001	402.3 to 1432
EPR-7 vs Alt-X	727.0	6.498	P < 0.01	212.3 to 1242
EPR-7 vs Chi-R	1019	9.108	P < 0.001	504.3 to 1534
EPR-7 vs Chi-S	913.0	8.161	P < 0.001	398.3 to 1428
SHR-5 vs Alt-S	-735.0	6.570	P < 0.01	-1250 to -220.3
SHR-5 vs Alt-B	-119.0	1.064	P > 0.05	-633.7 to 395.7
SHR-5 vs Alt-X	-309.0	2.762	P > 0.05	-823.7 to 205.7
SHR-5 vs Chi-R	-17.00	0.1520	P > 0.05	-531.7 to 497.7
SHR-5 vs Chi-S	-123.0	1.099	P > 0.05	-637.7 to 391.7
Alt-S vs Alt-B	616.0	5.506	P < 0.05	101.3 to 1131
Alt-S vs Alt-X	426.0	3.808	P > 0.05	-88.67 to 940.7
Alt-S vs Chi-R	718.0	6.418	P < 0.01	203.3 to 1233
Alt-S vs Chi-S	612.0	5.470	P < 0.05	97.33 to 1127
Alt-B vs Alt-X	-190.0	1.698	P > 0.05	-704.7 to 324.7
Alt-B vs Chi-R	102.0	0.9117	P > 0.05	-412.7 to 616.7
Alt-B vs Chi-S	-4.000	0.03575	P > 0.05	-518.7 to 510.7
Alt-X vs Chi-R	292.0	2.610	P > 0.05	-222.7 to 806.7
Alt-X vs Chi-S	186.0	1.663	P > 0.05	-328.7 to 700.7
Chi-R vs Chi-S	-106.0	0.9475	P > 0.05	-620.7 to 408.8

Appendix to Figure 6. Statistical analysis

Efficacy outcome measure: TBS
Concentration of the extracts: 20 mg/ml
Statistical tool: One-way analysis of variance

P value	P<0.0001
P value summary	***
Are means signif. different? (P < 0.05)	Yes
Number of groups	7
F	15.89
R squared	0.8195

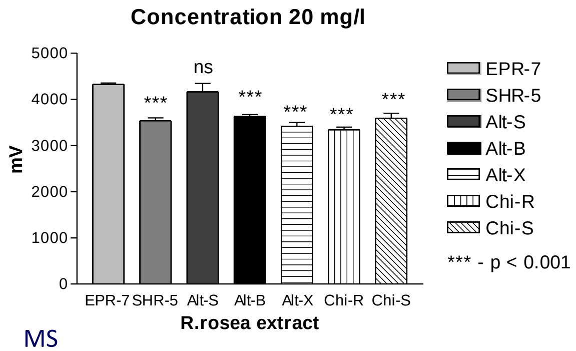

ANOVA Table	SS	df	MS
Treatment (between columns)	3408000	6	568000
Residual (within columns)	750600	21	35740
Total	4159000	27	

Tukey's Multiple Comparison Test	Mean Diff.	q	P value	95% CI of diff
**EPR-7 vs SHR-5**	**786.0**	**8.315**	**P < 0.001**	**351.1 to 1221**
EPR-7 vs Alt-S	163.0	1.724	P > 0.05	-271.9 to 597.9
**EPR-7 vs Alt-B**	**695.0**	**7.352**	**P < 0.001**	**260.1 to 1130**
**EPR-7 vs Alt-X**	**906.0**	**9.584**	**P < 0.001**	**471.1 to 1341**
**EPR-7 vs Chi-R**	**983.0**	**10.40**	**P < 0.001**	**548.1 to 1418**
**EPR-7 vs Chi-S**	**733.0**	**7.754**	**P < 0.001**	**298.1 to 1168**
**SHR-5 vs Alt-S**	**-623.0**	**6.591**	**P < 0.01**	**-1058 to -188.1**
SHR-5 vs Alt-B	-91.00	0.9627	P > 0.05	-525.9 to 343.9
SHR-5 vs Alt-X	120.0	1.269	P > 0.05	-314.9 to 554.9
SHR-5 vs Chi-R	197.0	2.084	P > 0.05	-237.9 to 631.9
SHR-5 vs Chi-S	-53.00	0.5607	P > 0.05	-487.9 to 381.9
**Alt-S vs Alt-B**	**532.0**	**5.628**	**P < 0.05**	**97.15 to 966.9**
**Alt-S vs Alt-X**	**743.0**	**7.860**	**P < 0.001**	**308.1 to 1178**
**Alt-S vs Chi-R**	**820.0**	**8.675**	**P < 0.001**	**385.1 to 1255**
**Alt-S vs Chi-S**	**570.0**	**6.030**	**P < 0.01**	**135.1 to 1005**
Alt-B vs Alt-X	211.0	2.232	P > 0.05	-223.9 to 645.9
Alt-B vs Chi-R	288.0	3.047	P > 0.05	-146.9 to 722.9
Alt-B vs Chi-S	38.00	0.4020	P > 0.05	-396.9 to 472.9
Alt-X vs Chi-R	77.00	0.8146	P > 0.05	-357.9 to 511.9
Alt-X vs Chi-S	-173.0	1.830	P > 0.05	-607.9 to 261.9
Chi-R vs Chi-S	-250.0	2.645	P > 0.05	-684.9 to 184.9
