# Supplementary material for: Assessing the Quality and Potential Efficacy of Commercial Extracts of Rhodiola rosea L. by Analyzing the Salidroside and Rosavin Content and the Electrophysiological Activity in Hippocampal Long-Term Potentiation, a Synaptic Model of Memory
Source: Front Pharmacol. 2018 May 24;9:425. doi: 10.3389/fphar.2018.00425 (PMC5976749; doi:10.3389/fphar.2018.00425)
Supplement: Supplementary file 4 [file Data_Sheet_4.pdf]

9 Appendix

Control															
**slice**	**ACSF +SS**					**ACSF +SS**					**ACSF +TBS**				
**time [min]**	**10**	**20**	**30**	**40**	**Mean 20-40**	**50**	**60**	**70**	**80**	**Mean 60-80**	**90**	**100**	**110**	**120**	**Mean 100-120**
**1**	-1105,00	-1113,00	-1080,00	-899,35	**-1030,78**	-1013,00	-1092,00	-855,45	-1264,00	**-1070,48**	-2235,00	-2699,00	-2359,00	-2689,00	**-2582,33**
**2**	-981,85	-1102,00	-1142,00	-1113,00	**-1119,00**	-1286,00	-1233,00	-1316,00	-1232,00	**-1260,33**	-2719,00	-2565,00	-2567,00	-2492,00	**-2541,33**
**3**	-549,34	-1365,00	-1179,00	-1133,00	**-1225,67**	-1092,00	-1234,00	-975,13	-1288,00	**-1165,71**	-2347,00	-2248,00	-2278,00	-2293,00	**-2273,00**
**4**	-1041,00	-1216,00	-1050,00	-1063,00	**-1109,67**	-1232,00	-1175,00	-1069,00	-943,51	**-1062,50**	-2137,00	-2035,00	-2209,00	-2164,00	**-2136,00**
**5**	-1059,00	-1091,00	-937,05	-946,53	**-991,53**	-934,76	-945,95	-903,57	-827,57	**-892,36**	-2250,00	-1933,00	-2163,00	-2073,00	**-2056,33**
**6**	-851,44	-940,91	-1008,00	-1113,00	**-1020,64**	-1169,00	-1038,00	-1098,00	-1037,00	**-1057,67**	-2098,00	-2051,00	-2086,00	-2349,00	**-2162,00**
**7**	-717,92	-867,28	-1124,00	-896,54	**-962,61**	-1248,00	-1056,00	-1061,00	-1061,00	**-1059,33**	-1466,00	-2162,00	-2122,00	-2088,00	**-2124,00**
**8**	-1298,00	-1072,00	-1058,00	-1326,00	**-1152,00**	-1045,00	-1117,00	-1280,00	-1097,00	**-1164,67**	-2230,00	-2184,00	-2245,00	-2124,00	**-2184,33**
**9**	-840,06	-908,15	-1033,00	-1214,00	**-1051,72**	-1257,00	-1252,00	-1214,00	-1168,00	**-1211,33**	-2622,00	-2422,00	-2074,00	-1994,00	**-2163,33**
**10**	-893,26	-1099,00	-939,95	-961,62	**-1000,19**	-946,03	-1009,00	-898,36	-1210,00	**-1039,12**	-2151,00	-1896,00	-2166,00	-2173,00	**-2078,33**
**11**	-1551,00	-1347,00	-1074,00	-1120,00	**-1180,33**	-1255,00	-1377,00	-1143,00	-1224,00	**-1248,00**	-2837,00	-2336,00	-2523,00	-2553,00	**-2470,67**
**12**	-1218,00	-1242,00	-1057,00	-1297,00	**-1198,67**	-1232,00	-1237,00	-1136,00	-1190,00	**-1187,67**	-2198,00	-2203,00	-2529,00	-2542,00	**-2424,67**
**Mean**	-1008,82	-1113,61	-1056,83	-1090,25	**-1086,90**	-1142,48	-1147,16	-1079,13	-1128,51	**-1118,27**	-2274,17	-2227,83	-2276,75	-2294,50	**-2266,36**
**SD**	269,21	159,53	72,95	144,38	**88,99**	129,61	124,80	149,88	139,58	**106,49**	352,78	244,55	177,74	227,35	**187,63**
**SEM**	77,81	46,11	21,08	41,73	25,72	37,46	36,07	43,32	40,34	30,78	101,96	70,68	51,37	65,71	54,23

Tab. 2 Effects of Placebo on pyramidal cell activity in terms of changes of population spike amplitudes. Results from single slices as obtained after single stimuli (SS) or after burst stimuli (TBS). Overview on final results after averaging 12 slices. SD=standard deviation; SEM=standard error of mean. ACSF=artificial cerebro-spinal fluid.

NCAG 1517															
**slice**	**ACSF +SS**					**RR-EUR-S 5.00 mg/l + SS**					**RR-EUR-S 5.00 mg/l + TBS**				
**time [min]**	**10**	**20**	**30**	**40**	**Mean 20-40**	**50**	**60**	**70**	**80**	**Mean 60-80**	**90**	**100**	**110**	**120**	**Mean 100-120**
**1**	-993,96	-1138,00	-872,96	-1025,00	**-1011,99**	-1138,00	-1430,00	-1577,00	-1882,00	**-1629,67**	-2690,00	-2709,00	-3018,00	-3332,00	**-3019,67**
**2**	-582,41	-993,62	-1065,00	-1091,00	**-1049,87**	-1267,00	-1432,00	-1505,00	-1407,00	**-1448,00**	-2726,00	-3142,00	-3141,00	-2967,00	**-3083,33**
**3**	-1150,00	-1036,00	-960,99	-992,00	**-996,33**	-1047,00	-1214,00	-1326,00	-1293,00	**-1277,67**	-2154,00	-2911,00	-2529,00	-3117,00	**-2852,33**
**4**	-1207,00	-1123,00	-1109,00	-1129,00	**-1120,33**	-1302,00	-1703,00	-1672,00	-1681,00	**-1685,33**	-3268,00	-3467,00	-3365,00	-3153,00	**-3328,33**
**Mean**	-983,34	-1072,66	-1001,99	-1059,25	**-1044,63**	-1188,50	-1444,75	-1520,00	-1565,75	**-1510,17**	-2709,50	-3057,25	-3013,25	-3142,25	**-3070,92**
**SD**	282,05	69,27	106,07	62,10	**55,25**	117,78	200,27	146,30	266,39	**185,19**	455,03	325,45	353,35	149,97	**197,33**
**SEM**	141,03	34,63	53,03	31,05	27,62	58,89	100,13	73,15	133,19	92,59	227,51	162,72	176,68	74,98	98,67
**P<**	n.s.	n.s.	n.s.	n.s.	n.s.	n.s.	0.02	0.01	0.01	0.01	n.s.	0.01	0.01	0.01	0.01

Tab. 3 Effects of RR-Eur-S 5.00 mg/l on pyramidal cell activity in terms of changes of population spike amplitudes. Results from single slices as obtained after single stimuli (SS) or after burst stimuli (TBS). Overview on final results after averaging 4 slices. SD=standard deviation; SEM=standard error of mean. P<=Wilcoxon Mann Whitny U-Test. ACSF=artificial cerebro-spinal fluid.

Report NCAG 15/17 H – Hippocampus slice preparation

NCAG 1517															
slice	ACSF +SS					RR-EUR-S 10.0 mg/l + SS					RR-EUR-S 10.0 mg/l + TBS				
time [min]	10	20	30	40	Mean 20-40	50	60	70	80	Mean 60-80	90	100	110	120	Mean 100-120
1	-1283,00	-944,85	-987,44	-1040,00	**-990,76**	-1556,00	-1609,00	-1557,00	-1764,00	**-1643,33**	-3243,00	-4114,00	-4064,00	-4399,00	**-4192,33**
2	-1097,00	-981,47	-983,71	-975,02	**-980,07**	-1551,00	-1691,00	-1814,00	-1783,00	**-1762,67**	-4050,00	-3841,00	-3930,00	-3384,00	**-3718,33**
3	-961,01	-1215,00	-1093,00	-1080,00	**-1129,33**	-1319,00	-1912,00	-2054,00	-2034,00	**-2000,00**	-3450,00	-3378,00	-3629,00	-3568,00	**-3525,00**
4	-916,41	-1082,00	-951,88	-938,57	**-990,82**	-976,58	-1881,00	-1810,00	-1895,00	**-1862,00**	-3392,00	-3433,00	-3712,00	-3328,00	**-3491,00**
Mean	-1064,36	-1055,83	-1004,01	-1008,40	**-1022,75**	-1350,65	-1773,25	-1808,75	-1869,00	**-1817,00**	-3533,75	-3691,50	-3833,75	-3669,75	**-3731,67**
SD	164,76	120,92	61,44	63,55	**71,24**	272,79	146,75	202,94	124,26	**151,25**	355,04	349,27	199,19	496,86	**323,02**
SEM	82,38	60,46	30,72	31,77	35,62	136,39	73,37	101,47	62,13	75,62	177,52	174,63	99,59	248,43	161,51
P<	n.s.	n.s.	n.s.	n.s.	n.s.	0.01	0.01	0.01	0.01	0.01	0.01	0.01	0.01	0.01	0.01

Tab. 4 Effects of RR-Eur-S 10.0 mg/l on pyramidal cell activity in terms of changes of population spike amplitudes. Results from single slices as obtained after single stimuli (SS) or after burst stimuli (TBS). Overview on final results after averaging 4 slices. SD=standard deviation; SEM=standard error of mean. P<=Wilcoxon Mann Whitny U-Test. ACSF=artificial cerebro-spinal fluid.

NCAG 1517															
slice	ACSF +SS					RR-EUR-S 20.0 mg/l + SS					RR-EUR-S 20.0 mg/l + TBS				
time [min]	10	20	30	40	Mean 20-40	50	60	70	80	Mean 60-80	90	100	110	120	Mean 100-120
1	-1080,00	-1107,00	-1052,00	-1075,00	**-1078,00**	-2416,00	-2095,00	-2015,00	-1953,00	**-2021,00**	-4582,00	-4889,00	-3868,00	-4119,00	**-4292,00**
2	-1000,00	-1297,00	-1089,00	-1067,00	**-1151,00**	-2051,00	-1963,00	-2116,00	-2017,00	**-2032,00**	-4635,00	-4448,00	-4463,00	-4321,00	**-4410,67**
3	-927,26	-940,89	-1033,00	-1048,00	**-1007,30**	-1947,00	-1840,00	-2069,00	-1875,00	**-1928,00**	-3261,00	-4285,00	-4127,00	-4478,00	**-4296,67**
4	-831,72	-1293,00	-1001,00	-851,34	**-1048,45**	-2186,00	-2221,00	-1960,00	-1962,00	**-2047,67**	-4123,00	-4751,00	-3983,00	-4140,00	**-4291,33**
Mean	-959,75	-1159,47	-1043,75	-1010,34	**-1071,19**	-2150,00	-2029,75	-2040,00	-1951,75	**-2007,17**	-4150,25	-4593,25	-4110,25	-4264,50	**-4322,67**
SD	105,72	170,56	36,78	106,60	**60,60**	202,54	164,62	67,43	58,47	**53,90**	635,85	275,96	257,93	168,77	**58,71**
SEM	52,86	85,28	18,39	53,30	30,30	101,27	82,31	33,72	29,23	26,95	317,92	137,98	128,97	84,38	29,36
P<	n.s.	n.s.	n.s.	n.s.	n.s.	0.01	0.01	0.01	0.01	0.01	0.01	0.01	0.01	0.01	0.01

Tab. 5 Effects of RR-Eur-S 20.0 mg/l on pyramidal cell activity in terms of changes of population spike amplitudes. Results from single slices as obtained after single stimuli (SS) or after burst stimuli (TBS). Overview on final results after averaging 4 slices. SD=standard deviation; SEM=standard error of mean. P<=Wilcoxon Mann Whitny U-Test. ACSF=artificial cerebro-spinal fluid.

Report NCAG 15/17 H – Hippocampus slice preparation

NCAG 1517															
**slice**	**ACSF +SS**					**RR-EUR-S 30.0 mg/l + SS**					**RR-EUR-S 30.0 mg/l + TBS**				
**time [min]**	**10**	**20**	**30**	**40**	**Mean 20-40**	**50**	**60**	**70**	**80**	**Mean 60-80**	**90**	**100**	**110**	**120**	**Mean 100-120**
**1**	-955,10	-971,79	-1036,00	-1052,00	**-1019,93**	-1636,00	-2014,00	-2079,00	-2111,00	**-2068,00**	-4031,00	-4417,00	-4567,00	-4576,00	**-4520,00**
**2**	-941,41	-990,72	-1016,00	-939,85	**-982,19**	-1845,00	-1999,00	-2151,00	-2119,00	**-2089,67**	-3720,00	-4210,00	-4247,00	-4251,00	**-4236,00**
**3**	-863,39	-1029,00	-1120,00	-978,62	**-1042,54**	-1778,00	-2041,00	-2194,00	-2297,00	**-2177,33**	-3981,00	-4601,00	-3739,00	-3958,00	**-4099,33**
**4**	-904,73	-1200,00	-1039,00	-1001,00	**-1080,00**	-2512,00	-2216,00	-2060,00	-2351,00	**-2209,00**	-3148,00	-4380,00	-4226,00	-4326,00	**-4310,67**
**Mean**	-916,16	-1047,88	-1052,75	-992,87	**-1031,17**	-1942,75	-2067,50	-2121,00	-2219,50	**-2136,00**	-3720,00	-4402,00	-4194,75	-4277,75	**-4291,50**
**SD**	41,11	104,17	45,98	46,82	**40,98**	389,38	100,51	62,49	122,71	**67,84**	404,98	160,39	341,56	254,45	**175,68**
**SEM**	20,55	52,08	22,99	23,41	20,49	194,69	50,26	31,24	61,35	33,92	202,49	80,19	170,78	127,23	87,84
**P<**	n.s.	n.s.	n.s.	n.s.	n.s.	0.01	0.01	0.01	0.01	0.01	0.01	0.01	0.01	0.01	0.01

Tab. 6 Effects of RR-Eur-S 30.0 mg/l on pyramidal cell activity in terms of changes of population spike amplitudes. Results from single slices as obtained after single stimuli (SS) or after burst stimuli (TBS). Overview on final results after averaging 4 slices. SD=standard deviation; SEM=standard error of mean. P<=Wilcoxon Mann Whitny U-Test. ACSF=artificial cerebro-spinal fluid.

NCAG 1517															
**slice**	**ACSF +SS**					**RR-Chi-R 5.00 mg/l + SS**					**RR-Chi-R 5.00 mg/l + TBS**				
**time [min]**	**10**	**20**	**30**	**40**	**Mean 20-40**	**50**	**60**	**70**	**80**	**Mean 60-80**	**90**	**100**	**110**	**120**	**Mean 100-120**
**1**	-981,93	-1039,00	-985,49	-1263,00	**-1095,83**	-937,37	-1167,00	-1197,00	-1168,00	**-1177,33**	-1746,00	-2033,00	-2048,00	-2070,00	**-2050,33**
**2**	-1076,00	-1027,00	-1030,00	-1153,00	**-1070,00**	-1292,00	-1166,00	-1068,00	-1188,00	**-1140,67**	-1534,00	-1844,00	-2034,00	-1997,00	**-1958,33**
**3**	-1113,00	-968,66	-964,67	-1023,00	**-985,44**	-1047,00	-1144,00	-1158,00	-1121,00	**-1141,00**	-1991,00	-2185,00	-2083,00	-2093,00	**-2120,33**
**4**	-1032,00	-1162,00	-1122,00	-1200,00	**-1161,33**	-1177,00	-1277,00	-1314,00	-1248,00	**-1279,67**	-1837,00	-2596,00	-2537,00	-2650,00	**-2594,33**
**Mean**	-1050,73	-1049,17	-1025,54	-1159,75	**-1078,15**	-1113,34	-1188,50	-1184,25	-1181,25	**-1184,67**	-1777,00	-2164,50	-2175,50	-2202,50	**-2180,83**
**SD**	56,57	81,26	69,84	101,70	**72,79**	154,21	59,95	101,98	52,62	**65,63**	190,97	319,70	241,88	301,13	**283,54**
**SEM**	28,28	40,63	34,92	50,85	36,39	77,10	29,97	50,99	26,31	32,81	95,48	159,85	120,94	150,56	141,77
**P<**	n.s.	n.s.	n.s.	n.s.	n.s.	n.s.	n.s.	n.s.	n.s.	n.s.	0.10	n.s.	n.s.	n.s.	n.s.

Tab. 7 Effects of RR-Chi-R 5.00 mg/l on pyramidal cell activity in terms of changes of population spike amplitudes. Results from single slices as obtained after single stimuli (SS) or after burst stimuli (TBS). Overview on final results after averaging 4 slices. SD=standard deviation; SEM=standard error of mean. P<=Wilcoxon Mann Whitny U-Test. ACSF=artificial cerebro-spinal fluid.

Report NCAG 15/17 H – Hippocampus slice preparation

NCAG 1517															
**slice**	**ACSF +SS**					**RR-Chi-R 10.0 mg/l + SS**					**RR-Chi-R 10.0 mg/l + TBS**				
**time [min]**	**10**	**20**	**30**	**40**	**Mean 20-40**	**50**	**60**	**70**	**80**	**Mean 60-80**	**90**	**100**	**110**	**120**	**Mean 100-120**
**1**	-934,37	-964,00	-992,25	-1126,00	**-1027,42**	-1425,00	-1370,00	-1351,00	-1375,00	**-1365,33**	-2197,00	-2594,00	-2557,00	-2969,00	**-2706,67**
**2**	-798,04	-1246,00	-1051,00	-985,64	**-1094,21**	-1634,00	-1657,00	-1737,00	-1735,00	**-1709,67**	-3304,00	-2572,00	-2704,00	-2940,00	**-2738,67**
**3**	-890,93	-948,49	-1139,00	-1152,00	**-1079,83**	-957,18	-957,18	-1572,00	-1633,00	**-1387,39**	-2235,00	-2669,00	-2503,00	-2853,00	**-2675,00**
**4**	-918,78	-954,73	-1079,00	-1094,00	**-1042,58**	-1351,00	-1288,00	-1534,00	-1404,00	**-1408,67**	-2878,00	-2605,00	-2767,00	-2816,00	**-2729,33**
**Mean**	-885,53	-1028,31	-1065,31	-1089,41	**-1061,01**	-1341,80	-1318,05	-1548,50	-1536,75	**-1467,77**	-2653,50	-2610,00	-2632,75	-2894,50	**-2712,42**
**SD**	61,03	145,27	60,99	73,13	**31,22**	283,03	287,93	158,43	175,45	**162,24**	534,50	41,66	123,39	71,89	**28,33**
**SEM**	30,52	72,63	30,50	36,57	15,61	141,52	143,97	79,22	87,73	81,12	267,25	20,83	61,69	35,95	14,17
**P<**	n.s.	n.s.	n.s.	n.s.	n.s.	n.s.	n.s.	0.01	0.01	0.02	n.s.	0.02	0.02	0.01	0.01

Tab. 8 Effects of RR-Chi-R 10.0 mg/l on pyramidal cell activity in terms of changes of population spike amplitudes. Results from single slices as obtained after single stimuli (SS) or after burst stimuli (TBS). Overview on final results after averaging 4 slices. SD=standard deviation; SEM=standard error of mean. P<=Wilcoxon Mann Whitny U-Test. ACSF=artificial cerebro-spinal fluid.

NCAG 1517															
**slice**	**ACSF +SS**					**RR-Chi-R 20.0 mg/kg + SS**					**RR-Chi-R 20.0 mg/kg + TBS**				
**time [min]**	**10**	**20**	**30**	**40**	**Mean 20-40**	**50**	**60**	**70**	**80**	**Mean 60-80**	**90**	**100**	**110**	**120**	**Mean 100-120**
**1**	-1063,00	-929,31	-1086,00	-1092,00	**-1035,77**	-1483,00	-1855,00	-2034,00	-2095,00	**-1994,67**	-3630,00	-3642,00	-3451,00	-3414,00	**-3502,33**
**2**	-1019,00	-1151,00	-1073,00	-1240,00	**-1154,67**	-1360,00	-1998,00	-1969,00	-1869,00	**-1945,33**	-3631,00	-3378,00	-3180,00	-3257,00	**-3271,67**
**3**	-910,34	-933,75	-1067,00	-931,79	**-977,51**	-1701,00	-2126,00	-2075,00	-2152,00	**-2117,67**	-3546,00	-3267,00	-3179,00	-3255,00	**-3233,67**
**4**	-814,94	-972,50	-1215,00	-1206,00	**-1131,17**	-1818,00	-1941,00	-2036,00	-1904,00	**-1960,33**	-3250,00	-3247,00	-3476,00	-3325,00	**-3349,33**
**Mean**	-951,82	-996,64	-1110,25	-1117,45	**-1074,78**	-1590,50	-1980,00	-2028,50	-2005,00	**-2004,50**	-3514,25	-3383,50	-3321,50	-3312,75	**-3339,25**
**SD**	111,55	104,72	70,28	139,02	**82,75**	207,09	113,70	43,93	139,53	**78,22**	180,61	181,71	164,29	74,93	**118,90**
**SEM**	55,78	52,36	35,14	69,51	41,38	103,54	56,85	21,96	69,77	39,11	90,31	90,86	82,14	37,47	59,45
**P<**	n.s.	n.s.	n.s.	n.s.	n.s.	0.01	0.01	0.01	0.01	0.01	0.01	0.01	0.01	0.01	0.01

Tab. 9 Effects of RR-Chi-R 20.0 mg/l on pyramidal cell activity in terms of changes of population spike amplitudes. Results from single slices as obtained after single stimuli (SS) or after burst stimuli (TBS). Overview on final results after averaging 4 slices. SD=standard deviation; SEM=standard error of mean. P<=Wilcoxon Mann Whitny U-Test. ACSF=artificial cerebro-spinal fluid.

Report NCAG 15/17 H – Hippocampus slice preparation

NCAG 1517															
**slice**	**ACSF +SS**					**RR-Chi-R 30.0 mg/kg + SS**					**RR-Chi-R 30.0 mg/kg + TBS**				
**time [min]**	**10**	**20**	**30**	**40**	**Mean 20-40**	**50**	**60**	**70**	**80**	**Mean 60-80**	**90**	**100**	**110**	**120**	**Mean 100-120**
**1**	-1005,00	-1023,00	-1081,00	-1031,00	**-1045,00**	-2248,00	-2644,00	-2508,00	-2366,00	**-2506,00**	-3224,00	-4444,00	-4254,00	-4874,00	**-4524,00**
**2**	-385,34	-937,85	-1008,00	-1054,00	**-999,95**	-1588,00	-1951,00	-1907,00	-2026,00	**-1961,33**	-4189,00	-4068,00	-4005,00	-4463,00	**-4178,67**
**3**	-627,23	-1074,00	-1089,00	-1003,00	**-1055,33**	-1699,00	-2368,00	-2437,00	-2444,00	**-2416,33**	-3383,00	-3779,00	-4201,00	-3809,00	**-3929,67**
**4**	-1002,00	-916,34	-1082,00	-1124,00	**-1040,78**	-2226,00	-2327,00	-2187,00	-2286,00	**-2266,67**	-3306,00	-3892,00	-3880,00	-3870,00	**-3880,67**
**Mean**	-754,89	-987,80	-1065,00	-1053,00	**-1035,27**	-1940,25	-2322,50	-2259,75	-2280,50	**-2287,58**	-3525,50	-4045,75	-4085,00	-4254,00	**-4128,25**
**SD**	303,58	73,65	38,17	51,72	**24,32**	345,76	284,88	272,50	181,51	**238,86**	447,07	290,91	173,63	507,79	**294,33**
**SEM**	151,79	36,82	19,08	25,86	12,16	172,88	142,44	136,25	90,76	119,43	223,54	145,46	86,81	253,90	147,17
**P<**	n.s.	n.s.	n.s.	n.s.	n.s.	0.01	0.01	0.01	0.01	0.01	0.01	0.01	0.01	0.01	0.01

Tab. 10 Effects of RR-RR-Chi-R 30.0 mg/l on pyramidal cell activity in terms of changes of population spike amplitudes. Results from single slices as obtained after single stimuli (SS) or after burst stimuli (TBS). Overview on final results after averaging 4 slices. SD=standard deviation; SEM=standard error of mean. P<=Wilcoxon Mann Whitny U-Test. ACSF=artificial cerebro-spinal fluid.

NCAG 1517															
**slice**	**ACSF +SS**					**RR-Chi-S 5.00 mg/l + SS**					**RR-Chi-S 5.00 mg/l + TBS**				
**time [min]**	**10**	**20**	**30**	**40**	**Mean 20-40**	**50**	**60**	**70**	**80**	**Mean 60-80**	**90**	**100**	**110**	**120**	**Mean 100-120**
**1**	-886,25	-1169,00	-891,34	-908,74	**-989,69**	-1083,00	-1155,00	-960,93	-1079,00	**-1064,98**	-1979,00	-2565,00	-2713,00	-2173,00	**-2483,67**
**2**	-615,72	-1055,00	-1005,00	-976,61	**-1012,20**	-1328,00	-1214,00	-1229,00	-1111,00	**-1184,67**	-2774,00	-2715,00	-2664,00	-2348,00	**-2575,67**
**3**	-1095,00	-906,56	-1047,00	-975,53	**-976,36**	-846,96	-1032,00	-1015,00	-1256,00	**-1101,00**	-2223,00	-2026,00	-2584,00	-2345,00	**-2318,33**
**4**	-666,50	-859,61	-1025,00	-1038,00	**-974,20**	-1363,00	-1230,00	-1240,00	-1073,00	**-1181,00**	-1969,00	-2153,00	-2511,00	-2460,00	**-2374,67**
**Mean**	-815,87	-997,54	-992,09	-974,72	**-988,12**	-1155,24	-1157,75	-1111,23	-1129,75	**-1132,91**	-2236,25	-2364,75	-2618,00	-2331,50	**-2438,08**
**SD**	220,03	141,42	69,32	52,80	**17,46**	240,32	89,82	144,11	85,80	**59,51**	377,25	327,80	88,97	118,45	**114,56**
**SEM**	110,01	70,71	34,66	26,40	8,73	120,16	44,91	72,05	42,90	29,76	188,62	163,90	44,48	59,22	57,28
**P<**	n.s.	n.s.	n.s.	n.s.	n.s.	n.s.	n.s.	n.s.	n.s.	n.s.	n.s.	n.s.	n.s.	n.s.	n.s.

Tab. 11 Effects of RR-Chi-S 5.00 mg/l on pyramidal cell activity in terms of changes of population spike amplitudes. Results from single slices as obtained after single stimuli (SS) or after burst stimuli (TBS). Overview on final results after averaging 4 slices. SD=standard deviation; SEM=standard error of mean. P<=Wilcoxon Mann Whitny U-Test. ACSF=artificial cerebro-spinal fluid.

Report NCAG 15/17 H – Hippocampus slice preparation

NCAG 1517															
slice	ACSF +SS					RR-Chi-S 10.0 mg/l + SS					RR-Chi-S 10.0 mg/l + TBS				
time [min]	10	20	30	40	Mean 20-40	50	60	70	80	Mean 60-80	90	100	110	120	Mean 100-120
1	-1177,00	-874,38	-1005,00	-820,34	**-899,91**	-1068,00	-1538,00	-1238,00	-1076,00	**-1284,00**	-2702,00	-2551,00	-2424,00	-2598,00	**-2524,33**
2	-798,51	-1004,00	-1002,00	-980,50	**-995,50**	-1390,00	-1547,00	-1408,00	-1529,00	**-1494,67**	-3168,00	-3067,00	-2979,00	-3099,00	**-3048,33**
3	-664,81	-1126,00	-1044,00	-1044,00	**-1071,33**	-1694,00	-1780,00	-1739,00	-1991,00	**-1836,67**	-2951,00	-3069,00	-2720,00	-2580,00	**-2789,67**
4	-1135,00	-1230,00	-1087,00	-1087,00	**-1134,67**	-2191,00	-2238,00	-1789,00	-1934,00	**-1987,00**	-2924,00	-2894,00	-2769,00	-3073,00	**-2912,00**
Mean	-943,83	-1058,60	-1034,50	-982,96	**-1025,35**	-1585,75	-1775,75	-1543,50	-1632,50	**-1650,58**	-2936,25	-2895,25	-2723,00	-2837,50	**-2818,58**
SD	251,58	153,67	39,89	116,91	**101,15**	477,64	327,89	264,69	424,20	**319,63**	190,57	243,72	228,81	287,23	**222,81**
SEM	125,79	76,83	19,94	58,45	50,57	238,82	163,95	132,34	212,10	159,82	95,28	121,86	114,40	143,62	111,40
P<	n.s.	n.s.	n.s.	n.s.	n.s.	0.10	0.01	0.01	0.10	0.01	0.01	0.01	0.01	0.01	0.01

Tab. 12 Effects of RR-Chi-S 10.0 mg/l on pyramidal cell activity in terms of changes of population spike amplitudes. Results from single slices as obtained after single stimuli (SS) or after burst stimuli (TBS). Overview on final results after averaging 4 slices. SD=standard deviation; SEM=standard error of mean. P<=Wilcoxon Mann Whitny U-Test. ACSF=artificial cerebro-spinal fluid.

NCAG 1517															
slice	ACSF +SS					RR-Chi-S 20.0 mg/l + SS					RR-Chi-S 20.0 mg/l + TBS				
time [min]	10	20	30	40	Mean 20-40	50	60	70	80	Mean 60-80	90	100	110	120	Mean 100-120
1	-930,20	-1147,00	-1207,00	-1105,00	**-1153,00**	-1848,00	-2043,00	-1990,00	-1979,00	**-2004,00**	-2910,00	-4165,00	-3345,00	-3917,00	**-3809,00**
2	-1028,00	-944,73	-943,43	-1001,00	**-963,05**	-1320,00	-1958,00	-2000,00	-2016,00	**-1991,33**	-3735,00	-3675,00	-3785,00	-3765,00	**-3741,67**
3	-993,54	-953,11	-919,46	-967,43	**-946,67**	-1947,00	-2249,00	-2217,00	-2188,00	**-2218,00**	-3474,00	-3514,00	-3277,00	-3496,00	**-3429,00**
4	-997,12	-991,39	-846,37	-1108,00	**-981,92**	-2601,00	-1933,00	-2097,00	-2179,00	**-2069,67**	-3715,00	-3444,00	-3230,00	-3459,00	**-3377,67**
Mean	-987,22	-1009,06	-979,07	-1045,36	**-1011,16**	-1929,00	-2045,75	-2076,00	-2090,50	**-2070,75**	-3458,50	-3699,50	-3409,25	-3659,25	**-3589,33**
SD	41,04	94,18	157,46	71,93	**95,65**	525,79	143,45	105,66	108,51	**104,00**	384,42	325,05	254,91	219,37	**217,54**
SEM	20,52	47,09	78,73	35,96	47,83	262,89	71,72	52,83	54,25	52,00	192,21	162,53	127,45	109,68	108,77
P<	n.s.	n.s.	n.s.	n.s.	n.s.	0.01	0.01	0.01	0.01	0.01	0.01	0.01	0.01	0.01	0.01

Tab. 13 Effects of RR-Chi-S 20.0 mg/l on pyramidal cell activity in terms of changes of population spike amplitudes. Results from single slices as obtained after single stimuli (SS) or after burst stimuli (TBS). Overview on final results after averaging 4 slices. SD=standard deviation; SEM=standard error of mean. P<=Wilcoxon Mann Whitny U-Test. ACSF=artificial cerebro-spinal fluid.

Report NCAG 15/17 H – Hippocampus slice preparation

NCAG 1517															
**slice**	**ACSF +SS**					**RR-Chi-S 30.0 mg/l + SS**					**RR-Chi-S 30.0 mg/l + TBS**				
**time [min]**	**10**	**20**	**30**	**40**	**Mean 20-40**	**50**	**60**	**70**	**80**	**Mean 60-80**	**90**	**100**	**110**	**120**	**Mean 100-120**
**1**	-886,37	-966,35	-985,26	-1060,00	**-1003,87**	-1250,00	-1788,00	-1971,00	-2039,00	**-1932,67**	-3063,00	-4132,00	-4085,00	-3711,00	**-3976,00**
**2**	-1350,00	-1108,00	-953,25	-1025,00	**-1028,75**	-2152,00	-2401,00	-2220,00	-2140,00	**-2253,67**	-4279,00	-4227,00	-4377,00	-4042,00	**-4215,33**
**3**	-578,02	-1285,00	-1147,00	-810,61	**-1080,87**	-2673,00	-2418,00	-2373,00	-2170,00	**-2320,33**	-3378,00	-3719,00	-3843,00	-3980,00	**-3847,33**
**4**	-882,84	-955,01	-816,85	-1141,00	**-970,95**	-2253,00	-1980,00	-2128,00	-2104,00	**-2070,67**	-3522,00	-4775,00	-4285,00	-4531,00	**-4530,33**
**Mean**	-924,31	-1078,59	-975,59	-1009,15	**-1021,11**	-2082,00	-2146,75	-2173,00	-2113,25	**-2144,33**	-3560,50	-4213,25	-4147,50	-4066,00	**-4142,25**
**SD**	318,48	154,21	135,61	141,00	**46,34**	598,79	313,44	168,36	56,38	**176,22**	515,93	434,60	236,79	341,67	**300,31**
**SEM**	159,24	77,10	67,81	70,50	23,17	299,39	156,72	84,18	28,19	88,11	257,96	217,30	118,39	170,84	150,16
**P<**	n.s.	n.s.	n.s.	n.s.	n.s.	0.01	0.01	0.01	0.01	0.01	0.01	0.01	0.01	0.01	0.01

Tab. 14 Effects of RR-Chi-S 30.0 mg/l on pyramidal cell activity in terms of changes of population spike amplitudes. Results from single slices as obtained after single stimuli (SS) or after burst stimuli (TBS). Overview on final results after averaging 4 slices. SD=standard deviation; SEM=standard error of mean. P<=Wilcoxon Mann Whitny U-Test. ACSF=artificial cerebro-spinal fluid.

NCAG 1517															
**slice**	**ACSF +SS**					**RR-Alt-S 5.00 mg/l + SS**					**RR-Alt-S 5.00 mg/l + TBS**				
**time [min]**	**10**	**20**	**30**	**40**	**Mean 20-40**	**50**	**60**	**70**	**80**	**Mean 60-80**	**90**	**100**	**110**	**120**	**Mean 100-120**
**1**	-786,75	-922,18	-994,67	-1066,00	**-994,28**	-961,66	-1146,00	-1285,00	-1208,00	**-1213,00**	-2305,00	-3191,00	-2651,00	-2754,00	**-2865,33**
**2**	-787,85	-1127,00	-1230,00	-1049,00	**-1135,33**	-1116,00	-1094,00	-1287,00	-1256,00	**-1212,33**	-3221,00	-2841,00	-2532,00	-2827,00	**-2733,33**
**3**	-957,16	-918,04	-988,77	-1143,00	**-1016,60**	-1277,00	-1393,00	-1274,00	-1305,00	**-1324,00**	-2747,00	-2938,00	-2335,00	-2708,00	**-2660,33**
**4**	-933,47	-1059,00	-1090,00	-1117,00	**-1088,67**	-1188,00	-1363,00	-1272,00	-1332,00	**-1322,33**	-2486,00	-2574,00	-2996,00	-2706,00	**-2758,67**
**Mean**	-866,31	-1006,56	-1075,86	-1093,75	**-1058,72**	-1135,67	-1249,00	-1279,50	-1275,25	**-1267,92**	-2689,75	-2886,00	-2628,50	-2748,75	**-2754,42**
**SD**	91,74	103,62	112,75	43,74	**65,04**	133,39	150,96	7,59	54,77	**63,80**	397,93	255,02	277,50	56,68	**84,89**
**SEM**	45,87	51,81	56,37	21,87	32,52	66,70	75,48	3,80	27,38	31,90	198,97	127,51	138,75	28,34	42,44
**P<**	n.s.	n.s.	n.s.	n.s.	n.s.	n.s.	n.s.	0.10	0.10	0.10	0.10	0.01	0.02	0.01	0.01

Tab. 15 Effects of RR-Alt-S 5.00 mg/l on pyramidal cell activity in terms of changes of population spike amplitudes. Results from single slices as obtained after single stimuli (SS) or after burst stimuli (TBS). Overview on final results after averaging 4 slices. SD=standard deviation; SEM=standard error of mean. P<=Wilcoxon Mann Whitny U-Test. ACSF=artificial cerebro-spinal fluid.

NeuroCode AG
Sportparkstr. 9
D-35578 Wetzlar / Germany

EuroPharm, Inc
955 Challenger Drive, Green Bay
Wisconsin 54311, USA

Report NCAG 15/17 H – Hippocampus slice preparation

NCAG 1517															
slice	ACSF +SS					RR-Alt-S 10.0 mg/l + SS					RR-Alt-S 10.0 mg/l + TBS				
time [min]	10	20	30	40	Mean 20-40	50	60	70	80	Mean 60-80	90	100	110	120	Mean 100-120
1	-1095,00	-941,46	-1058,00	-1030,00	**-1009,82**	-1193,00	-1697,00	-2017,00	-1584,00	**-1766,00**	-2673,00	-3716,00	-3516,00	-3059,00	**-3430,33**
2	-999,93	-938,70	-1157,00	-1132,00	**-1075,90**	-1352,00	-1682,00	-1919,00	-1583,00	**-1728,00**	-2892,00	-3385,00	-3852,00	-4248,00	**-3828,33**
3	-1042,00	-1118,00	-1099,00	-1186,00	**-1134,33**	-1640,00	-1732,00	-1632,00	-1670,00	**-1678,00**	-2496,00	-3046,00	-3103,00	-3586,00	**-3245,00**
4	-1135,00	-914,91	-1176,00	-1245,00	**-1111,97**	-1725,00	-1637,00	-1681,00	-1687,00	**-1668,33**	-2926,00	-3276,00	-2962,00	-3410,00	**-3216,00**
Mean	-1067,98	-978,27	-1122,50	-1148,25	**-1083,01**	-1477,50	-1687,00	-1812,25	-1631,00	**-1710,08**	-2746,75	-3355,75	-3358,25	-3575,75	**-3429,92**
SD	59,24	93,91	54,05	91,35	**54,41**	247,89	39,37	185,32	55,29	**45,53**	201,28	278,65	404,49	498,84	**282,07**
SEM	29,62	46,96	27,03	45,67	27,20	123,95	19,69	92,66	27,64	22,77	100,64	139,33	202,25	249,42	141,04
P<	n.s.	n.s.	n.s.	n.s.	n.s.	0.02	0.01	0.01	0.01	0.01	0.02	0.01	0.01	0.01	0.01

Tab. 16 Effects of RR-Alt-S 10.0 mg/l on pyramidal cell activity in terms of changes of population spike amplitudes. Results from single slices as obtained after single stimuli (SS) or after burst stimuli (TBS). Overview on final results after averaging 4 slices. SD=standard deviation; SEM=standard error of mean. P<=Wilcoxon Mann Whitny U-Test. ACSF=artificial cerebro-spinal fluid.

NCAG 1517															
slice	ACSF +SS					RR-Alt-S 20.0 mg/l + SS					RR-Alt-S 20.0 mg/l + TBS				
time [min]	10	20	30	40	Mean 20-40	50	60	70	80	Mean 60-80	90	100	110	120	Mean 100-120
1	-742,36	-936,46	-971,52	-979,71	**-962,56**	-1513,00	-1968,00	-1777,00	-1974,00	**-1906,33**	-3896,00	-4523,00	-4713,00	-4807,00	**-4681,00**
2	-1190,00	-1156,00	-1166,00	-1002,00	**-1108,00**	-2179,00	-1886,00	-2540,00	-2157,00	**-2194,33**	-3613,00	-4055,00	-4198,00	-3881,00	**-4044,67**
3	-1081,00	-1082,00	-1195,00	-1065,00	**-1114,00**	-1428,00	-1795,00	-1929,00	-1840,00	**-1854,67**	-3433,00	-3675,00	-4021,00	-3736,00	**-3810,67**
4	-1145,00	-1085,00	-901,19	-1212,00	**-1066,06**	-1629,00	-2344,00	-2461,00	-2253,00	**-2352,67**	-4166,00	-4381,00	-4034,00	-3887,00	**-4100,67**
Mean	-1039,59	-1064,87	-1058,43	-1064,68	**-1062,66**	-1687,25	-1998,25	-2176,75	-2056,00	**-2077,00**	-3777,00	-4158,50	-4241,50	-4077,75	**-4159,25**
SD	203,14	92,18	144,34	104,65	**70,05**	338,03	241,09	380,32	184,74	**236,87**	321,83	377,21	324,49	491,15	**369,82**
SEM	101,57	46,09	72,17	52,32	35,03	169,01	120,54	190,16	92,37	118,43	160,91	188,60	162,24	245,58	184,91
P<	n.s.	n.s.	n.s.	n.s.	n.s.	0.01	0.01	0.01	0.01	0.01	0.01	0.01	0.01	0.01	0.01

Tab. 17 Effects of RR-Alt-S 20.0 mg/l on pyramidal cell activity in terms of changes of population spike amplitudes. Results from single slices as obtained after single stimuli (SS) or after burst stimuli (TBS). Overview on final results after averaging 4 slices. SD=standard deviation; SEM=standard error of mean. P<=Wilcoxon Mann Whitny U-Test. ACSF=artificial cerebro-spinal fluid.

Report NCAG 15/17 H – Hippocampus slice preparation

NCAG 1517															
**slice**	**ACSF +SS**					**RR-Alt-S 30.0 mg/l + SS**					**RR-Alt-S 30.0 mg/l + TBS**				
**time [min]**	**10**	**20**	**30**	**40**	**Mean 20-40**	**50**	**60**	**70**	**80**	**Mean 60-80**	**90**	**100**	**110**	**120**	**Mean 100-120**
**1**	-626,62	-968,88	-1221,00	-1039,00	**-1076,29**	-2183,00	-2112,00	-1950,00	-2151,00	**-2071,00**	-4245,00	-4126,00	-4426,00	-4485,00	**-4345,67**
**2**	-1258,00	-1056,00	-1211,00	-1173,00	**-1146,67**	-2713,00	-2577,00	-2249,00	-2265,00	**-2363,67**	-4682,00	-4890,00	-4103,00	-4250,00	**-4414,33**
**3**	-949,86	-1189,00	-1162,00	-1171,00	**-1174,00**	-1538,00	-1707,00	-1963,00	-2290,00	**-1986,67**	-3156,00	-3730,00	-3788,00	-4243,00	**-3920,33**
**4**	-571,33	-862,93	-956,85	-1049,00	**-956,26**	-1531,00	-2274,00	-1977,00	-2138,00	**-2129,67**	-3426,00	-3957,00	-4122,00	-4071,00	**-4050,00**
**Mean**	-851,45	-1019,20	-1137,71	-1108,00	**-1088,31**	-1991,25	-2167,50	-2034,75	-2211,00	**-2137,75**	-3877,25	-4175,75	-4109,75	-4262,25	**-4182,58**
**SD**	318,32	138,01	123,30	74,02	**97,18**	570,08	362,48	143,26	77,64	**161,64**	708,68	503,05	260,60	170,01	**235,70**
**SEM**	159,16	69,00	61,65	37,01	48,59	285,04	181,24	71,63	38,82	80,82	354,34	251,52	130,30	85,01	117,85
**P<**	n.s.	n.s.	n.s.	n.s.	n.s.	0.01	0.01	0.01	0.01	0.01	0.01	0.01	0.01	0.01	0.01

Tab. 18 Effects of RR-Alt-S 30.0 mg/l on pyramidal cell activity in terms of changes of population spike amplitudes. Results from single slices as obtained after single stimuli (SS) or after burst stimuli (TBS). Overview on final results after averaging 4 slices. SD=standard deviation; SEM=standard error of mean. P<=Wilcoxon Mann Whitny U-Test. ACSF=artificial cerebro-spinal fluid.

NCAG 1517															
**slice**	**ACSF +SS**					**RR-Alt-B 5.00 mg/l + SS**					**RR-Alt-B 5.00 mg/l + TBS**				
**time [min]**	**10**	**20**	**30**	**40**	**Mean 20-40**	**50**	**60**	**70**	**80**	**Mean 60-80**	**90**	**100**	**110**	**120**	**Mean 100-120**
**1**	-788,99	-1090,00	-1211,00	-1178,00	**-1159,67**	-1021,00	-970,72	-1312,00	-1270,00	**-1184,24**	-1971,00	-2369,00	-2731,00	-2727,00	**-2609,00**
**2**	-708,16	-1062,00	-929,78	-1088,00	**-1026,59**	-1230,00	-1159,00	-1039,00	-898,22	**-1032,07**	-1971,00	-2388,00	-2344,00	-2291,00	**-2341,00**
**3**	-917,49	-1086,00	-1112,00	-1053,00	**-1083,67**	-1228,00	-1200,00	-1066,00	-1162,00	**-1142,67**	-2789,00	-2619,00	-2377,00	-2108,00	**-2368,00**
**4**	-1033,00	-1229,00	-1063,00	-1021,00	**-1104,33**	-1091,00	-1066,00	-1038,00	-1070,00	**-1058,00**	-2413,00	-2521,00	-2468,00	-2342,00	**-2443,67**
**Mean**	-861,91	-1116,75	-1078,95	-1085,00	**-1093,57**	-1142,50	-1098,93	-1113,75	-1100,06	**-1104,25**	-2286,00	-2474,25	-2480,00	-2367,00	**-2440,42**
**SD**	142,97	75,85	116,96	67,77	**54,98**	103,89	102,22	132,80	157,44	**71,23**	394,79	117,83	175,36	260,18	**120,50**
**SEM**	71,48	37,92	58,48	33,88	27,49	51,95	51,11	66,40	78,72	35,62	197,40	58,92	87,68	130,09	60,25
**P<**	n.s.	n.s.	n.s.	n.s.	n.s.	n.s.	n.s.	n.s.	n.s.	n.s.	n.s.	n.s.	n.s.	n.s.	n.s.

Tab. 19 Effects of RR-Alt-B 5.00 mg/l on pyramidal cell activity in terms of changes of population spike amplitudes. Results from single slices as obtained after single stimuli (SS) or after burst stimuli (TBS). Overview on final results after averaging 4 slices. SD=standard deviation; SEM=standard error of mean. P<=Wilcoxon Mann Whitny U-Test. ACSF=artificial cerebro-spinal fluid.

Report NCAG 15/17 H – Hippocampus slice preparation

NCAG 1517															
**slice**	**ACSF +SS**					**RR-Alt-B 10.0 mg/l + SS**					**RR-Alt-B 10.0 mg/l + TBS**				
**time [min]**	**10**	**20**	**30**	**40**	**Mean 20-40**	**50**	**60**	**70**	**80**	**Mean 60-80**	**90**	**100**	**110**	**120**	**Mean 100-120**
**1**	-1084,00	-989,45	-1084,00	-1181,00	**-1084,82**	-1252,00	-1390,00	-1365,00	-1566,00	**-1440,33**	-2535,00	-2964,00	-2534,00	-2326,00	**-2608,00**
**2**	-1083,00	-940,29	-1184,00	-1229,00	**-1117,76**	-727,76	-1503,00	-1615,00	-1560,00	**-1559,33**	-2636,00	-3148,00	-2933,00	-2446,00	**-2842,33**
**3**	-618,06	-985,37	-940,90	-967,85	**-964,71**	-1183,00	-1247,00	-1837,00	-1463,00	**-1515,67**	-3294,00	-2961,00	-2543,00	-2745,00	**-2749,67**
**4**	-802,69	-1020,00	-1090,00	-1146,00	**-1085,33**	-1529,00	-1767,00	-1730,00	-1744,00	***-1747,00**	-2076,00	-2680,00	-3287,00	-3202,00	**-3056,33**
**Mean**	-896,94	-983,78	-1074,73	-1130,96	**-1063,16**	-1172,94	-1476,75	-1636,75	-1583,25	**-1505,11**	-2635,25	-2938,25	-2824,25	-2679,75	**-2814,08**
**SD**	228,23	32,85	100,28	113,94	**67,42**	332,32	220,03	202,58	117,10	**60,20**	502,25	193,11	360,24	390,21	**188,06**
**SEM**	114,12	16,43	50,14	56,97	33,71	166,16	110,02	101,29	58,55	34,80	251,12	96,55	180,12	195,10	94,03
**P<**	n.s.	n.s.	n.s.	n.s.	n.s.	n.s.	0.01	0.01	0.01	0.01	n.s.	0.01	0.01	n.s.	0.01

Tab. 20 Effects of RR-Alt-B 10.0 mg/l on pyramidal cell activity in terms of changes of population spike amplitudes. Results from single slices as obtained after single stimuli (SS) or after burst stimuli (TBS). Overview on final results after averaging 4 slices. SD=standard deviation; SEM=standard error of mean. P<=Wilcoxon Mann Whitny U-Test. ACSF=artificial cerebro-spinal fluid. Outlier ismarked by *.

NCAG 1517															
**slice**	**ACSF +SS**					**RR-Alt-B 20.0 mg/l + SS**					**RR-Alt-B 20.0 mg/l + TBS**				
**time [min]**	**10**	**20**	**30**	**40**	**Mean 20-40**	**50**	**60**	**70**	**80**	**Mean 60-80**	**90**	**100**	**110**	**120**	**Mean 100-120**
**1**	-727,89	-936,24	-898,24	-936,14	**-923,54**	-1774,00	-1652,00	-1954,00	-1646,00	**-1750,67**	-3278,00	-3988,00	-3418,00	-3433,00	**-3613,00**
**2**	-1261,00	-987,97	-1085,00	-1154,00	**-1075,66**	-1570,00	-1649,00	-1784,00	-1774,00	**-1735,67**	-2906,00	-3415,00	-3849,00	-3697,00	**-3653,67**
**3**	-658,37	-1090,00	-977,81	-1056,00	**-1041,27**	-1151,00	-1907,00	-1391,00	-1534,00	**-1610,67**	-3294,00	-3694,00	-3379,00	-3494,00	**-3522,33**
**4**	-813,16	-1037,00	-963,05	-908,65	**-969,57**	-1063,00	-1467,00	-1579,00	-2228,00	**-1758,00**	-3167,00	-3697,00	-3768,00	-3694,00	**-3719,67**
**Mean**	-865,11	-1012,80	-981,03	-1013,70	**-1002,51**	-1389,50	-1668,75	-1677,00	-1795,50	**-1713,75**	-3161,25	-3698,50	-3603,50	-3579,50	**-3627,17**
**SD**	271,42	65,89	77,45	113,32	**68,74**	338,58	180,87	244,66	304,55	**69,35**	179,29	233,96	239,54	136,25	**82,56**
**SEM**	135,71	32,94	38,73	56,66	34,37	169,29	90,43	122,33	152,27	34,67	89,65	116,98	119,77	68,12	41,28
**P<**	n.s.	n.s.	n.s.	n.s.	n.s.	0.01	0.01	0.01	0.01	0.01	0.01	0.01	0.01	0.01	0.01

Tab. 21 Effects of RR-Alt-B 20.0 mg/l on pyramidal cell activity in terms of changes of population spike amplitudes. Results from single slices as obtained after single stimuli (SS) or after burst stimuli (TBS). Overview on final results after averaging 4 slices. SD=standard deviation; SEM=standard error of mean. P<=Wilcoxon Mann Whitny U-Test. ACSF=artificial cerebro-spinal fluid.

Report NCAG 15/17 H – Hippocampus slice preparation

NCAG 1517															
**slice**	**ACSF +SS**					**RR-Alt-B 30.0 mg/l + SS**					**RR-Alt-B 30.0 mg/l + TBS**				
**time [min]**	**10**	**20**	**30**	**40**	**Mean 20-40**	**50**	**60**	**70**	**80**	**Mean 60-80**	**90**	**100**	**110**	**120**	**Mean 100-120**
**1**	-992,07	-994,81	-1145,00	-1269,00	**-1136,27**	-1649,00	-1970,00	-2233,00	-2243,00	**-2148,67**	-2559,00	-4011,00	-3816,00	-4095,00	**-3974,00**
**2**	-923,25	-1014,00	-901,29	-1139,00	**-1018,10**	-1909,00	-1872,00	-2123,00	-2269,00	**-2088,00**	-2902,00	-3787,00	-4130,00	-3843,00	**-3920,00**
**3**	-901,98	-912,75	-916,96	-834,48	**-888,06**	-1415,00	-1889,00	-2110,00	-1954,00	**-1984,33**	-4000,00	-4501,00	-3927,00	-3982,00	**-4136,67**
**4**	-742,86	-944,85	-1253,00	-1194,00	**-1130,62**	-2187,00	-2311,00	-2347,00	-2335,00	**-2331,00**	-3028,00	-4285,00	-3870,00	-4405,00	**-4186,67**
**Mean**	-890,04	-966,60	-1054,06	-1109,12	**-1043,26**	-1790,00	-2010,50	-2203,25	-2200,25	**-2138,00**	-3122,25	-4146,00	-3935,75	-4081,25	**-4054,33**
**SD**	105,38	46,24	173,19	190,69	**116,91**	332,80	204,85	110,58	168,67	**145,46**	617,82	312,22	137,20	239,18	**127,53**
**SEM**	52,69	23,12	86,59	95,34	58,45	166,40	102,42	55,29	84,34	72,73	308,91	156,11	68,60	119,59	63,76
**P<**	n.s.	n.s.	n.s.	n.s.	n.s.	0.01	0.01	0.01	0.01	0.01	0.01	0.01	0.01	0.01	0.01

Tab. 22 Effects of RR-Alt-B 30.0 mg/l on pyramidal cell activity in terms of changes of population spike amplitudes. Results from single slices as obtained after single stimuli (SS) or after burst stimuli (TBS). Overview on final results after averaging 4 slices. SD=standard deviation; SEM=standard error of mean. P<=Wilcoxon Mann Whitny U-Test. ACSF=artificial cerebro-spinal fluid.

NCAG 1517															
**slice**	**ACSF +SS**					**RR-Alt-X 5.00 mg/l + SS**					**RR-Alt-X 5.00 mg/l + TBS**				
**time [min]**	**10**	**20**	**30**	**40**	**Mean 20-40**	**50**	**60**	**70**	**80**	**Mean 60-80**	**90**	**100**	**110**	**120**	**Mean 100-120**
**1**	-445,19	-1188,00	-1127,00	-953,17	**-1089,39**	-1035,00	-1177,00	-1265,00	-1238,00	**-1226,67**	-1929,00	-2228,00	-2256,00	-2159,00	**-2214,33**
**2**	-747,12	-991,03	-1129,00	-954,39	**-1024,81**	-1607,00	-1154,00	-1392,00	-1420,00	**-1322,00**	-1794,00	-2087,00	-2189,00	-2326,00	**-2200,67**
**3**	-700,55	-1137,00	-1102,00	-1107,00	**-1115,33**	-1292,00	-1252,00	-1316,00	-1493,00	**-1353,67**	-1672,00	-2163,00	-2354,00	-2509,00	**-2342,00**
**4**	-1274,00	-732,24	-1037,00	-1015,00	**-928,08**	-725,14	-828,86	-1392,00	-1335,00	**-1185,29**	-2291,00	-2603,00	-2337,00	-2339,00	**-2426,33**
**Mean**	-791,72	-1012,07	-1098,75	-1007,39	**-1039,40**	-1164,79	-1102,97	-1341,25	-1371,50	**-1271,91**	-1921,50	-2270,25	-2284,00	-2333,25	**-2295,83**
**SD**	347,84	204,37	42,96	72,41	**83,41**	375,00	187,47	62,19	109,95	**79,04**	267,76	229,19	76,42	142,99	**107,80**
**SEM**	173,92	102,19	21,48	36,20	41,70	187,50	93,73	31,09	54,98	39,52	133,88	114,60	38,21	71,49	53,90
**P<**	n.s.	n.s.	n.s.	n.s.	n.s.	n.s.	n.s.	n.s.	n.s.	n.s.	n.s.	n.s.	n.s.	n.s.	n.s.

Tab. 23 Effects of RR-Alt-X 5.00 mg/l on pyramidal cell activity in terms of changes of population spike amplitudes. Results from single slices as obtained after single stimuli (SS) or after burst stimuli (TBS). Overview on final results after averaging 4 slices. SD=standard deviation; SEM=standard error of mean. P<=Wilcoxon Mann Whitny U-Test. ACSF=artificial cerebro-spinal fluid.

Report NCAG 15/17 H – Hippocampus slice preparation

NCAG 1517															
**slice**	**ACSF +SS**					**RR-Alt-X 10.0 mg/l + SS**					**RR-Alt-X 10.0 mg/l + TBS**				
**time [min]**	**10**	**20**	**30**	**40**	**Mean 20-40**	**50**	**60**	**70**	**80**	**Mean 60-80**	**90**	**100**	**110**	**120**	**Mean 100-120**
**1**	-730,29	-913,32	-888,69	-1188,00	* **-996,67**	-2634,00	-2791,00	-2799,00	-2561,00	* **-2717,00**	-3437,00	-3648,00	-3678,00	-3475,00	* **-3600,33**
**2**	-1024,00	-1167,00	-1080,00	-875,35	**-1040,78**	-1442,00	-1448,00	-1600,00	-1605,00	**-1551,00**	-2745,00	-2785,00	-2810,00	-2573,00	**-2722,67**
**3**	-464,16	-925,52	-921,26	-1024,00	**-956,93**	-1499,00	-1444,00	-1423,00	-1741,00	**-1536,00**	-2819,00	-2962,00	-3437,00	-3130,00	**-3176,33**
**4**	-866,81	-1271,00	-813,98	-1175,00	**-1086,66**	-1719,00	-1553,00	-1734,00	-1304,00	**-1530,33**	-2518,00	-3036,00	-2991,00	-3310,00	**-3112,33**
**Mean**	-784,99	-1121,17	-938,41	-1024,78	**-1028,12**	-1553,33	-1481,67	-1585,67	-1550,00	**-1539,11**	-2694,00	-2927,67	-3079,33	-3004,33	**-3003,78**
**SD**	288,75	177,24	133,84	149,83	**65,79**	146,27	61,81	155,99	223,63	**10,68**	156,85	128,97	322,70	384,23	**245,54**
**SEM**	144,37	88,62	66,92	74,91	32,89	73,14	30,90	78,00	111,82	5,34	78,42	64,49	161,35	192,12	122,77
**P<**	n.s.	n.s.	n.s.	n.s.	n.s.	0.01	0.01	0.01	0.01	0.01	0.01	0.01	0.01	0.01	0.01

Tab. 24 Effects of RR-Alt-X 10.0 mg/l on pyramidal cell activity in terms of changes of population spike amplitudes. Results from single slices as obtained after single stimuli (SS) or after burst stimuli (TBS). Overview on final results after averaging 4 slices. SD=standard deviation; SEM=standard error of mean. P<=Wilcoxon Mann Whitny U-Test. ACSF=artificial cerebro-spinal fluid. Outliers are marked by *.

NCAG 1517															
**slice**	**ACSF +SS**					**RR-Alt-X 20.0 mg/l + SS**					**RR-Alt-X 20.0 mg/l + TBS**				
**time [min]**	**10**	**20**	**30**	**40**	**Mean 20-40**	**50**	**60**	**70**	**80**	**Mean 60-80**	**90**	**100**	**110**	**120**	**Mean 100-120**
**1**	-1093,00	-1028,00	-1020,00	-888,21	**-978,74**	-1453,00	-2034,00	-1918,00	-1810,00	**-1920,67**	-3502,00	-3636,00	-3488,00	-3788,00	**-3637,33**
**2**	-1041,00	-954,76	-986,11	-965,70	**-968,86**	-2138,00	-2037,00	-2040,00	-1910,00	**-1995,67**	-3720,00	-3546,00	-3501,00	-3288,00	**-3445,00**
**3**	-1443,00	-933,23	-1127,00	-1114,00	**-1058,08**	-1704,00	-1930,00	-1768,00	-1995,00	**-1897,67**	-2960,00	-3425,00	-3244,00	-3282,00	**-3317,00**
**4**	-943,59	-947,15	-1037,00	-996,87	**-993,67**	-1814,00	-1811,00	-1793,00	-2063,00	**-1889,00**	-2785,00	-2953,00	-3696,00	-3145,00	**-3264,67**
**Mean**	-1130,15	-965,79	-1042,53	-991,20	**-999,84**	-1777,25	-1953,00	-1879,75	-1944,50	**-1925,75**	-3241,75	-3390,00	-3482,25	-3375,75	**-3416,00**
**SD**	217,57	42,42	60,16	93,75	**40,14**	284,02	106,94	125,38	109,35	**48,49**	441,38	303,89	185,15	282,66	**165,86**
**SEM**	108,78	21,21	30,08	46,88	20,07	142,01	53,47	62,69	54,68	24,24	220,69	151,94	92,57	141,33	82,93
**P<**	n.s.	n.s.	n.s.	n.s.	n.s.	0.01	0.01	0.01	0.01	0.01	0.01	0.01	0.01	0.01	0.01

Tab. 25 Effects of RR-Alt-X 20.0 mg/l on pyramidal cell activity in terms of changes of population spike amplitudes. Results from single slices as obtained after single stimuli (SS) or after burst stimuli (TBS). Overview on final results after averaging 4 slices. SD=standard deviation; SEM=standard error of mean. P<=Wilcoxon Mann Whitny U-Test. ACSF=artificial cerebro-spinal fluid.

Report NCAG 15/17 H – Hippocampus slice preparation

NCAG 1517															
**slice**	**ACSF +SS**					**RR-Alt-X 30.0 mg/l + SS**					**RR-Alt-X 30.0 mg/l + TBS**				
**time [min]**	**10**	**20**	**30**	**40**	**Mean 20-40**	**50**	**60**	**70**	**80**	**Mean 60-80**	**90**	**100**	**110**	**120**	**Mean 100-120**
**1**	-1030,00	-1136,00	-1049,00	-1057,00	**-1080,67**	-1341,00	-2091,00	-2400,00	-1787,00	**-2092,67**	-4200,00	-4726,00	-4496,00	-4404,00	**-4542,00**
**2**	-545,97	-931,08	-949,99	-1232,00	**-1037,69**	-2872,00	-1713,00	-2263,00	-2565,00	**-2180,33**	-4965,00	-4490,00	-4786,00	-4115,00	**-4463,67**
**3**	-1021,00	-1287,00	-1222,00	-1132,00	**-1213,67**	-2531,00	-2434,00	-2264,00	-2090,00	**-2262,67**	-3934,00	-3945,00	-4431,00	-3982,00	**-4119,33**
**4**	-886,78	-957,11	-1199,00	-1162,00	**-1106,04**	-1888,00	-1852,00	-2055,00	-2345,00	**-2084,00**	-3549,00	-4136,00	-4601,00	-4717,00	**-4484,67**
**Mean**	-870,94	-1077,80	-1105,00	-1145,75	**-1109,52**	-2158,00	-2022,50	-2245,50	-2196,75	**-2154,92**	-4162,00	-4324,25	-4578,50	-4304,50	**-4402,42**
**SD**	226,33	166,58	128,70	72,50	**74,95**	680,52	315,64	142,37	335,10	**83,98**	598,33	350,31	155,05	326,58	**191,60**
**SEM**	113,16	83,29	64,35	36,25	37,47	340,26	157,82	71,19	167,55	41,99	299,17	175,15	77,53	163,29	95,80
**P<**	n.s.	n.s.	n.s.	n.s.	n.s.	0.01	0.01	0.01	0.01	0.01	0.01	0.01	0.01	0.01	0.01

Tab. 26 Effects of RR-Alt-X 30.0 mg/l on pyramidal cell activity in terms of changes of population spike amplitudes. Results from single slices as obtained after single stimuli (SS) or after burst stimuli (TBS). Overview on final results after averaging 4 slices. SD=standard deviation; SEM=standard error of mean. P<=Wilcoxon Mann Whitny U-Test. ACSF=artificial cerebro-spinal fluid.

NCAG 1517															
**slice**	**ACSF +SS**					**RR-Alt-G 5.00 mg/l + SS**					**RR-Alt-G 5.00 mg/l + TBS**				
**time [min]**	**10**	**20**	**30**	**40**	**Mean 20-40**	**50**	**60**	**70**	**80**	**Mean 60-80**	**90**	**100**	**110**	**120**	**Mean 100-120**
**1**	-1015,00	-985,39	-995,36	-1030,00	**-1003,58**	-1102,00	-1126,00	-1098,00	-1103,00	**-1109,00**	-2592,00	-2490,00	-2394,00	-2074,00	**-2319,33**
**2**	-1086,00	-966,24	-1109,00	-1185,00	**-1086,75**	-1004,00	-1354,00	-1088,00	-1217,00	**-1219,67**	-1753,00	-2573,00	-2188,00	-2244,00	**-2335,00**
**3**	-1006,00	-914,13	-996,07	-1074,00	**-994,73**	-1220,00	-1317,00	-1173,00	-1233,00	**-1241,00**	-2627,00	-2616,00	-2668,00	-2799,00	**-2694,33**
**4**	-1170,00	-1090,00	-1041,00	-1352,00	**-1161,00**	-1653,00	-1286,00	-1253,00	-1208,00	**-1249,00**	-1293,00	-2302,00	-2566,00	-2628,00	**-2498,67**
**Mean**	-1069,25	-988,94	-1035,36	-1160,25	**-1061,52**	-1244,75	-1270,75	-1153,00	-1190,25	**-1204,67**	-2066,25	-2495,25	-2454,00	-2436,25	**-2461,83**
**SD**	76,10	73,80	53,54	143,51	**78,21**	286,13	100,42	76,70	59,08	**64,97**	654,95	139,04	210,31	334,93	**174,93**
**SEM**	38,05	36,90	26,77	71,75	39,10	143,07	50,21	38,35	29,54	32,48	327,48	69,52	105,16	167,47	87,47
**P<**	n.s.	n.s.	n.s.	n.s.	n.s.	n.s.	n.s.	n.s.	n.s.	n.s.	n.s.	n.s.	n.s.	n.s.	n.s.

Tab. 27 Effects of RR-Alt-G 5.00 mg/l on pyramidal cell activity in terms of changes of population spike amplitudes. Results from single slices as obtained after single stimuli (SS) or after burst stimuli (TBS). Overview on final results after averaging 4 slices. SD=standard deviation; SEM=standard error of mean. P<=Wilcoxon Mann Whitny U-Test. ACSF=artificial cerebro-spinal fluid.

Report NCAG 15/17 H – Hippocampus slice preparation

NCAG 1517															
**slice**	**ACSF +SS**					**RR-Alt-G 10.0 mg/l + SS**					**RR-Alt-G 10.0 mg/l + TBS**				
**time [min]**	**10**	**20**	**30**	**40**	**Mean 20-40**	**50**	**60**	**70**	**80**	**Mean 60-80**	**90**	**100**	**110**	**120**	**Mean 100-120**
**1**	-1143,00	-1105,00	-1094,00	-1142,00	**-1113,67**	-1195,00	-1914,00	-1190,00	-1276,00	**-1460,00**	-2171,00	-2566,00	-2968,00	-3104,00	**-2879,33**
**2**	-1086,00	-973,95	-1182,00	-1042,00	**-1065,98**	-1594,00	-1600,00	-1475,00	-1610,00	**-1561,67**	-2029,00	-2655,00	-2547,00	-2426,00	**-2542,67**
**3**	-875,84	-804,84	-878,66	-992,39	**-891,96**	-750,14	-1477,00	-1483,00	-1573,00	**-1511,00**	-2476,00	-2628,00	-2555,00	-2713,00	**-2632,00**
**4**	-1053,00	-1053,00	-901,10	-998,93	**-984,34**	-1758,00	-2015,00	-1739,00	-1449,00	**-1734,33**	-2576,00	-2777,00	-2719,00	-2680,00	**-2725,33**
**Mean**	-1039,46	-984,20	-1013,94	-1043,83	**-1013,99**	-1324,29	-1751,50	-1471,75	-1477,00	**-1566,75**	-2313,00	-2656,50	-2697,25	-2730,75	**-2694,83**
**SD**	115,24	131,15	147,97	69,05	**97,31**	449,89	254,39	224,31	150,65	**119,18**	255,97	88,55	197,14	279,93	**143,84**
**SEM**	57,62	65,58	73,99	34,52	48,66	224,95	127,19	112,15	75,33	59,59	127,98	44,28	98,57	139,96	71,92
**P<**	n.s.	n.s.	n.s.	n.s.	n.s.	n.s.	0.01	0.01	0.01	0.01	n.s.	0.01	0.01	0.02	0.01

Tab. 28 Effects of RR-Alt-G 10.0 mg/l on pyramidal cell activity in terms of changes of population spike amplitudes. Results from single slices as obtained after single stimuli (SS) or after burst stimuli (TBS). Overview on final results after averaging 4 slices. SD=standard deviation; SEM=standard error of mean. P<=Wilcoxon Mann Whitny U-Test. ACSF=artificial cerebro-spinal fluid.

NCAG 1517															
**slice**	**ACSF +SS**					**RR-Alt-G 20.0 mg/l + SS**					**RR-Alt-G 20.0 mg/l + TBS**				
**time [min]**	**10**	**20**	**30**	**40**	**Mean 20-40**	**50**	**60**	**70**	**80**	**Mean 60-80**	**90**	**100**	**110**	**120**	**Mean 100-120**
**1**	-1162,00	-860,33	-803,67	-867,05	**-843,68**	-1481,00	-1919,00	-1682,00	-2191,00	**-1930,67**	-3853,00	-3085,00	-3995,00	-3692,00	**-3590,67**
**2**	-779,77	-960,52	-1131,00	-929,37	**-1006,96**	-1450,00	-1722,00	-1751,00	-1764,00	**-1745,67**	-3640,00	-3552,00	-3655,00	-3709,00	**-3638,67**
**3**	-971,95	-1187,00	-1072,00	-950,08	**-1069,69**	-2149,00	-2302,00	-2200,00	-2170,00	**-2224,00**	-2633,00	-3464,00	-3696,00	-2933,00	**-3364,33**
**4**	-856,88	-944,74	-1151,00	-1285,00	**-1126,91**	-2058,00	-2229,00	-1863,00	-2424,00	**-2172,00**	-3444,00	-3481,00	-3679,00	-3492,00	**-3550,67**
**Mean**	-942,65	-988,15	-1039,42	-1007,88	**-1011,81**	-1784,50	-2043,00	-1874,00	-2137,25	**-2018,08**	-3392,50	-3395,50	-3756,25	-3456,50	**-3536,08**
**SD**	166,19	139,67	160,70	188,09	**122,32**	370,43	270,86	229,78	274,17	**222,07**	533,17	210,48	160,05	362,64	**120,02**
**SEM**	83,10	69,84	80,35	94,04	61,16	185,22	135,43	114,89	137,08	111,03	266,58	105,24	80,03	181,32	60,01
**P<**	n.s.	n.s.	n.s.	n.s.	n.s.	0.01	0.01	0.01	0.01	0.01	0.01	0.01	0.01	0.01	0.01

Tab. 29 Effects of RR-Alt-G 20.0 mg/l on pyramidal cell activity in terms of changes of population spike amplitudes. Results from single slices as obtained after single stimuli (SS) or after burst stimuli (TBS). Overview on final results after averaging 4 slices. SD=standard deviation; SEM=standard error of mean. P<=Wilcoxon Mann Whitny U-Test. ACSF=artificial cerebro-spinal fluid.

Report NCAG 15/17 H – Hippocampus slice preparation

NCAG 1517															
**slice**	**ACSF +SS**					**RR-Alt-G 30.0 mg/l + SS**					**RR-Alt-G 30.0 mg/l + TBS**				
**time [min]**	**10**	**20**	**30**	**40**	**Mean 20-40**	**50**	**60**	**70**	**80**	**Mean 60-80**	**90**	**100**	**110**	**120**	**Mean 100-120**
**1**	-742,19	-989,34	-968,15	-845,76	**-934,42**	-1799,00	-2469,00	-2553,00	-2478,00	**-2500,00**	-2954,00	-4942,00	-4913,00	-3398,00	**-4417,67**
**2**	-1088,00	-1230,00	-1010,00	-725,78	**-988,59**	-2007,00	-1876,00	-1915,00	-2049,00	**-1946,67**	-4977,00	-4881,00	-3872,00	-4385,00	**-4379,33**
**3**	-678,44	-1385,00	-1035,00	-902,39	**-1107,46**	-2022,00	-2482,00	-2557,00	-2357,00	**-2465,33**	-3953,00	-3802,00	-4486,00	-4152,00	**-4146,67**
**4**	-1193,00	-1169,00	-1084,00	-1073,00	**-1108,67**	-2525,00	-2237,00	-2472,00	-2220,00	**-2309,67**	-3633,00	-3732,00	-3947,00	-3964,00	**-3881,00**
**Mean**	-925,41	-1193,34	-1024,29	-886,73	**-1034,79**	-2088,25	-2266,00	-2374,25	-2276,00	**-2305,42**	-3879,25	-4339,25	-4304,50	-3974,75	**-4206,17**
**SD**	253,38	163,59	48,43	144,37	**87,46**	308,44	283,32	308,66	184,42	**253,08**	842,07	661,86	489,24	421,30	**247,65**
**SEM**	126,69	81,80	24,21	72,18	43,73	154,22	141,66	154,33	92,21	126,54	421,03	330,93	244,62	210,65	123,83
**P<**	n.s.	n.s.	n.s.	n.s.	n.s.	0.01	0.01	0.01	0.01	0.01	0.01	0.01	0.01	0.01	0.01

Tab. 30 Effects of RR-Alt-G 30.0 mg/l on pyramidal cell activity in terms of changes of population spike amplitudes. Results from single slices as obtained after single stimuli (SS) or after burst stimuli (TBS). Overview on final results after averaging 4 slices. SD=standard deviation; SEM=standard error of mean. P<=Wilcoxon Mann Whitny U-Test. ACSF=artificial cerebro-spinal fluid.

NCAG 1517															
**slice**	**ACSF +SS**					**Rosavin 0.25 mg/l + SS**					**Rosavin 0.25 mg/l + TBS**				
**time [min]**	**10**	**20**	**30**	**40**	**Mean 20-40**	**50**	**60**	**70**	**80**	**Mean 60-80**	**90**	**100**	**110**	**120**	**Mean 100-120**
**1**	-795,57	-1285,00	-1062,00	-1169,00	**-1172,00**	-1205,00	-1153,00	-1505,00	-1690,00	**-1449,33**	-2868,00	-2811,00	-3048,00	-2709,00	**-2856,00**
**2**	-734,49	-983,34	-965,10	-1072,00	**-1006,81**	-1250,00	-1262,00	-1260,00	-1235,00	**-1252,33**	-2084,00	-2017,00	-2264,00	-2218,00	**-2166,33**
**3**	-728,04	-973,01	-944,83	-1076,00	**-997,95**	-1105,00	-1266,00	-1247,00	-1270,00	**-1261,00**	-2328,00	-2649,00	-2578,00	-2396,00	**-2541,00**
**4**	-832,76	-933,13	-1140,00	-885,06	**-986,06**	-1011,00	-1360,00	-1267,00	-1208,00	**-1278,33**	-2238,00	-2401,00	-2438,00	-2810,00	**-2549,67**
**Mean**	-772,72	-1043,62	-1027,98	-1050,52	**-1040,71**	-1142,75	-1260,25	-1319,75	-1350,75	**-1310,25**	-2379,50	-2469,50	-2582,00	-2533,25	**-2528,25**
**SD**	50,28	162,37	90,51	119,06	**87,94**	106,71	84,63	123,78	227,59	**93,35**	340,89	345,59	336,17	274,29	**282,27**
**SEM**	25,14	81,18	45,25	59,53	43,97	53,35	42,32	61,89	113,79	46,68	170,45	172,79	168,09	137,14	141,13
**P<**	n.s.	n.s.	n.s.	n.s.	n.s.	n.s.	0.10	0.05	0.05	0.05	n.s.	n.s.	n.s.	n.s.	n.s.

Tab. 31 Effects of Rosavin 0.25 mg/l on pyramidal cell activity in terms of changes of population spike amplitudes. Results from single slices as obtained after single stimuli (SS) or after burst stimuli (TBS). Overview on final results after averaging 4 slices. SD=standard deviation; SEM=standard error of mean. P<=Wilcoxon Mann Whitny U-Test. ACSF=artificial cerebro-spinal fluid.

NeuroCode AG
Sportparkstr. 9
D-35578 Wetzlar / Germany

EuroPharm, Inc
955 Challenger Drive, Green Bay
Wisconsin 54311, USA

Report NCAG 15/17 H – Hippocampus slice preparation

NCAG 1517															
**slice**	**ACSF +SS**					**Rosavin 0.50 mg/l + SS**					**Rosavin 0.50 mg/l + TBS**				
**time [min]**	**10**	**20**	**30**	**40**	**Mean 20-40**	**50**	**60**	**70**	**80**	**Mean 60-80**	**90**	**100**	**110**	**120**	**Mean 100-120**
**1**	-803,97	-1024,00	-1168,00	-1148,00	**-1113,33**	-1301,00	-1568,00	-1678,00	-1667,00	**-1637,67**	-2911,00	-2927,00	-2707,00	-2847,00	**-2827,00**
**2**	-1128,00	-990,53	-896,68	-912,55	**-933,25**	-1391,00	-1821,00	-1820,00	-1693,00	**-1778,00**	-2953,00	-3019,00	-2662,00	-2825,00	**-2835,33**
**3**	-994,49	-1224,00	-975,59	-1197,00	**-1132,20**	-1513,00	-1718,00	-1767,00	-1756,00	**-1747,00**	-2746,00	-2506,00	-2877,00	-2949,00	**-2777,33**
**4**	-994,36	-984,69	-1074,00	-921,45	**-993,38**	-1859,00	-1858,00	-1896,00	-1921,00	**-1891,67**	-2691,00	-3113,00	-2967,00	-2937,00	**-3005,67**
**Mean**	-980,21	-1055,81	-1028,57	-1044,75	**-1043,04**	-1516,00	-1741,25	-1790,25	-1759,25	**-1763,58**	-2825,25	-2891,25	-2803,25	-2889,50	**-2861,33**
**SD**	133,30	113,46	117,91	148,91	**95,58**	244,61	129,80	91,67	114,12	**104,47**	126,46	267,82	143,14	62,62	**99,57**
**SEM**	66,65	56,73	58,95	74,45	47,79	122,31	64,90	45,83	57,06	52,24	63,23	133,91	71,57	31,31	49,79
**P<**	n.s.	n.s.	n.s.	n.s.	n.s.	0.01	0.01	0.01	0.01	0.01	0.01	0.01	0.01	0.01	0.01

Tab. 32 Effects of Rosavin 0.50 mg/l on pyramidal cell activity in terms of changes of population spike amplitudes. Results from single slices as obtained after single stimuli (SS) or after burst stimuli (TBS). Overview on final results after averaging 4 slices. SD=standard deviation; SEM=standard error of mean. P<=Wilcoxon Mann Whitny U-Test. ACSF=artificial cerebro-spinal fluid.

NCAG 1517															
**slice**	**ACSF +SS**					**Rosavin 0.75 mg/l + SS**					**Rosavin 0.75 mg/l + TBS**				
**time [min]**	**10**	**20**	**30**	**40**	**Mean 20-40**	**50**	**60**	**70**	**80**	**Mean 60-80**	**90**	**100**	**110**	**120**	**Mean 100-120**
**1**	-697,46	-1069,00	-945,09	-1047,00	**-1020,36**	-1643,00	-2669,00	-2349,00	-2149,00	**-2389,00**	-4229,00	-4334,00	-4114,00	-3924,00	**-4124,00**
**2**	-714,73	-1020,00	-984,82	-980,23	**-995,02**	-1410,00	-1824,00	-1957,00	-2047,00	**-1942,67**	-3396,00	-4153,00	-4330,00	-4339,00	**-4274,00**
**3**	-638,11	-1029,00	-983,84	-1307,00	**-1106,61**	-1900,00	-1860,00	-1875,00	-1978,00	**-1904,33**	-4405,00	-4975,00	-4650,00	-3708,00	**-4444,33**
**4**	-991,26	-1105,00	-976,94	-1203,00	**-1094,98**	-1863,00	-2190,00	-2207,00	-2087,00	**-2161,33**	-2810,00	-3809,00	-4116,00	-4079,00	**-4001,33**
**Mean**	-760,39	-1055,75	-972,67	-1134,31	**-1054,24**	-1704,00	-2135,75	-2097,00	-2065,25	**-2099,33**	-3710,00	-4317,75	-4302,50	-4012,50	**-4210,92**
**SD**	157,37	39,14	18,72	148,22	**54,95**	226,46	391,80	219,46	71,72	**223,85**	744,08	489,29	252,87	265,57	**191,44**
**SEM**	78,69	19,57	9,36	74,11	27,47	113,23	195,90	109,73	35,86	111,92	372,04	244,64	126,43	132,78	95,72
**P<**	n.s.	n.s.	n.s.	n.s.	n.s.	0.01	0.01	0.01	0.01	0.01	0.01	0.01	0.01	0.01	0.01

Tab. 33 Effects of Rosavin 0.75 mg/l on pyramidal cell activity in terms of changes of population spike amplitudes. Results from single slices as obtained after single stimuli (SS) or after burst stimuli (TBS). Overview on final results after averaging 4 slices. SD=standard deviation; SEM=standard error of mean. P<=Wilcoxon Mann Whitny U-Test. ACSF=artificial cerebro-spinal fluid.

Report NCAG 15/17 H – Hippocampus slice preparation

NCAG 1517															
slice	ACSF +SS					Rosavin 1.50 mg/l + SS					Rosavin 1.50 mg/l + TBS				
time [min]	10	20	30	40	Mean 20-40	50	60	70	80	Mean 60-80	90	100	110	120	Mean 100-120
1	-1142,00	-1004,00	-1121,00	-1206,00	**-1110,33**	-1961,00	-1815,00	-2186,00	-2390,00	**-2130,33**	-3992,00	-4236,00	-4047,00	-4349,00	**-4210,67**
2	-966,39	-1201,00	-1054,00	-1130,00	**-1128,33**	-2026,00	-2226,00	-2272,00	-2389,00	**-2295,67**	-3988,00	-4054,00	-4171,00	-4051,00	**-4092,00**
3	-1208,00	-1113,00	-1104,00	-1305,00	**-1174,00**	-1721,00	-1852,00	-2183,00	-2448,00	**-2161,00**	-2938,00	-3837,00	-4322,00	-4314,00	**-4157,67**
4	-963,05	-1080,00	-929,53	-1018,00	**-1009,18**	-2455,00	-2149,00	-1978,00	-2277,00	**-2134,67**	-3582,00	-4187,00	-4947,00	-4582,00	**-4572,00**
Mean	-1069,86	-1099,50	-1052,13	-1164,75	**-1105,46**	-2040,75	-2010,50	-2154,75	-2376,00	**-2180,42**	-3625,00	-4078,50	-4371,75	-4324,00	**-4258,08**
SD	124,37	81,62	86,54	121,26	**69,56**	305,73	207,34	124,85	71,53	**78,02**	496,75	178,42	399,65	217,42	**214,83**
SEM	62,18	40,81	43,27	60,63	34,78	152,87	103,67	62,43	35,77	39,01	248,37	89,21	199,82	108,71	107,42
P<	n.s.	n.s.	n.s.	n.s.	n.s.	0.01	0.01	0.01	0.01	0.01	0.01	0.01	0.01	0.01	0.01

Tab. 34 Effects of Rosavin 1.50 mg/l on pyramidal cell activity in terms of changes of population spike amplitudes. Results from single slices as obtained after single stimuli (SS) or after burst stimuli (TBS). Overview on final results after averaging 4 slices. SD=standard deviation; SEM=standard error of mean. P<=Wilcoxon Mann Whitny U-Test. ACSF=artificial cerebro-spinal fluid.

NCAG 1517															
slice	ACSF +SS					Salidroside 0.25 mg/l + SS					Salidroside 0.25 mg/l + TBS				
time [min]	10	20	30	40	Mean 20-40	50	60	70	80	Mean 60-80	90	100	110	120	Mean 100-120
1	-910,75	-975,53	-1127,00	-863,22	**-988,58**	-899,98	-1168,00	-1265,00	-1158,00	**-1197,00**	-2223,00	-2285,00	-2194,00	-2215,00	**-2231,33**
2	-895,50	-956,09	-1058,00	-1170,00	**-1061,36**	-1594,00	-1306,00	-1331,00	-1229,00	**-1288,67**	-2109,00	-2062,00	-2051,00	-2163,00	**-2092,00**
3	-984,24	-1045,00	-1013,00	-1111,00	**-1056,33**	-1281,00	-1349,00	-1195,00	-1305,00	**-1283,00**	-2851,00	-2137,00	-2257,00	-2597,00	**-2330,33**
4	-916,88	-1162,00	-1206,00	-998,13	**-1122,04**	-1091,00	-1334,00	-1204,00	-1235,00	**-1257,67**	-1975,00	-2661,00	-2415,00	-2218,00	**-2431,33**
Mean	-926,84	-1034,66	-1101,00	-1035,59	**-1057,08**	-1216,50	-1289,25	-1248,75	-1231,75	**-1256,58**	-2289,50	-2286,25	-2229,25	-2298,25	**-2271,25**
SD	39,31	93,08	84,25	135,24	**54,56**	295,86	82,77	63,04	60,06	**41,95**	387,81	266,46	150,87	200,76	**144,73**
SEM	19,65	46,54	42,12	67,62	27,28	147,93	41,39	31,52	30,03	20,97	193,91	133,23	75,44	100,38	72,37
P<	n.s.	n.s.	n.s.	n.s.	n.s.	n.s.	n.s.	n.s.	n.s.	n.s.	n.s.	n.s.	n.s.	n.s.	n.s.

Tab. 35 Effects of Salidroside 0.25 mg/l on pyramidal cell activity in terms of changes of population spike amplitudes. Results from single slices as obtained after single stimuli (SS) or after burst stimuli (TBS). Overview on final results after averaging 4 slices. SD=standard deviation; SEM=standard error of mean. P<=Wilcoxon Mann Whitny U-Test. ACSF=artificial cerebro-spinal fluid.

Report NCAG 15/17 H – Hippocampus slice preparation

NCAG 1517															
slice	ACSF +SS					Salidroside 0.50 mg/l + SS					Salidroside 0.50 mg/l + TBS				
time [min]	10	20	30	40	Mean 20-40	50	60	70	80	Mean 60-80	90	100	110	120	Mean 100-120
1	-874,30	-1031,00	-974,38	-1055,00	**-1020,13**	-1278,00	-1539,00	-1710,00	-1612,00	**-1620,33**	-2826,00	-3273,00	-3245,00	-3270,00	**-3262,67**
2	-955,90	-1137,00	-954,00	-1085,00	**-1058,67**	-1590,00	-1690,00	-1465,00	-1532,00	**-1562,33**	-2564,00	-3231,00	-3155,00	-3186,00	**-3190,67**
3	-769,15	-929,22	-922,32	-928,81	**-926,78**	-1511,00	-1595,00	-1671,00	-1634,00	**-1633,33**	-2344,00	-3430,00	-3403,00	-3350,00	**-3394,33**
4	-667,61	-988,58	-984,84	-969,92	**-981,11**	-1104,00	-1560,00	-1827,00	-1710,00	**-1699,00**	-2860,00	-3187,00	-3183,00	-2705,00	**-3025,00**
Mean	-816,74	-1021,45	-958,89	-1009,68	**-996,67**	-1370,75	-1596,00	-1668,25	-1622,00	**-1628,75**	-2648,50	-3280,25	-3246,50	-3127,75	**-3218,17**
SD	125,41	87,62	27,54	72,68	**56,33**	221,73	66,79	150,85	73,23	**56,09**	242,28	105,83	110,90	289,68	**153,93**
SEM	62,71	43,81	13,77	36,34	28,17	110,87	33,39	75,42	36,62	28,04	121,14	52,91	55,45	144,84	76,97
P<	n.s.	n.s.	n.s.	n.s.	n.s.	0.10	0.01	0.01	0.01	0.01	0.10	0.01	0.01	0.01	0.01

Tab. 36 Effects of Salidroside 0.50 mg/l on pyramidal cell activity in terms of changes of population spike amplitudes. Results from single slices as obtained after single stimuli (SS) or after burst stimuli (TBS). Overview on final results after averaging 4 slices. SD=standard deviation; SEM=standard error of mean. P<=Wilcoxon Mann Whitny U-Test. ACSF=artificial cerebro-spinal fluid.

NCAG 1517															
slice	ACSF +SS					Salidroside 0.75 mg/l + SS					Salidroside 0.75 mg/l + TBS				
time [min]	10	20	30	40	Mean 20-40	50	60	70	80	Mean 60-80	90	100	110	120	Mean 100-120
1	-514,15	-1147,00	-1274,00	-1027,00	**-1149,33**	-1805,00	-1726,00	-1758,00	-1923,00	**-1802,33**	-3313,00	-4028,00	-4282,00	-4781,00	**-4363,67**
2	-775,64	-1319,00	-1166,00	-942,65	**-1142,55**	-1636,00	-1911,00	-2128,00	-2056,00	**-2031,67**	-3356,00	-3422,00	-4195,00	-3783,00	**-3800,00**
3	-705,12	-830,69	-884,11	-1009,00	**-907,93**	-1729,00	-2187,00	-2118,00	-2021,00	**-2108,67**	-3755,00	-3782,00	-3714,00	-3815,00	**-3770,33**
4	-784,14	-1087,00	-1228,00	-1105,00	**-1140,00**	-2732,00	-2724,00	-2133,00	-2227,00	**-2361,33**	-3657,00	-3806,00	-4211,00	-4228,00	**-4081,67**
Mean	-694,76	-1095,92	-1138,03	-1020,91	**-1084,95**	-1975,50	-2137,00	-2034,25	-2056,75	**-2076,00**	-3520,25	-3759,50	-4100,50	-4151,75	**-4003,92**
SD	125,51	202,32	174,97	66,77	**118,08**	509,05	434,77	184,27	126,69	**230,46**	218,89	250,78	260,43	465,89	**277,85**
SEM	62,75	101,16	87,48	33,38	59,04	254,52	217,38	92,14	63,35	115,23	109,45	125,39	130,21	232,94	138,93
P<	0.10	n.s.	n.s.	n.s.	n.s.	0.01	0.01	0.01	0.01	0.01	0.01	0.01	0.01	0.01	0.01

Tab. 37 Effects of Salidroside 0.75 mg/l on pyramidal cell activity in terms of changes of population spike amplitudes. Results from single slices as obtained after single stimuli (SS) or after burst stimuli (TBS). Overview on final results after averaging 4 slices. SD=standard deviation; SEM=standard error of mean. P<=Wilcoxon Mann Whitny U-Test. ACSF=artificial cerebro-spinal fluid.

Report NCAG 15/17 H – Hippocampus slice preparation

NCAG 1517															
slice	ACSF +SS					Salidroside 1.50 mg/l + SS					Salidroside 1.50 mg/l + TBS				
time [min]	10	20	30	40	Mean 20-40	50	60	70	80	Mean 60-80	90	100	110	120	Mean 100-120
1	-726,50	-931,86	-932,73	-857,10	**-907,23**	-1295,00	-1515,00	-1760,00	-1697,00	**-1657,33**	-4099,00	-4368,00	-4292,00	-3995,00	**-4218,33**
2	-776,46	-1129,00	-1184,00	-1237,00	**-1183,33**	-2267,00	-1885,00	-1814,00	-1731,00	**-1810,00**	-3560,00	-3968,00	-4068,00	-3889,00	**-3975,00**
3	-952,79	-992,72	-967,31	-909,22	**-956,42**	-1944,00	-2297,00	-2131,00	-2269,00	**-2232,33**	-3986,00	-4502,00	-4140,00	-3962,00	**-4201,33**
4	-1052,00	-887,43	-1222,00	-1078,00	**-1062,48**	-1785,00	-2256,00	-1920,00	-2160,00	**-2112,00**	-3575,00	-3952,00	-4121,00	-3861,00	**-3978,00**
Mean	-876,94	-985,25	-1076,51	-1020,33	**-1027,36**	-1822,75	-1988,25	-1906,25	-1964,25	**-1952,92**	-3805,00	-4197,50	-4155,25	-3926,75	**-4093,17**
SD	151,80	105,10	147,56	172,49	**122,51**	404,97	365,90	163,91	292,70	**265,31**	278,16	279,72	96,12	62,31	**134,90**
SEM	75,90	52,55	73,78	86,25	61,25	202,49	182,95	81,96	146,35	132,66	139,08	139,86	48,06	31,16	67,45
P<	n.s.	n.s.	n.s.	n.s.	n.s.	0.01	0.01	0.01	0.01	0.01	0.01	0.01	0.01	0.01	0.01

Tab. 38 Effects of Salidroside 1.50 mg/l on pyramidal cell activity in terms of changes of population spike amplitudes. Results from single slices as obtained after single stimuli (SS) or after burst stimuli (TBS). Overview on final results after averaging 4 slices. SD=standard deviation; SEM=standard error of mean. P<=Wilcoxon Mann Whitny U-Test. ACSF=artificial cerebro-spinal fluid.

NCAG 1517															
slice	ACSF +SS					WS-KSM-66 0.25 mg/l + SS					WS-KSM-66 0.25 mg/l + TBS				
time [min]	10	20	30	40	Mean 20-40	50	60	70	80	Mean 60-80	90	100	110	120	Mean 100-120
1	-708,14	-953,69	-910,18	-896,30	**-920,06**	-992,45	-1232,00	-1289,00	-944,40	**-1155,13**	-1387,00	-2231,00	-1832,00	-2064,00	**-2042,33**
2	-849,73	-1088,00	-1129,00	-996,14	**-1071,05**	-1059,00	-1097,00	-1116,00	-1046,00	**-1086,33**	-1966,00	-2030,00	-2202,00	-2315,00	**-2182,33**
3	-806,49	-1153,00	-1011,00	-947,93	**-1037,31**	-1045,00	-1021,00	-1170,00	-1148,00	**-1113,00**	-1796,00	-2037,00	-2065,00	-2098,00	**-2066,67**
4	-1225,00	-935,58	-1213,00	-1201,00	**-1116,53**	-1135,00	-1222,00	-1257,00	-1497,00	**-1325,33**	-1755,00	-1999,00	-2075,00	-1998,00	**-2024,00**
Mean	-897,34	-1032,57	-1065,80	-1010,34	**-1036,24**	-1057,86	-1143,00	-1208,00	-1158,85	**-1169,95**	-1726,00	-2074,25	-2043,50	-2118,75	**-2078,83**
SD	226,33	105,21	132,77	133,48	**83,98**	58,87	101,92	79,31	240,27	**107,39**	243,76	105,80	154,17	137,26	**71,18**
SEM	113,17	52,60	66,38	66,74	41,99	29,43	50,96	39,65	120,13	53,70	121,88	52,90	77,09	68,63	35,59
P<	n.s.	n.s.	n.s.	n.s.	n.s.	n.s.	n.s.	n.s.	n.s.	n.s.	n.s.	n.s.	n.s.	n.s.	n.s.

Tab. 39 Effects of WS KSM66 0.25 mg/l on pyramidal cell activity in terms of changes of population spike amplitudes. Results from single slices as obtained after single stimuli (SS) or after burst stimuli (TBS). Overview on final results after averaging 4 slices. SD=standard deviation; SEM=standard error of mean. P<=Wilcoxon Mann Whitny U-Test. ACSF=artificial cerebro-spinal fluid.
